# Comparative Study of Organoids from Patient-Derived Normal and Tumor Colon and Rectal Tissue

**DOI:** 10.3390/cancers12082302

**Published:** 2020-08-15

**Authors:** Alba Costales-Carrera, Asunción Fernández-Barral, Pilar Bustamante-Madrid, Orlando Domínguez, Laura Guerra-Pastrián, Ramón Cantero, Luis del Peso, Aurora Burgos, Antonio Barbáchano, Alberto Muñoz

**Affiliations:** 1 Departamento de Biología del Cáncer, Instituto de Investigaciones Biomédicas “Alberto Sols”, Consejo Superior de Investigaciones Científicas (CSIC)-Universidad Autónoma de Madrid (UAM), 28029 Madrid, Spain; albacostales@iib.uam.es (A.C.-C.); afbarral@iib.uam.es (A.F.-B.); pbustamante@iib.uam.es (P.B.-M.); lpeso@iib.uam.es (L.d.P.); abarbachano@iib.uam.es (A.B.); 2 Instituto de Investigación del Hospital Universitario La Paz (IdiPAZ), 28029 Madrid, Spain; lauraguerrap@gmail.com (L.G.-P.); ramon.cantero@salud.madrid.org (R.C.); 3 Centro de Investigaciones Biomédicas en Red-Cáncer (CIBERONC), 28029 Madrid, Spain; 4 Unidad de Genómica, Centro Nacional de Investigaciones Oncológicas (CNIO), 28029 Madrid, Spain; odominguez@cnio.es; 5 Departamento de Patología, Hospital Universitario La Paz, 28029 Madrid, Spain; 6 Unidad Colorrectal, Departamento de Cirugía, Hospital Universitario La Paz, 28029 Madrid, Spain; 7 Centro de Investigaciones Biomédicas en Red-Enfermedades Respiratorias (CIBERES), 28029 Madrid, Spain; 8 Unidad de Endoscopia, Departamento de Digestivo, Hospital Universitario La Paz, 28029 Madrid, Spain; burgos.aurora@gmail.com

**Keywords:** colorectal cancer, stem cells, patient-derived organoids, rectal tumors, vitamin D

## Abstract

Colon and rectal tumors, often referred to as colorectal cancer, show different gene expression patterns in studies that analyze whole tissue biopsies containing a mix of tumor and non-tumor cells. To better characterize colon and rectal tumors, we investigated the gene expression profile of organoids generated from endoscopic biopsies of rectal tumors and adjacent normal colon and rectum mucosa from therapy-naive rectal cancer patients. We also studied the effect of vitamin D on these organoid types. Gene profiling was performed by RNA-sequencing. Organoids from a normal colon and rectum had a shared gene expression profile that profoundly differed from that of rectal tumor organoids. We identified a group of genes of the biosynthetic machinery as rectal tumor organoid-specific, including those encoding the RNA polymerase II subunits POLR2H and POLR2J. The active vitamin D metabolite 1α,25-dihydroxyvitamin D3/calcitriol upregulated stemness-related genes (*LGR5*, *LRIG1, SMOC2,* and *MSI1*) in normal rectum organoids, while it downregulated differentiation marker genes (*TFF2* and *MUC2*). Normal colon and rectum organoids share similar gene expression patterns and respond similarly to calcitriol. Rectal tumor organoids display distinct and heterogeneous gene expression profiles, with differences with respect to those of colon tumor organoids, and respond differently to calcitriol than normal rectum organoids.

## 1. Introduction

Colorectal cancer (CRC) is no longer considered a single disease. The colon and rectum differ in their ontogeny, anatomy, microbiome, and function, and this is reflected in differences in risk factors and epidemiological and clinicopathological features between colon and rectal tumors [1,2,3,4,5]. The two types of cancer also differ in their patterns of metastases and chemosensitivity [6,7,8], and this underlies the different clinical management of colon and rectal cancer patients [3,9]. Moreover, colon and rectal carcinomas have different mutational landscapes and cytogenetic aberrations, as is also the case for tumors affecting different colon segments [10,11,12,13]. This has led to the idea that molecular-genetic features change gradually along the colorectum, known as linearity [1]. Differences in gene expression patterns have been found between a normal colon and rectum and between colon and rectal tumors [14,15]. However, these genetic studies were performed with whole tissue biopsies containing a mix of tumor and non-tumor epithelial and stromal cells, including fibroblasts, immune cells, pericytes, adipocytes, blood, and lymphatic endothelial cells. It has therefore been impossible to assign the expression of a given gene to a particular cell type.

According to the cancer stem cell (CSC) model, tumors have a hierarchical organization, with a minor population of mutated stem cells (CSCs) being responsible for tumor initiation, progression, and, possibly, the capacity to metastasize and regrow after radiotherapy or chemotherapy. In mice, crypt stem cells have been identified as “the cells-of-origin of intestinal cancer” [16]. Our laboratory has therefore performed long-term cultures of intestinal normal stem cells (SC) and CSCs and their progenies in three-dimensional (3D) structures called organoids. These organoid cultures were generated from biopsies obtained endoscopically at diagnosis of therapy-naïve rectal cancer patients, thus generating a living biobank of patient-derived normal and tumor organoids.

The gut physiology is profoundly affected by the action of vitamin D [17], and observational studies have established a link between vitamin D deficiency and an elevated risk and poor prognosis of CRC, suggesting that colon and rectal cancer patients might preferentially benefit from an adequate vitamin D status [18,19,20,21,22,23,24,25,26,27,28]. Data from recent interventional clinical studies indicate that the addition of vitamin D to standard chemotherapy improves the progression-free survival of metastatic CRC patients [29]; however, another randomized trial showed no preventive effect on adenoma recurrence [30]. Long-term studies show that vitamin D compounds inhibit cell proliferation in human rectal mucosa explants and colon carcinoma cells, inducing differentiation of the latter [31,32]. Our recent results show that human colon SCs in situ and organoids derived from normal human colon express the vitamin D receptor and respond to calcitriol, which is the active vitamin D metabolite [33].

In this study, we compared the gene expression profile of SC-derived organoids from human normal colon and rectum tissue and of CSC-derived organoids from rectal tumors. Additionally, we compared the gene expression profiles to the transcriptomic data previously obtained from CSC-derived organoids from colon tumors [33]. Our study shows that human SC-derived organoids from a normal colon and rectum have highly similar morphology and gene expression profiles, whereas they differ greatly between organoids derived from a normal rectum and rectal tumors. This is reflected in the expression of distinct sets of genes abnormally expressed in rectal and colon tumor organoids vs. their respective normal counterparts, including a number of genes of the protein biosynthetic machinery. We also found that normal colon and rectum organoids respond very similarly to calcitriol, whereas the calcitriol responses of a normal rectum and rectal tumor organoids are different. To the best of our knowledge, this is the first report comparing human normal and tumor colon and rectum organoids and the effects of calcitriol on normal and tumor organoids derived from these tissues.

## 2. Results

### 2.1. Establishment and Morphology of 3D Organoids From Endoscopic Biopsies from Rectal Cancer Patients

We generated a biobank of organoids (*n* = 50) from therapy-naïve rectal cancer patients subjected to screening colonoscopy. The minimum amount of biopsy material plus the bacterial contaminations limited the success rate of organoid establishment to 74% (at least one type of organoid culture from 50 out of 68 patients), which is very similar to recent reports [34,35]. Although the small size of endoscopy samples made it difficult to establish stable cultures of the normal colon, normal rectum, and rectal tumor from the same patient, the efficiency establishment was 85% for normal organoids (colon and rectum) and 55% for tumor organoids. The gene expression profiles of colon, rectum, and rectal tumor organoids were analyzed by RNA-sequencing (RNA-seq). Patient characteristics are shown in Table 1. Normal colon and normal rectum organoids were obtained from the same rectal cancer patients (#40, #41, #42, #43, #44, and #45). However, the rectal tumor organoids were established from a different patient group (#44, #46, #47, #48, #49, and #50), because some rectal tumors were stenosing and clogged the endoscopy track, blocking access to the normal tissue and making it impossible to get a biopsy from the colon, rectum, and rectal tumor from the same patient. For some comparative analyses, we also included previously reported data from normal and tumor colon organoids (obtained from surgical biopsies) [33], meaning that data for the normal colon is *n* = 12 when the published data is included (indicated in the figures).

**Figure 1:**
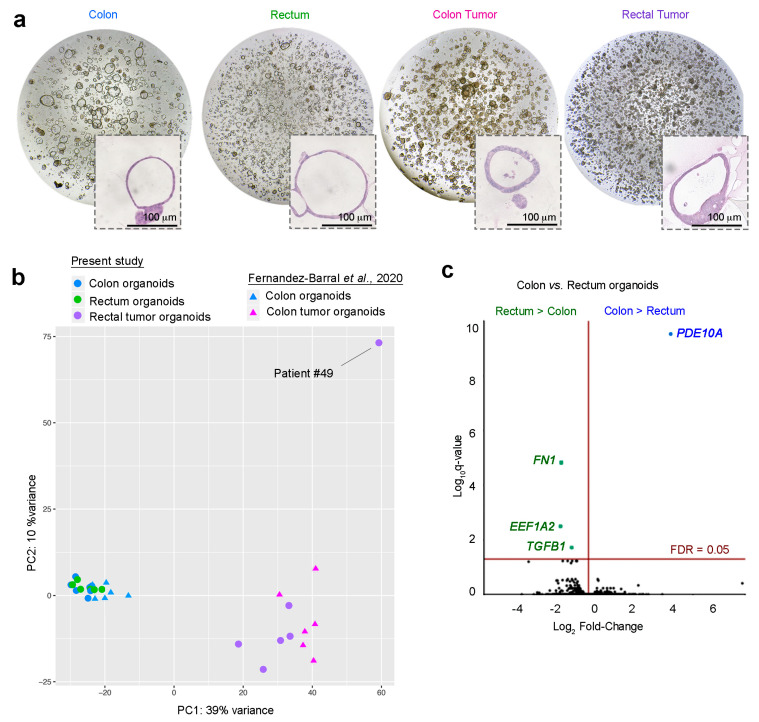
Morphology and gene expression analysis of patient-derived normal and tumoral colon and rectum tissues. (**a**) Phase-contrast micrographs of each type of patient-derived organoid culture. Insets show hematoxylin and eosin (H&E) staining. Scale bar = 100 μm. (**b**) Principal component analysis (PCA) plot of the transcriptomes of organoid cultures derived from the colon, rectum, rectal tumor, and colon tumor. Additional RNA-seq data for normal and tumor colon organoids were included from a previous publication (accession number GSE100785) [33]. (**c**) Volcano plot comparing human colon and rectum RNA-seq signatures from six matched normal organoid cultures. The *x*-axis shows Log_2_ fold-change (Log_2_FC) and the *y*-axis shows the *q*-value (−Log_10_).

Rectal tumor organoids were derived from all rectal sites (distal, mid, and upper). Whereas normal colon and rectum organoids were dependent on Wnt3a, R-Spondin, and nicotinamide, rectal tumor organoids (such as colon tumor organoids) could be grown in the absence of these factors. Although the precise 3D morphology of each patient-derived normal colon and rectum organoid culture was unique, normal organoids were generally composed of a single cell monolayer surrounding a large lumen, whereas tumor organoids consisted of several cell layers surrounding a smaller and more variably sized lumen (Figure 1a). 

### 2.2. Gene Profiling of Normal Colon and Normal Rectum Organoids

To compare the gene expression profiles of normal colon and rectum SC-derived organoid cultures, we performed an RNA-seq analysis on six matched (same patient) organoids from both normal tissues. Principal component analysis (PCA) and hierarchical clustering of samples based on the Euclidean distance of normalized RNA-seq counts showed that normal organoids from the colon and rectum clustered together, whereas tumor organoids from the colon or rectum had a more heterogeneous and dispersed transcriptomic profile and clustered far from the normal organoid group (Figure 1b). Normal colon and rectum organoids had very similar gene expression profiles, with only four genes being differentially expressed above the 5% false discovery rate (FDR) threshold (Figure 1c): In colon organoids, the only gene with a statistically higher expression was *PDEA10A*, and rectum organoids showed an elevated expression of *FN1*, *TGFB1*, and *EEF1A2*. *PDE10A* encodes the phosphodiesterase isoenzyme 10A, which degrades cAMP and cGMP. PDE10A promotes β catenin nuclear translocation and transcriptional activity and proliferation in colon carcinoma cells and is overexpressed in colon tumors [36]. *FN1*-encoded fibronectin 1 is more highly expressed in the normal rectum than in the normal right colon in patients with Lynch syndrome [15]. Curiously, *FN1* and *TGFB1* are upregulated by 5-fluorouracil in colon carcinoma cells [37]. Recently, the *EEF1A2*-encoded eukaryotic elongation factor 1 alpha 2 (eEF1A2), which is a translation elongation factor with additional moonlighting functions, was reported to be overexpressed in many cancers and to promote tumor cell survival [38,39]. eEF1A2 is a target of the natural marine antitumor drug Plitidepsin [40].

### 2.3. Gene Profiling of Normal Rectum and Rectal Tumor Organoids

**Figure 2:**
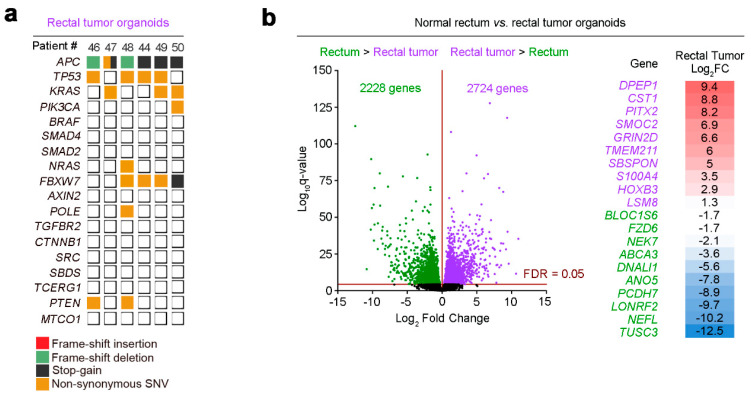
Mutational analysis and transcriptome profiles of human rectal tumor organoids. (**a**) Overview of the mutations found in the rectal tumor organoid cultures of six patients. (**b**) Volcano plot comparing normal rectum and rectal tumor RNA-seq signatures from six organoid cultures. The *x*-axis shows Log_2_FC and the *y*-axis shows the *q*-value (−Log_10_). Green/purple dots represent downregulated/upregulated (respectively) genes in rectal tumor organoids. The table lists the top 10 (lowest *q*-value) upregulated/downregulated genes in rectal tumor organoids ordered by their Log_2_FC.

We compared the transcriptomes of six normal rectum and rectal tumor organoid cultures by RNA-seq. Mutational analysis of rectal tumor organoids confirmed that they harbored patterns of genetic alterations that are typical of sporadic CRC, with mutations in *APC* (6/6), *TP53* (4/6), and *KRAS* (3/6) (Figure 2a). This mutation pattern is similar to that previously reported for colon tumor organoids: *APC* (5/6), *TP53* (3/6), and *KRAS* (4/6) [33].

PCA showed robust clustering and strong separation between organoids derived from normal tissue and those from rectal tumor tissue (Figure 1b). Notably, the rectal tumor organoid culture from patient #49 was very separated in comparison to all others. The explanation for this finding came from a post-surgery pathological study that revealed patient #49 tumor as neuroendocrine, which led us to exclude its organoids from gene expression analyses. The comparison of normal rectum and rectal tumor organoids detected 4952 differentially expressed genes (FDR-adjusted *p* < 0.05): 2228 downregulated genes in rectal tumor organoids and 2724 upregulated genes (Figure 2b). The top-10 dysregulated genes are listed in Figure 2b.

### 2.4. Transcriptomic Profiles of Rectal Tumor and Colon Tumor Organoids

**Figure 3:**
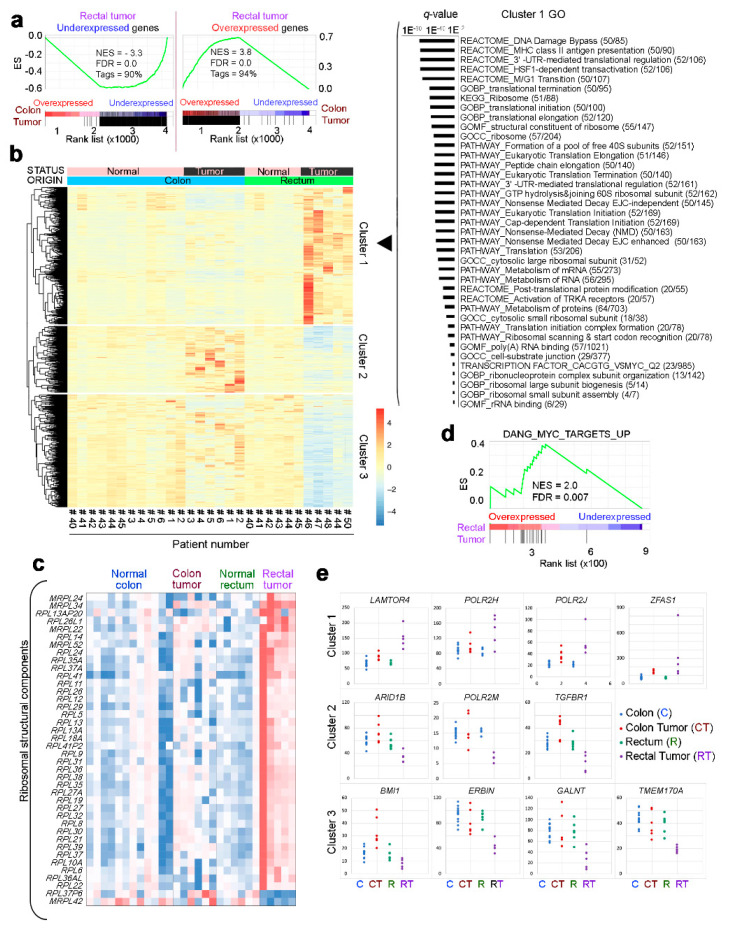
Transcriptomic comparison of colon and rectal tumor organoids. (**a**) Gene Set Enrichment Analysis (GSEA) comparing the genes dysregulated in colon and rectal tumor organoids. (**b**) Heatmap showing the genes that behave distinctly during tumor progression in the two locations (FDR 5%). Clusters 1–3 group genes whose expression changes distinctly in colon and rectal tumors. The list to the right shows Gene Ontology analysis of Cluster 1 genes (only induced in rectal tumors) in several databases. (**c**) Heatmap showing the expression (cpm) of genes encoding ribosomal structural components distinctly dysregulated in colon and rectal tumor organoids (interaction study, FDR 5%). (**d**) GSEA comparing the MYC signature with the genes distinctly dysregulated in rectal tumor vs. colon tumor organoids. (**e**) Quantification of the expression level (cpm) of selected genes from Clusters 1 to 3 in (**b**).

A Gene Set Enrichment Analysis (GSEA) comparison of transcriptomic changes in rectal tumor organoids and those reported in colon tumor organoids [33] revealed that most defining gene sets are common to both types of tumor (Figure 3a). However, some dysregulated genes showed different responses in colon and rectal tumor organoids. To determine whether these differences were significant, we looked for interactions between the variable status (normal vs. tumor) and origin (rectum vs. colon). This analysis revealed 905 genes whose expression upon malignant transformation differed significantly (FDR < 0.05), depending on the tissue of origin (Figure 3b). These genes could be broadly divided into three clusters: Cluster 1, including genes upregulated upon tumor transformation in the rectum but not in the colon; cluster 2, including genes upregulated in the colon tumor but downregulated in the rectal tumor; and cluster 3, including genes downregulated in the rectal tumor but little affected in the colon tumor (Figure 3b).

Regarding cluster 1, Gene Ontology analysis of multiple databases showed that the rectal–cancer-specific genes are related to protein synthesis: Ribosomes, translation control, protein metabolism, and RNA (Figure 3b). Further analysis confirmed a large set of ribosomal genes only upregulated in the rectal tumor (Figure 3c). Ribosome biogenesis is directly regulated by *c-MYC* [41,42], and GSEA showed that the genes specifically overexpressed in rectal tumors are targeted by c-MYC (Figure 3d). c-MYC associates with polymerases I, II, and III to produce pre-rRNA and ribosomal protein subunits [43]. We observed that *POLR2H* and *POLR2J* are exclusively overexpressed in rectal tumor organoids (Figure 3e). *POLR2H* encodes the common H subunit of RNA polymerases I, II, and III [44] and *POLR2J* encodes a core subunit of the polymerase II that interacts with transcription factors [45] and translation initiation factors [46]. On the contrary, the same organoids show a downregulated expression of *POLR2M*, which encodes the RNA polymerase II Gdown1 repressor [47].

The three clusters of genes showing expression changes upon malignant transformation included several known cancer-related genes. For example, rectal tumor organoids showed a diminished expression of the colorectal tumorigenesis suppressor *ERBIN7* [48] and increased expression of the mTORC1 activator *LAMTOR4* [49] and the protumorigenic gene *ZFAS1* [50] (Figure 3e).

### 2.5. Effect of Calcitriol on Organoids from Normal and Tumor Tissues

**Figure 4:**
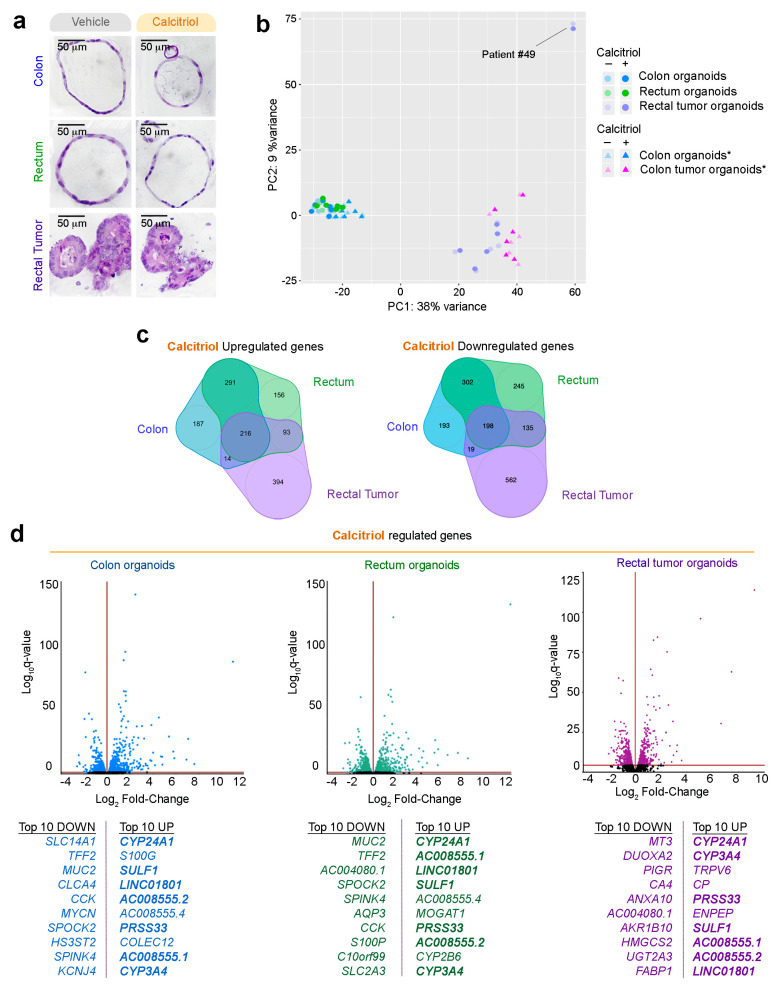
Effect of calcitriol on human normal colon, rectum, and rectal tumor organoids. (**a**) Morphology analysis by H&E staining of each type of patient-derived organoids cultured for 96 h in the presence of 100 nM calcitriol or vehicle. Scale bar = 50 µm. (**b**) PCA of raw RNA-seq data from organoids treated with calcitriol or vehicle (17 patients/30 organoid cultures). * RNA-seq data (six patients) for normal and tumor colon organoids were included from a previous publication; transcriptome data from GSE100785 [33]. (**c**) Venn diagram showing the overlap among genes showing a significantly altered expression in normal and tumor organoids. The numbers of genes in each group are indicated. (**d**) Volcano plots showing calcitriol-regulated genes in organoids derived from a normal colon (blue), normal rectum (green), and rectal tumor (purple). Top-10 up/down regulated genes in normal colon, normal rectum, and rectal tumor organoids are listed. Common regulated genes in the three types of cultures are shown in bold.

Given the protective action attributed to vitamin D in CRC, we next studied the effect of its active metabolite calcitriol on the transcriptomes of organoids derived from a normal colon, normal rectum, and rectal tumors. To investigate the long-term effects of calcitriol, we treated the organoids with calcitriol (100 nM) or vehicle for 96 h and performed RNA-seq analysis (patients #40 to #50). Calcitriol did not alter the morphology of normal colon or rectum organoids (monolayer, large lumen) or rectal tumor organoids (multilayer, irregular, small or no lumen) (Figure 4a).

PCA showed that calcitriol promoted a moderate change in the global transcriptome (Figure 4b). Again, patient #49′s rectal neuroendocrine tumor organoid culture behaved very distinctly and was excluded from further analyses. Calcitriol regulated comparable numbers of genes in the colon (1420), rectum (1636), and rectal tumor organoids (1631) (Figure 4c, Table S1). Venn diagrams show that only a minor subset of genes were commonly upregulated (216) or downregulated (198) in all three organoid types (Figure 4c). This finding is consistent with the results of an in vivo study on the response of human white blood cells to vitamin D, which revealed significant individual differences in vitamin D target genes [51]. Nevertheless, in our analysis, eight of the top-10 calcitriol-upregulated genes and five of the top-10 downregulated genes coincided with normal colon and rectum organoids (Figure 4d). In contrast, in rectal tumor organoids, seven of the top-10 upregulated genes and none of the downregulated genes coincided with the normal organoid types.

The most strongly upregulated gene by calcitriol in all organoid types was *CYP24A1*, as also occurs in many other systems (Figure 4d). The other upregulated genes in the top-10 for all three organoids were *CYP3A4* (a key enzyme for drug metabolism), the serine protease *PRSS33*, *AC008555.1*, *AC008555.2*, and *SULF1*. SULF1 is a multitask protein with roles at the plasma membrane and cell nucleus that has both tumor suppressor and promotor actions [52,53,54].

**Figure 5:**
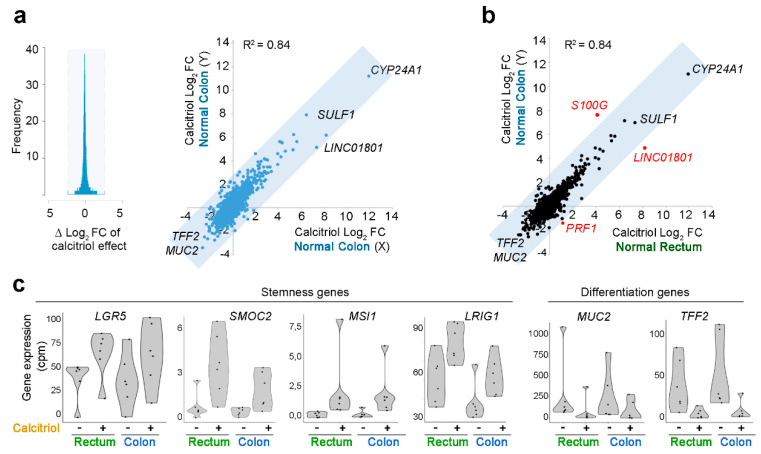
Gene expression profiles induced by calcitriol in normal-tissue organoids. (**a**) Comparison of the calcitriol response of normal colon organoids in independent RNA-seq studies under the same conditions (Normal Colon X: *n* = 6, GSE100785 [33]; Normal Colon Y: *n* = 6, present study; total *n* = 12). Gray shading shows the calculated “correlation zone” according to the dispersion of the Log_2_FC induced by calcitriol in the replicate studies. (**b**) Comparison of the calcitriol response of normal colon organoids and normal rectum organoids. Genes in black within the gray-shaded correlation zone are not considered differentially regulated because the Log_2_FC difference does not exceed that of the replicate assays presented in panel (**a**); genes in red outside of the correlation zone are considered differentially regulated by calcitriol. (**c**) Violin plots showing the RNA expression (cpm = “counts per million”) of stemness and differentiation genes in colon and rectum organoids and their regulation by calcitriol.

To test the consistency of data for organoids of the same type, we analyzed the data dispersion of the two independent studies of calcitriol-treated normal colon organoids (this study and Ref. [33]; six organoid cultures/study, total *n* = 12). This analysis established a “correlation zone” as the maximum deviation from a 0 difference in Log_2_ fold-change (Log_2_FC) that can be attributed to inter-assay variation among replicates (gray-blocked area in Figure 5a, left). Considering genes outside this “correlation zone” as differentially regulated, we found a good correlation (R^2^ = 0.84) between the RNA-seq results for normal colon organoids in the two independent studies (Figure 5a, right).

Further analyses examined the correlation between the calcitriol responses of different organoid types. The calcitriol responses of normal colon and normal rectum organoids were very similar (R^2^ = 0.87), with three differentially regulated genes: *S100G, LINC01801*, and *PRF1*. *S100G* encodes a calcium binding protein and was more strongly induced (>8 fold-change) in normal rectum than in normal colon organoids (Figure 5b). Calcitriol upregulated the expression of stemness-related genes (*LGR5*, *LRIG1, SMOC2,* and *MSI-1*) in normal rectum organoids, while it downregulated differentiation marker genes (*TFF2* and *MUC2*) (Figure 5b,c); this coincides with the data from colon organoids [33] and suggests that calcitriol supports crypt stem-cell maintenance in the colon and rectum.

**Figure 6:**
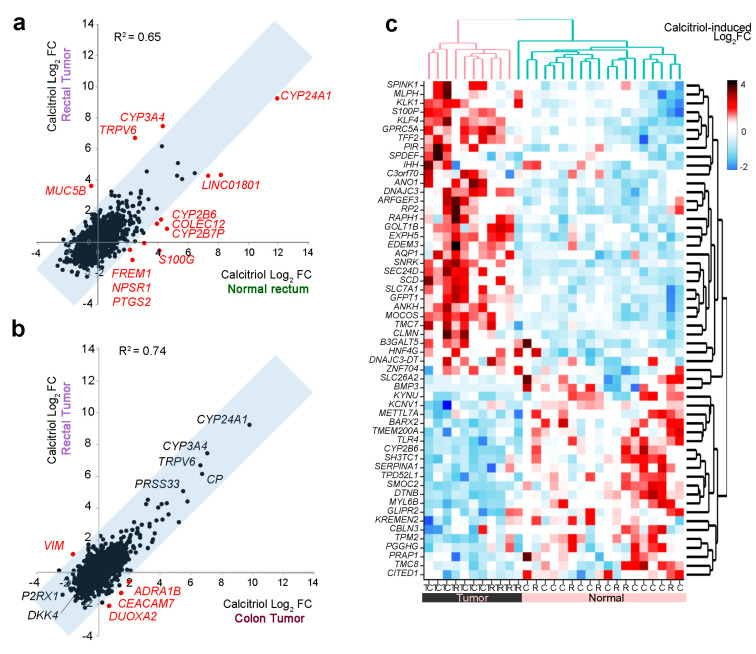
Comparison of calcitriol effects on the gene expression profiles of rectal tumor and normal rectum organoids. (**a**) Comparison of the calcitriol response of rectal tumor and normal rectum organoids (*n* = 6 of each type). Genes showing differential regulation between the two organoid types are shown in red. (**b**) Comparison of the calcitriol response in colon tumor and rectal tumor organoids (*n* = 6 of each type). Genes in black within the gray-shaded correlation zone are not considered differentially regulated because the Log_2_FC difference does not exceed that of the replicate assays presented in Figure 5a. (**c**) Heatmap showing genes differentially regulated by calcitriol in normal and tumor organoids. The heatmap shows calcitriol regulation (Log_2_FC) in the transcriptome of 29 organoid cultures (colon, *n* = 12; colon tumor, *n* = 6; rectum, *n* = 6; rectal tumor, *n* = 5). The RNA-seq data for six of the normal colon organoid cultures and the six colon tumor organoid cultures were obtained from GSE100785 [33].

A comparison of calcitriol-treated normal rectum and rectal tumor organoids revealed several distinctly regulated genes (Figure 6a). A single gene was induced by calcitriol in rectal tumor organoids, but repressed in normal rectum organoids (*MUC5B*), while the opposite regulation was found for *S100G, FREM1, NPSR1,* and *PTGS2.* In addition, *COLEC12*; *LINCO810*; and the metabolizing enzymes *CYP24A1*, *CYP2B7P*, and *CYP2B6* were induced to a lesser degree in rectal tumor organoids, whereas *TRPV6* and *CYP3A4* were more strongly induced by calcitriol in rectal tumor organoids (Figure 6a).

We also compared the calcitriol response of rectal tumor organoids to that of colon tumor organoids, reported recently [33] (Figure 6b). The calcitriol-induced transcriptional profiles were very similar in the two types of organoids, with the calcitriol-induced Log_2_FC in colon tumor organoids (*n* = 6) mirroring that in the rectal tumor organoids (*n* = 6). The top calcitriol-upregulated genes in both types of organoids were the known calcitriol targets *CYP24A1*, *CYP3A4*, *TRPV6,* and *PRSS33*. Among the top-downregulated genes, we found *P2RX1* and *DKK4,* with the latter described as repressed by calcitriol in colon carcinoma cells [55,56]. *DUOXA2*, *CEACAM7*, and the proto-oncogene *ADRA1B* [57] were downregulated by calcitriol in rectal tumor organoids, but upregulated in colon tumor organoids. On the contrary, the pro-migratory and EMT-related gene *VIM* [58] was upregulated by calcitriol in rectal tumor organoids, but downregulated in colon tumor organoids (Figure 6b). Moreover, a large group of genes previously reported as differentially regulated by calcitriol in normal vs. tumor colon organoids [33] showed the same type of regulation in normal rectum and rectal tumor organoids (Figure 6c).

Lastly, we examined the effect of calcitriol treatment on the four genes (*FN1*, *EEF1A2*, *PDE10A,* and *TGFB1*) differentially expressed between normal rectum and normal colon organoids (Figure 1c). Calcitriol significantly upregulated *FN1* in normal colon, rectum, and rectal tumor organoids. Conversely, it downregulated *EEF1A2* in normal rectum organoids, *PDE10A* in normal colon organoids, and *TGFB1* in rectal tumor organoids (Table S2).

## 3. Discussion

In the present work, we describe the generation and gene expression profiling of organoids derived from endoscopic biopsies of a normal colon and rectum and from rectal tumors of therapy-naïve patients at diagnosis. We also compare the effect of the active vitamin D metabolite calcitriol on the gene expression profiles of organoids derived from normal and tumor rectum and colon tissue, including data from the organoids generated here and others derived from human colon tumors characterized in a previous report [33]. A few previous studies have described the generation of rectal tumor organoids from patients with primary, metastatic, or recurrent disease and who had received a variety of treatments [34,59,60] or were therapy-naïve [35]. These earlier studies examined the ex vivo responses of rectal tumor organoids to chemotherapy and/or radiation and showed that rectal cancer patient-derived organoids can predict clinical responses to therapy. Our study pioneers the characterization of normal colon and rectum tissues by comparing derived organoids in culture. We also compared the transcriptomes of organoids derived from a normal rectum and therapy-naïve rectal tumors and their responses to calcitriol.

Our results show extremely similar gene expression profiles for organoids derived from normal colon and rectum stem cells. This was unexpected given the reported differences between these large intestine segments in terms of function, oncogenic transformation, and response to therapy. A possible explanation is that the colon biopsies were from the sigmoid colon, which in many respects is more similar to the rectum (including hindgut derivation and vascularization) than to the right colon [3]. These similarities are reflected in the closely overlapping mutational landscape of distal left colon and rectal tumors, which differs from that of right colon tumors [12]. Our data do not reproduce the reported differences in gene expression between the sigmoid colon and rectum epithelial gene signatures (obtained after elimination of the stromal layer by vortexing) of normal participants in a dietary study of gut resiliency [61]. A comparison of right colon organoids and rectum organoids is an interesting proposition, but difficult to address via screening colonoscopy. Another, not exclusive, explanation for the discrepancy between our results and the differences between the colon and rectum in vivo is that organoid cultures cannot reproduce organismal signals, derived either from soluble/long distance signalers or from interactions between cells (epithelial stem cells and stromal cells) or between cells and the tissue matrix. Qualitative and quantitative differences in such local signals may underlie heterogeneity between colon and rectum tissues in vivo that is lost in their respective cultured stem cells. Other studies have described gene expression differences between human fetal small and large intestine stem cells [62], human duodenum and ileum organoids [63], and human fetal colonic segments [64]; however, the reported differences were small, and the statistical criteria used were sometimes less restrictive than those used in the present study. If confirmed, the similarity between normal sigmoid colon and rectum stem cells would have implications for regenerative medicine, potentially making these stem cells interchangeable in transplantation approaches to distal intestinal diseases.

The many differences detected between the gene expression profiles of rectal cancer and normal rectum tissue organoids matches data from colon tumor and normal colon organoids [33,59] and is in line with the large number of alterations in rectal tumors detected by large-scale sequencing [65]. Interestingly, although there was extensive overlap between genes aberrantly expressed in rectal and colon tumor organoids, some of them showed differences in the level of expression, while others showed opposite dysregulation in the two tumor organoid types. Importantly, a group of genes involved in protein synthesis (ribosomal biogenesis, translation components, and regulation) was over-expressed in rectal, but not colon, tumor organoids, suggesting that colon and rectal tumors may differ more at the level of protein expression than at the RNA level. A multifaceted relation has been proposed between ribosome biogenesis and cancer, in part derived from the over-activation of this process in cancer cells [66]. Moreover, a recent study by Batlle’s group has revealed that a subset of tumor cells that are adjacent to the stroma have a high biosynthetic capacity, with a strong expression of genes involved in protein synthesis, and are at the top of the stem cell hierarchy in CRC [67]. In both our study and that of Batlle’s group, genes encoding subunits of RNA polymerases were found; however, while we detected differences in the expression of three RNA polymerase II subunits (POLR2H, POLR2J, and POLR2M, with the first common to all three RNA polymerases) between rectal and colon tumor organoids, Batlle’s group referred to elevated levels of RNA polymerase I subunit A (POLR1A) as a critical feature of top CSC in CRC. Together, these data support the interest in a profound analysis of ribosomal DNA transcription and protein synthesis to define the perhaps distinct stem cells that sustain colon and rectal tumors.

Given the highly overlapping gene profiles of untreated normal colon and rectum organoids, the close similarity of the calcitriol responses of these organoids is not surprising. Calcitriol thus appears to have the same effect on stem cells from the sigmoid colon and rectum. In addition, differences in the effect of calcitriol on the transcriptome of normal rectum and rectal tumor organoids were mainly on the level of gene expression. These findings appear to conflict with the reported links between genomic variation/polymorphisms in several vitamin D system genes (*VDR*, *GC*, *CYP24A1*, *CYP2R1,* and *DHCR7/NADSYN1*) and differences in the response to vitamin D compounds in studies involving CRC patients [68,69,70,71,72,73]. However, this discrepancy may again reflect differences between the in vivo and in vitro situations, since some vitamin D system genes play no role in cultured organoids; for example, the *GC* gene encodes the vitamin D binding protein, which is responsible for the blood transport of 25-hydroxyvitamin D/calcidiol and calcitriol.

The highly similar effect of calcitriol on colon tumor and rectal tumor organoids is consistent with the finding that colon and rectal cancers, excluding hypermutated cancers, have similar patterns of genetic alterations [65]. Moreover, our finding also agrees with the proposed protective effect of vitamin D on both colon and rectal cancers [18,19,20,21,22,23,24,25,26,27,28].

Common concerns in the analysis of calcitriol action are the interindividual variability inherent to patient-derived organoids and the acquired genetic instability described in several studies [74,75,76]. These factors may preclude the identification of statistically significant differences in target genes, and therefore quantitative effects and strong tendencies that do not reach statistical significance should not be automatically discarded as irrelevant.

The data presented here show that organoid cultures generated from human adult sigmoid colon and rectum stem cells share a highly similar pattern of gene expression, with only four exceptions identified as differentially expressed genes. Differences between stem cells from rectum and other colon segments remain to be determined. The gene expression responses of normal colon and rectum organoids to calcitriol show only minor differences. Interestingly, calcitriol upregulates stemness genes in rectum and colon organoids, consistent with a homeostatic action on the large intestine crypt stem cell compartment.

The gene expression patterns of rectal tumor organoids compared to normal rectum organoids are generally distinct, and the differences between rectal tumor and colon tumor organoids related to the biosynthetic capacity suggest that the malignant transformation of stem cells might differ according to tumor origin.

## 4. Materials and Methods

### 4.1. Human Samples and Ethical Guidelines

Fresh human tissues were provided by IdiPAZ, a participating center in the Spanish Biobank Network. The biopsy samples were obtained from individuals undergoing screening colonoscopy. Informed consent was obtained from all participants. The study was approved by the Hospital Universitario La Paz Ethics Committee (HULP-PI-1425 and HULP-PI-1639) and also complied with Helsinki declaration ethical guidelines.

### 4.2. Establishment of 3D Organoid Cultures from Endoscopy Biopsies

Normal rectum mucosa samples were collected with 6 mm forceps from the area adjacent to the rectal tumor, either distal or proximal. Normal colon samples were collected from the sigmoid colon sigma mucosa. Normal colon/rectum biopsies were incubated with rotation in phosphate buffered saline (PBS) and antibiotics (Primocin (Invivogen, CA, USA) plus gentamycin and fungizone (Thermo Fisher Scientific, MA, USA)) for 30 min at room temperature (RT). After cutting tissues into small pieces, they were incubated for 2 × 5 min in PBS containing 10 mM dithiothreitol (DTT) at RT, followed by two incubations in 8 mM EDTA in PBS (first for 5 min at RT and second 60 min with slow rotation at 4 °C). EDTA was removed by several PBS washes and samples were transferred to a 50 mL conical tube in fresh PBS. In order to separate crypts from the mucosa, tubes were gently shaken, and supernatants were centrifuged at 200× *g* for 5 min at RT to pellet crypts. We used a specific washing buffer (Table 2) to wash the crypts and they were finally embedded in Matrigel (Corning, MA, USA). We seeded the drops in pre-warmed (overnight) 48-well culture dishes. Complete “normal” culture medium (Table 2) was added after Matrigel solidification.

Human rectal tumor organoid cultures were generated as follows. Human tumor endoscopy biopsies were washed several times in PBS and incubated with antibiotics (as normal biopsies) for 30 min at RT. Biopsies were cut into small pieces and digested with collagenase type IV (1 mg/mL in PBS (Sigma-Aldrich)) for 30 min at 37 °C with continuous shaking in a water bath. In order to obtain single cells, they were disaggregated by passing the suspension through a 18 G needle and the cell suspension was filtered through a 200-μm mesh into a 50 mL conical tube and centrifuged at 250× *g* for 5 min at 4 °C. Cells were incubated in 157 mM NH_4_Cl for 5 min to lysate the erythrocytes, washed in washing buffer, and finally embedded in Matrigel and seeded on pre-warmed 48-well culture dishes. “Tumor” culture medium (“normal” culture medium minus Wnt3a-conditioned medium, nicotinamide, and RSPO1) was added after Matrigel solidification.

### 4.3. Growth and Expansion of Organoid Cultures

Normal and tumor culture medium was changed every second day. Cells were passaged according to the protocol previously published [33]. Briefly, after collecting organoids with a scraper in a 15 mL conical tube, they were incubated with Cell Recovery Solution (Corning) for 30 min on ice with continuous shaking to remove Matrigel. After two washes (washing buffer) and centrifugations at 260× *g* for 5 min at 4 °C, organoids were incubated in disaggregation buffer (washing buffer containing 1 mg/mL dispase (Thermo Fisher Scientific)) for 10 min at RT with orbital rotation, and additional incubation for 5 min after adding 2 mM EDTA to the mixture. Organoids were disaggregated by passing through a 21G needle and pelleted by centrifugation at 250× *g* for 5 min at 4 °C. After washing the cell solution twice in washing buffer, pelleted cells were embedded in Matrigel and seeded on culture dishes.

### 4.4. Histology and Imaging

Culture medium was removed, and after washing the organoids (passages 3–7) twice in PBS, they were collected in a Tissue-Tek Paraform biopsy cassette (Sakura, The Netherlands). Hematoxilin/eosin staining was performed in 3 μm paraffin sections. Images were captured with a DFC550 digital camera (Leica, Wetzlar, Germany) mounted on an inverted TS100 microscope (Nikon, Tokyo, Japan).

### 4.5. Mutational Status

Mutations were analyzed in six tumor organoid cultures from rectal cancer patients (#44, 46–50; passages 1–5). Cell Recovery Solution was used to remove Matrigel, and DNA was extracted from pelleted organoids by incubation overnight at 56 °C in lysis buffer (Table 2) for DNA extraction. After adding saturated NaCl buffer for five minutes, DNA was precipitated with isopropanol and washed twice with 70% ethanol. DNA was genotyped by sequencing the amplified product of a multiplexed-PCR reaction (Amplicon sequencing) using a proof-reading polymerase. Indexed libraries were pooled and loaded onto an MiSeq instrument (Illumina, CA, USA). Initial alignment was performed with BWA after primer sequence clipping, and variant calling was done with the GATK Unified Genotyper and VarScan2, followed by ANNOVAR annotation. Mutations were called at a minimum 3% allele frequency. Single nucleotide polymorphisms (SNPs) were filtered out with dbSNP and 1000 genome datasets. All detected variants were manually revised and confirmed.

### 4.6. RNA-Sequencing

Normal (passages 2–4) and tumor organoids (passages 2–11) from 11 patients (#40–50, Table 1) were seeded in 48-well culture dishes and 48 h later, were treated with 100 nM calcitriol (Sigma-Aldrich) or a vehicle for 96 h. “Normal” or “tumor” medium and calcitriol/vehicle was replaced every second day. Total RNA was extracted, and an optimal RNA integrity was confirmed with an Agilent 2100 Bioanalyzer (RIN range, 9.1–9.9). Following this, 600 ng RNA samples were used to construct the cDNA library with the Illumina TruSeq Stranded mRNA Sample Preparation kit (Illumina). The adapter-ligated was sequenced on an Ilumina HiSeq2000 apparatus according to the manufacturer’s protocols, and a minimum of 25 million 50-base single-reads were generated for each sample.

Sequencing reads were aligned to the transcriptome with TopHat2 [77]. Novel transcript discovery was not attempted. TopHat was provided with known gene annotations and other transcript data obtained from the Gencode basic gene set (Version 26—Ensembl 75) for the GRCh37/hg19 human genome assembly. The gene expression level was calculated from TopHat alignments as the number of reads per gene computed using HTSeq with default settings and gene features, as defined in the GRCh37.75 human genome release. Differential gene expression analysis was performed with the Bioconductor DEseq2 package [78] for R statistical software (https://www.r-project.org). Venn diagrams were made using nVenn [79].

### 4.7. Coefficient of Determination and Correlation Zone

Inter-assay variability was calculated by comparing two identical studies. Differences in calcitriol-induced Log_2_FC between the present study and Ref. [33] (Figure 5a, left) reached maximum values of 2.6 (absolute value ΔLog_2_FC). This interval was interpreted as the “correlation zone” and is highlighted in gray in the figures. Genes differentially regulated by calcitriol were defined as those with |ΔLog_2_FC| > 2.6. The coefficient of determination (R^2^) was calculated using Excel’s built-in formula.

### 4.8. Gene Set Enrichment Analysis (GSEA)

The ranked list used in Figure 3a comprises Log_2_FC ordered genes significantly over- or under-expressed in rectal tumor organoids relative to normal rectum organoids. The comparative dataset comprises genes from GSE100785 that were significantly over- or under-expressed in colon tumor organoids relative to normal colon organoids. The ranked list in Figure 3d includes genes with different expression in rectal tumor organoids compared to colon tumor organoids (Log_2_FC ordered); this dataset was obtained from the status/origin interaction study (FDR < 0.05). A comparative gene set used for Figure 3d is named “DANG_MYC_TARGETS_UP” in the GSEA database.

### 4.9. Data Availability

RNA-seq analysis data for patient-derived rectal tumor and normal colon and rectum organoids related to this study have been deposited in the Gene Expression Omnibus (GEO) under the accession code GSE143782. For the purpose of comparison, the present study also analyzed transcriptomes from calcitriol- and vehicle-treated organoids from patients #1–6 in Ref. [33] (GSE100785).

## 5. Conclusions

In conclusion, normal sigmoid colon and rectum organoids have similar gene expression profiles and calcitriol responses. In contrast, rectal tumor organoids display distinct patterns of gene expression that also differ from those of colon tumor organoids, and show different behavior to calcitriol than normal rectum organoids.

## Figures and Tables

**Table 1:** Clinicopathological characteristics of rectal tumor patients from whom biopsies were obtained to generate organoid cultures.

Patient	Gender	Age (y)	Sample	T ^a^	N ^b^	M ^c^
#40	Female	65	Normal: Colon + Rectum	pTis	pN0	cM0
#41	Male	88	Normal: Colon + Rectum	cT3, ypT0	cN2a, ypN0	cM1a
#42	Male	70	Normal: Colon + Rectum	cT2-3, ypTis	cN1a, ypN0	cM0
#43	Male	64	Normal: Colon + Rectum	pT4b	pN0	cM0
#44	Male	65	Normal: Colon + Rectum Tumor: Lower rectum	cT3, ypT2	cN2, ypN0	cM0
#45	Male	72	Normal: Colon + Rectum	cT3, ypT3	cN2, ypN0	cM0
#46	Male	69	Tumor: Lower rectum	cT3, ypT2	cN1-2, ypN0	cM0
#47	Male	88	Tumor: Lower rectum	cT3-4, ypT3	cN1, ypN0	cM0
#48	Female	69	Tumor: Medial rectum	cT4	cN2b	cM1
#49	Female	51	Tumor: Upper rectum *	NA	cN+	cM1
#50	Female	88	Tumor: Upper rectum	pT3	pN1b	cM0

For comparative purposes, patients #1–6 from Ref. [33] (GSE100785) were also used (normal colon and colon tumor organoids). T ^a^: Direct extent of the primary tumor; N ^b^: degree of spread to regional lymph nodes; M ^c^: presence of distant metastasis (www.uicc.org/resources/tnm); p: pathological staging on surgical specimen; c: clinical staging based on imaging when no surgical specimen was available; yp: pathological staging after neoadjuvant treatment. * Small cell neuroendocrine carcinoma, excluded from differential expression analysis. #: patient number.

**Table 2:** Medium and buffers used for organoid culture and DNA extraction.

Buffer	Reagent	Concentration	Supplier
Washing buffer	Advanced DMEM/F12	100%	Thermo Fisher Scientific
	Hepes	10 mM	Thermo Fisher Scientific
	Glutamax	10 mM	Thermo Fisher Scientific
Normal culture medium	Advanced DMEM/F12	50%	Thermo Fisher Scientific
	Wnt3a-Conditioned medium	50%	Previously described [33]
	Hepes	10 mM	Thermo Fisher Scientific
	Glutamax	10 mM	Thermo Fisher Scientific
	Nicotinamide	10 mM	Sigma-Aldrich, MD, USA
	N2	1×	Thermo Fisher Scientific
	B27	1×	Thermo Fisher Scientific
	N-acetyl-L-cysteine	1 mM	Sigma-Aldrich
	Primocin	1:500	Invivogen
	Noggin	0.1 μg/mL	Peprotech, NJ, USA
	Gastrin	1 μg/mL	Tocris, Bristol, UK
	Y-27632	10 μM	Tocris
	RSPO1	1 μg/mL	Sinobiological, Beijin, China
	EGF	50 ng/mL	Peprotech
	PGE2	0.02 μM	Sigma-Aldrich
	LY-2157299	1 μM	Axon-Medchem, Groningen, The Netherlands
	SB-202190	10 μM	Sigma-Aldrich
Lysis buffer	TrisHCL pH 8.0	50 mM	Sigma-Aldrich
	EDTA pH 8.0	100 mM	Sigma-Aldrich
	NaCl	100 mM	Sigma-Aldrich
	SDS	1%	Sigma-Aldrich
	Proteinase K	20 mg/mL	Merck-Millipore, MA, USA

Table showing the reagents used in organoid processing.

## Supplementary Materials

The following are available online at https://www.mdpi.com/2072-6694/12/8/2302/s1: Table S1: List of genes regulated by calcitriol (*q* < 0.05) in normal (colon and rectum) and rectal tumor organoids identified by RNA-seq analysis, Table S2: Calcitriol RNA-seq data (Log_2_FC and *q*-value) of genes differentially expressed between colon and rectum organoids.
