# Supplementary material for: Comparative Study of Organoids from Patient-Derived Normal and Tumor Colon and Rectal Tissue
Source: Cancers (Basel). 2020 Aug 15;12(8):2302. doi: 10.3390/cancers12082302 (PMC7465167; doi:10.3390/cancers12082302)
Supplement: Supplementary file 1 [file cancers-12-02302-s001.zip › Table S2_Costales-Carrera et al.docx]

|  | COLON | | RECTUM | | RECTAL TUMOR | |
| --- | --- | --- | --- | --- | --- | --- |
| Gene | Log_2_FC | *q*-value | Log_2_FC | *q*-value | Log_2_FC | *q*-value |
| *FN1* | 2.6 | 7.9E-38 | 2.4 | 8.9E-20 | 1.2 | 4.5E-08 |
| *EEF1A2* | -0.5 | 1.0E-01 | -0.5 | 1.5E-03 | -0.4 | 2.3E-01 |
| *PDE10A* | -0.6 | 8.0E-03 | -0.6 | 3.3E-01 | 13.3 | 8.6E-01 |
| *TGFB1* | 0.1 | 8.3E-01 | -0.2 | 2.5E-01 | -0.4 | 8.5E-03 |

**Table S2: Calcitriol RNA-seq data (Log_2_FC and *q*-value) of genes differentially expressed between colon and rectum organoids**

Red, significantly upregulated

Blue, significantly downregulated
