# Supplementary material for: Comparative Study of Organoids from Patient-Derived Normal and Tumor Colon and Rectal Tissue
Source: Cancers (Basel). 2020 Aug 15;12(8):2302. doi: 10.3390/cancers12082302 (PMC7465167; doi:10.3390/cancers12082302)
Supplement: Supplementary file 1 [file cancers-12-02302-s001.zip › Table S1_Costales-Carrera et al.docx]

**Table S1: List of genes regulated by calcitriol (*q* < 0.05) in normal (colon and rectum) and rectal tumor organoids identified by RNA-seq analysis**

**Genes regulated by calcitriol in normal RECTUM organoids**

| \| *A1CF* \| \| \| --- \| --- \| \| *AASS* \| \| \| *ABCB1* \| \| \| *ABCB4* \| \| \| *ABCC2* \| \| \| *ABCD3* \| \| \| *ABHD3* \| \| \| *ACAA2* \| \| \| *ACADM* \| \| \| *ACTR3C* \| \| \| *ADAM22* \| \| \| *ADAMTS15* \| \| \| *ADAMTS9* \| \| \| *ADD3* \| \| \| *ADGRL2* \| \| \| *ADGRL3* \| \| \| *ADHFE1* \| \| \| *ADO* \| \| \| *ADRA1B* \| \| \| *AFF4* \| \| \| *AHR* \| \| \| *AHRR* \| \| \| *AIG1* \| \| \| *AKAP11* \| \| \| *AKAP12* \| \| \| *AKIRIN1* \| \| \| *AKR1C3* \| \| \| *ALOX5* \| \| \| *ALPI* \| \| \| *ALS2* \| \| \| *AMD1* \| \| \| *AMOTL1* \| \| \| *AMZ1* \| \| \| *ANKRD10* \| \| \| *ANKS4B* \| \| \| *ANXA4* \| \| \| *ANXA5* \| \| \| *AOC1* \| \| \| *APBB1* \| \| \| *APLF* \| \| \| *APPL2* \| \| \| *ARAP2* \| \| \| *ARHGAP25* \| \| \| *ARHGAP5* \| \| \| *ARHGEF12* \| \| \| *ARHGEF28* \| \| \| *ARHGEF38* \| \| \| *ARID2* \| \| \| *ARID3B* \| \| \| *ARL2BP* \| \| \| *ARL5B* \| \| \| *ARNT2* \| \| \| *ARNTL2* \| \| \| *ARPC2* \| \| \| *ASPH* \| \| \| *ATE1* \| \| \| *ATL2* \| \| \| *ATP10D* \| \| \| *ATP2B1* \| \| \| *ATP2B1-AS1* \| \| \| *ATP8A1* \| \| \| *ATRX* \| \| \| *B3GALNT1* \| \| \| *B4GALNT2* \| \| \| *BARX2* \| \| \| *BAZ2B* \| \| \| *BBS10* \| \| \| *BBX* \| \| \| *BCAS1* \| \| \| *BDP1* \| \| \| *BEND7* \| \| \| *BIRC2* \| \| \| *BIRC3* \| \| \| *BLNK* \| \| \| *BLVRA* \| \| \| *BMPR1B* \| \| \| *BTBD3* \| \| \| *C1orf131* \| \| \| *C1QTNF1* \| \| \| *C2orf54* \| \| \| *C2orf88* \| \| \| *C3* \| \| \| *C3orf52* \| \| \| *C5orf24* \| \| \| *C6orf15* \| \| \| *C9orf72* \| \| \| *CA2* \| \| \| *CAB39* \| \| \| *CACNA2D3* \| \| \| *CADPS* \| \| \| *CALB2* \| \| \| *CALM1* \| \| \| *CAMK2D* \| \| \| *CAPN12* \| \| \| *CAPN7* \| \| \| *CAPNS1* \| \| \| *CARMIL1* \| \| \| *CASP1* \| \| \| *CAV2* \| \| \| *CAVIN1* \| \| \| *CCL2* \| \| \| *CD14* \| \| \| *CD274* \| \| \| *CDA* \| \| \| *CDC42SE2* \| \| \| *CDH1* \| \| \| *CDH17* \| \| \| *CDK17* \| \| \| *CDK19* \| \| \| *CDKN2AIP* \| \| \| *CDKN2B* \| \| \| *CDON* \| \| \| *CDS1* \| \| \| *CDX2* \| \| \| *CEACAM6* \| \| \| *CEBPD* \| \| \| *CEP162* \| \| \| *CEP170* \| \| \| *CFAP97* \| \| \| *CFLAR* \| \| \| *CHD9* \| \| \| *CHDH* \| \| \| *CHIC1* \| \| \| *CHML* \| \| \| *CHRNA7* \| \| \| *CHST11* \| \| \| *CLASP1* \| \| \| *CLCF1* \| \| \| *CLCN5* \| \| \| *CLDN11* \| \| \| *CLIP4* \| \| \| *CMTR2* \| \| \| *CNTN1* \| \| \| *CNTN3* \| \| \| *COL12A1* \| \| \| *COL16A1* \| \| \| *COL18A1* \| \| \| *COLEC12* \| \| \| *CP* \| \| \| *CPA6* \| \| \| *CPE* \| \| \| *CPVL* \| \| \| *CRLF1* \| \| \| *CROT* \| \| \| *CRYBG2* \| \| \| *CSF1* \| \| \| *CSNK1G1* \| \| \| *CST6* \| \| \| *CTNNB1* \| \| \| *CTSO* \| \| \| *CTSS* \| \| \| *CUEDC1* \| \| \| *CXCL6* \| \| \| *CYB5A* \| \| \| *CYB5R4* \| \| \| *CYP19A1* \| \| \| *CYP1B1* \| \| \| *CYP24A1* \| \| \| *CYP27A1* \| \| \| *CYP2B6* \| \| \| *CYP2B7P* \| \| \| *CYP3A4* \| \| \| *CYP3A5* \| \| \| *CYP3A7* \| \| \| *CYP4F3* \| \| \| *CYTH4* \| \| \| *DAB2* \| \| \| *DACT1* \| \| \| *DAPK2* \| \| \| *DCBLD2* \| \| \| *DENND1B* \| \| \| *DENND6B* \| \| \| *DEPDC1B* \| \| \| *DGAT2* \| \| \| *DHRS9* \| \| \| *DMD* \| \| \| *DMXL2* \| \| \| *DNASE1* \| \| \| *DNER* \| \| \| *DOCK1* \| \| \| *DOCK5* \| \| \| *DOPEY1* \| \| \| *DPP4* \| \| \| *DPY19L1* \| \| \| *DPYD* \| \| \| *DTNA* \| \| \| *DTNB* \| \| \| *DUSP10* \| \| \| *DYNC2H1* \| \| \| *DYSF* \| \| \| *DZIP3* \| \| \| *EDN2* \| \| \| *EEA1* \| \| \| *EFL1* \| \| \| *EFR3A* \| \| \| *EGR2* \| \| \| *EHBP1* \| \| \| *EID1* \| \| \| *ELF1* \| \| \| *ELMOD2* \| \| \| *ELOVL7* \| \| \| *EMB* \| \| \| *ENAH* \| \| \| *ENPEP* \| \| \| *ENTPD7* \| \| \| *EPHA10* \| \| \| *EPHA4* \| \| \| *EPM2AIP1* \| \| \| *EPS8* \| \| \| *EREG* \| \| \| *ERMP1* \| \| \| *EXTL3* \| \| \| *FABP6* \| \| \| *FADS3* \| \| \| *FAM131B* \| \| \| *FAM155A* \| \| \| *FAM199X* \| \| \| *FAM20A* \| \| \| *FAM217B* \| \| \| *FAM3C* \| \| \| *FAM43A* \| \| \| *FAM84A* \| \| \| *FAM86DP* \| \| \| *FAM91A1* \| \| \| *FBLIM1* \| \| \| *FEM1B* \| \| \| *FER1L6* \| \| \| *FLG* \| \| \| *FLG-AS1* \| \| \| *FLRT3* \| \| \| *FN1* \| \| \| *FNBP1L* \| \| \| *FNDC3A* \| \| \| *FNIP2* \| \| \| *FOCAD* \| \| \| *FOS* \| \| \| *FRK* \| \| \| *FSIP2* \| \| \| *FTH1* \| \| \| *FTH1P15* \| \| \| *FTH1P7* \| \| \| *FTH1P8* \| \| \| *FYN* \| \| \| *G6PD* \| \| \| *GABRE* \| \| \| *GADD45A* \| \| \| *GALNT5* \| \| \| *GDE1* \| \| \| *GDF11* \| \| \| *GDPD3* \| \| \| *GEM* \| \| \| *GGT1* \| \| \| *GIMAP2* \| \| \| *GIPC2* \| \| \| *GLB1L2* \| \| \| *GNE* \| \| \| *GNPAT* \| \| \| *GOLIM4* \| \| \| *GPN1* \| \| \| *GPR160* \| \| \| *GPR37* \| \| \| *GRAMD4* \| \| \| *GRHL1* \| \| \| *GRIP2* \| \| \| *GRK5* \| \| \| *GSTA1* \| \| \| *GULP1* \| \| \| *HCN1* \| \| \| *HCP5* \| \| \| *HIF1A* \| \| \| *HIPK2* \| \| \| *HIPK3* \| \| \| *HIVEP2* \| \| \| *HMGA2* \| \| \| *HNF4G* \| \| \| *HNRNPA1P26* \| \| \| *HOOK1* \| \| \| *HPS3* \| \| \| *HRCT1* \| \| \| *HRNR* \| \| \| *HSD17B2* \| \| \| *HSD3B1* \| \| \| *HSPA12A* \| \| \| *ICAM1* \| \| \| *IFI16* \| \| \| *IFITM10* \| \| \| *IFNE* \| \| \| *IFT74* \| \| \| *IGFBP6* \| \| \| *IGFL2-AS1* \| \| \| *IGFN1* \| \| \| *IL17RB* \| \| \| *IL1RAPL1* \| \| \| *IL6R* \| \| \| *IL6ST* \| \| \| *INPP1* \| \| \| *INPP4B* \| \| \| *INTS6L* \| \| \| *IQCB1* \| \| \| *IQUB* \| \| \| *IRF8* \| \| \| *ISM1* \| \| \| *ITGA2* \| \| \| *ITGB8* \| \| \| *ITM2B* \| \| \| *JCAD* \| \| \| *JKAMP* \| \| \| *JMJD1C* \| \| \| *JUNB* \| \| \| *KCNMB4* \| \| \| *KCTD1* \| \| \| *KDM1A* \| \| \| *KIAA1211* \| \| \| *KIAA1324L* \| \| \| *KIAA1551* \| \| \| *KIDINS220* \| \| \| *KIF21A* \| \| \| *KIF3C* \| \| \| *KLHL23* \| \| \| *KLHL5* \| \| \| *KLK13* \| \| \| *KLK5* \| \| \| *KLK6* \| \| \| *KLK7* \| \| \| *KRT16* \| \| \| *KRT6B* \| \| \| *KSR2* \| \| \| *KYNU* \| \| \| *LACC1* \| \| \| *LAD1* \| \| \| *LAMA2* \| \| \| *LAP3* \| \| \| *LBH* \| \| \| *LCA5* \| \| \| *LDLRAP1* \| \| \| *LGR5* \| \| \| *LINC00649* \| \| \| *LINC01559* \| \| \| *LINC01801* \| \| \| *LINC02474* \| \| \| *LINC02588* \| \| \| *LMO3* \| \| \| *LPAR6* \| \| \| *LPCAT2* \| \| \| *LRBA* \| \| \| *LRIG1* \| \| \| *LRRC61* \| \| \| *LRRC75B* \| \| \| *LRRN4* \| \| \| *LTB* \| \| \| *LTN1* \| \| \| *LXN* \| \| \| *LYPD5* \| \| \| *LZTS2* \| \| \| *MACC1* \| \| \| *MALL* \| \| \| *MAOB* \| \| \| *MAP3K12* \| \| \| *MAP3K21* \| \| \| *MAP4K4* \| \| \| *MAP7* \| \| \| *MAP9* \| \| \| *MATR3* \| \| \| *MEF2C* \| \| \| *MELTF* \| \| \| *MELTF-AS1* \| \| \| *MERTK* \| \| \| *MFSD1* \| \| \| *MFSD2A* \| \| \| *MFSD6* \| \| \| *MGAT4A* \| \| \| *MGST1* \| \| \| *MIB1* \| \| \| *MICAL3* \| \| \| *MLLT3* \| \| \| *MMD* \| \| \| *MMP10* \| \| \| *MMP24* \| \| \| *MMP7* \| \| \| *MOGAT1* \| \| \| *MORC4* \| \| \| *MOSMO* \| \| \| *MPHOSPH10* \| \| \| *MPHOSPH6* \| \| \| *MPP5* \| \| \| *MSI1* \| \| \| *MTUS1* \| \| \| *MYO1B* \| \| \| *MYO6* \| \| \| *MYOM1* \| \| \| *NAB1* \| \| \| *NAPEPLD* \| \| \| *NAV3* \| \| \| *NBEA* \| \| \| *NBPF1* \| \| \| *NBPF3* \| \| \| *NDRG4* \| \| \| *NEK1* \| \| \| *NET1* \| \| \| *NEURL3* \| \| \| *NFKBIA* \| \| \| *NHS* \| \| \| *NIPAL2* \| \| \| *NLRC5* \| \| \| *NNT* \| \| \| *NOX1* \| \| \| *NPNT* \| \| \| *NPSR1* \| \| \| *NR5A2* \| \| \| *NRCAM* \| \| \| *NRIP1* \| \| \| *NRP2* \| \| \| *NSUN6* \| \| \| *ODF2L* \| \| \| *OGFRL1* \| \| \| *ONECUT2* \| \| \| *OPHN1* \| \| \| *OSGIN2* \| \| \| *OXTR* \| \| \| *P2RY1* \| \| \| *PADI1* \| \| \| *PAPSS1* \| \| \| *PARM1* \| \| \| *PARP12* \| \| \| *PARP14* \| \| \| *PBX1* \| \| \| *PCNX4* \| \| \| *PDCD4* \| \| \| *PDCD6* \| \| \| *PDCD6IP* \| \| \| *PDE3B* \| \| \| *PDE4B* \| \| \| *PDGFA* \| \| \| *PDLIM5* \| \| \| *PDZD8* \| \| \| *PEX1* \| \| \| *PEX13* \| \| \| *PFN2* \| \| \| *PGM2* \| \| \| *PGM2L1* \| \| \| *PHACTR3* \| \| \| *PHF10* \| \| \| *PHIP* \| \| \| *PHKA2* \| \| \| *PIEZO2* \| \| \| *PIK3CA* \| \| \| *PKD2* \| \| \| *PKIB* \| \| \| *PKN2* \| \| \| *PLB1* \| \| \| *PLEKHA5* \| \| \| *PLEKHA6* \| \| \| *PLEKHA7* \| \| \| *PLK2* \| \| \| *PLLP* \| \| \| *PLPP3* \| \| \| *PLS1* \| \| \| *PLS3* \| \| \| *PLXDC2* \| \| \| *PMEPA1* \| \| \| *PNPLA8* \| \| \| *POLK* \| \| \| *PPIP5K2* \| \| \| *PPP1R1C* \| \| \| *PPP1R21* \| \| \| *PPP1R9A* \| \| \| *PPP4R2* \| \| \| *PREPL* \| \| \| *PRKACB* \| \| \| *PRKAR1A* \| \| \| *PRKAR1B* \| \| \| *PRKCG* \| \| \| *PRKD1* \| \| \| *PRKG2* \| \| \| *PRMT9* \| \| \| *PRODH* \| \| \| *PROS1* \| \| \| *PRR13* \| \| \| *PRR5L* \| \| \| *PRRG1* \| \| \| *PRSS22* \| \| \| *PRSS33* \| \| \| *PTAFR* \| \| \| *PTAR1* \| \| \| *PTBP3* \| \| \| *PTGDS* \| \| \| *PTGS2* \| \| \| *PTPRK* \| \| \| *PYCARD* \| \| \| *RAB11FIP1* \| \| \| *RAB11FIP2* \| \| \| *RAB29* \| \| \| *RAB37* \| \| \| *RAB3GAP2* \| \| \| *RABEP1* \| \| \| *RABGAP1L* \| \| \| *RAI2* \| \| \| *RALGAPB* \| \| \| *RALGPS2* \| \| \| *RAMP1* \| \| \| *RANBP2* \| \| \| *RANBP6* \| \| \| *RARRES1* \| \| \| *RARRES2* \| \| \| *RASAL2* \| \| \| *RASL11A* \| \| \| *RASSF10* \| \| \| *RB1* \| \| \| *RBM41* \| \| \| *RBPMS* \| \| \| *RC3H2* \| \| \| *RCAN2* \| \| \| *RCBTB2* \| \| \| *RDX* \| \| \| *REPIN1* \| \| \| *REV3L* \| \| \| *RICTOR* \| \| \| *RIMS3* \| \| \| *RIN3* \| \| \| *RIPK2* \| \| \| *RMND5A* \| \| \| *RNF103* \| \| \| *RNF128* \| \| \| *RNF19A* \| \| \| *RNF224* \| \| \| *RNF5* \| \| \| *ROBO1* \| \| \| *RPGRIP1L* \| \| \| *RPL32P3* \| \| \| *RPS6KA3* \| \| \| *RPTN* \| \| \| *RRAGB* \| \| \| *RRM2B* \| \| \| *RSPRY1* \| \| \| *RUNDC3B* \| \| \| *SAA2* \| \| \| *SAMD12* \| \| \| *SAMD9* \| \| \| *SARDH* \| \| \| *SATB1* \| \| \| *SATB1-AS1* \| \| \| *SAXO2* \| \| \| *SCML1* \| \| \| *SCN8A* \| \| \| *SCOC* \| \| \| *SCYL2* \| \| \| *SEC14L1* \| \| \| *SEC14L6* \| \| \| *SECTM1* \| \| \| *SELENOP* \| \| \| *SEMA3B* \| \| \| *SEMA3C* \| \| \| *SEMA6A* \| \| \| *SERINC2* \| \| \| *SERPINA1* \| \| \| *SERPINB1* \| \| \| *SESN3* \| \| \| *SETX* \| \| \| *SGMS2* \| \| \| *SGPP2* \| \| \| *SH3BGRL* \| \| \| *SH3BGRL2* \| \| \| *SH3BP4* \| \| \| *SH3PXD2B* \| \| \| *SH3TC1* \| \| \| *SHH* \| \| \| *SHTN1* \| \| \| *SIK1* \| \| \| *SIRPA* \| \| \| *SIX4* \| \| \| *SKIL* \| \| \| *SLC15A2* \| \| \| *SLC1A1* \| \| \| *SLC25A4* \| \| \| *SLC28A3* \| \| \| *SLC30A10* \| \| \| *SLC34A2* \| \| \| *SLC34A3* \| \| \| *SLC37A2* \| \| \| *SLC44A5* \| \| \| *SLC46A3* \| \| \| *SLC4A7* \| \| \| *SLC51B* \| \| \| *SLC6A20* \| \| \| *SLC9A1* \| \| \| *SLCO4A1* \| \| \| *SLCO4A1-AS1* \| \| \| *SLFN5* \| \| \| *SMARCA1* \| \| \| *SMARCA5* \| \| \| *SMOC2* \| \| \| *SNHG14* \| \| \| *SNHG20* \| \| \| *SNN* \| \| \| *SNRK* \| \| \| *SNX30* \| \| \| *SOCS4* \| \| \| *SOX4* \| \| \| *SPATA2L* \| \| \| *SPATS2L* \| \| \| *SPOCD1* \| \| \| *SPTBN5* \| \| \| *SPTSSB* \| \| \| *SRI* \| \| \| *SSX2IP* \| \| \| *STARD8* \| \| \| *STAT1* \| \| \| *STEAP3* \| \| \| *STEAP4* \| \| \| *STK39* \| \| \| *STRADB* \| \| \| *STXBP3* \| \| \| *STXBP4* \| \| \| *SULF1* \| \| \| *SULT1C2* \| \| \| *SULT1C2P1* \| \| \| *SVIL* \| \| \| *SYNPR* \| \| \| *SYT12* \| \| \| *SYT8* \| \| \| *SYTL2* \| \| \| *TAF9B* \| \| \| *TAOK1* \| \| \| *TBC1D1* \| \| \| *TBC1D9* \| \| \| *TBCK* \| \| \| *TBL1XR1* \| \| \| *TCAF1* \| \| \| *TCAF1P1* \| \| \| *TCF12* \| \| \| *TCTN1* \| \| \| *TENT5A* \| \| \| *TEX15* \| \| \| *TEX9* \| \| \| *TFAP2A* \| \| \| *TFPI* \| \| \| *TFPI2* \| \| \| *TFRC* \| \| \| *THBD* \| \| \| *THRB* \| \| \| *THSD7A* \| \| \| *TIA1* \| \| \| *TIMP2* \| \| \| *TIPARP* \| \| \| *TLR4* \| \| \| *TMEM106B* \| \| \| *TMEM131* \| \| \| *TMEM132A* \| \| \| *TMEM176B* \| \| \| *TMEM200A* \| \| \| *TMEM263* \| \| \| *TMEM37* \| \| \| *TMEM63A* \| \| \| *TMEM87B* \| \| \| *TMEM98* \| \| \| *TMPRSS2* \| \| \| *TMTC4* \| \| \| *TNC* \| \| \| *TNF* \| \| \| *TNFAIP2* \| \| \| *TNFAIP3* \| \| \| *TNFRSF11A* \| \| \| *TNNI1* \| \| \| *TNPO1* \| \| \| *TNS3* \| \| \| *TOMM34* \| \| \| *TOX* \| \| \| *TP53BP1* \| \| \| *TPST1* \| \| \| *TRAK2* \| \| \| *TRIB1* \| \| \| *TRIM23* \| \| \| *TRIM35* \| \| \| *TRIM38* \| \| \| *TRIM56* \| \| \| *TRIM6* \| \| \| *TRIO* \| \| \| *TRIP11* \| \| \| *TRPV6* \| \| \| *TSKU* \| \| \| *TSPYL4* \| \| \| *TTC3P1* \| \| \| *TUBA1A* \| \| \| *TUG1* \| \| \| *TUSC3* \| \| \| *TWF1* \| \| \| *TXNRD1* \| \| \| *TYMP* \| \| \| *UBA3* \| \| \| *UBE2D1* \| \| \| *UBE2E2* \| \| \| *UBLCP1* \| \| \| *UBR1* \| \| \| *UCA1* \| \| \| *UCKL1* \| \| \| *UGDH* \| \| \| *UGT2B17* \| \| \| *USP12* \| \| \| *USP45* \| \| \| *USP53* \| \| \| *VAV1* \| \| \| *VEZF1* \| \| \| *VGLL3* \| \| \| *VPS13A* \| \| \| *VPS13B* \| \| \| *VPS50* \| \| \| *VWA3B* \| \| \| *VWDE* \| \| \| *WASHC5* \| \| \| *WDR55* \| \| \| *WDR72* \| \| \| *WDR78* \| \| \| *WNK1* \| \| \| *XKRX* \| \| \| *YAP1* \| \| \| *YBX2* \| \| \| *ZBED6CL* \| \| \| *ZBTB38* \| \| \| *ZBTB41* \| \| \| *ZC3H12C* \| \| \| *ZCCHC17* \| \| \| *ZDHHC21* \| \| \| *ZFYVE16* \| \| \| *ZMYM4* \| \| \| *ZNF12* \| \| \| *ZNF253* \| \| \| *ZNF260* \| \| \| *ZNF30* \| \| \| *ZNF441* \| \| \| *ZNF512B* \| \| \| *ZNF528* \| \| \| *ZNF594* \| \| \| *ZNF608* \| \| \| *ZNF664* \| \| \| *ZNF774* \| \| \| *ZNF780A* \| \| \| *ZNF827* \| \| \| *ZNF91* \| \| \| *A4GALT* \| \| *ABCA7* \| \| *ABCG1* \| \| *ABHD11* \| \| *ABHD17C* \| \| *ABLIM3* \| \| *ACER2* \| \| *ACHE* \| \| *ACP5* \| \| *ACSF2* \| \| *ACSL5* \| \| *ACSS1* \| \| *ACSS2* \| \| *ADA* \| \| *ADAM19* \| \| *ADAM8* \| \| *ADAMTSL4* \| \| *ADCK2* \| \| *ADCY3* \| \| *ADGRF1* \| \| *ADGRG6* \| \| *ADM* \| \| *ADM2* \| \| *ADORA2B* \| \| *ADRA2A* \| \| *ADRB1* \| \| *ADRB2* \| \| *AGR2* \| \| *AGR3* \| \| *AHCYL2* \| \| *AIF1L* \| \| *AIFM3* \| \| *AK4* \| \| *AKR1A1* \| \| *AKR7A2* \| \| *ALAS1* \| \| *ALDH1L1* \| \| *ALDH1L2* \| \| *ALDH3A1* \| \| *ALDH3B1* \| \| *ALDOA* \| \| *ALDOC* \| \| *ALPG* \| \| *ANGPTL4* \| \| *ANKEF1* \| \| *ANKRD1* \| \| *ANKRD9* \| \| *ANP32B* \| \| *ANXA1* \| \| *ANXA10* \| \| *ANXA3* \| \| *APCDD1* \| \| *APOBR* \| \| *APOL6* \| \| *AQP1* \| \| *AQP3* \| \| *AQP5* \| \| *AQP7* \| \| *ARF6* \| \| *ARHGAP27* \| \| *ARHGAP45* \| \| *ARHGAP6* \| \| *ARHGEF19* \| \| *ARHGEF2* \| \| *ARHGEF40* \| \| *ARRB1* \| \| *ARRDC2* \| \| *ASIC1* \| \| *ASPHD1* \| \| *ASPHD2* \| \| *ASS1* \| \| *ATF4* \| \| *ATP13A2* \| \| *ATP1A1* \| \| *ATP1B1* \| \| *ATP2A3* \| \| *ATP2C2* \| \| *AURKB* \| \| *AXL* \| \| *AZGP1* \| \| *B3GALT5* \| \| *B3GNT7* \| \| *B4GAT1* \| \| *BACE2* \| \| *BAG1* \| \| *BAIAP2L2* \| \| *BBC3* \| \| *BCAR3* \| \| *BCAT2* \| \| *BCL2L14* \| \| *BHLHA15* \| \| *BHLHE41* \| \| *BICDL1* \| \| *BIK* \| \| *BLCAP* \| \| *BLVRB* \| \| *BMP7* \| \| *C10orf99* \| \| *C11orf24* \| \| *C16orf74* \| \| *C1orf116* \| \| *C2CD2L* \| \| *C2CD4A* \| \| *C2orf72* \| \| *C5orf46* \| \| *C6orf223* \| \| *C9orf152* \| \| *CABLES1* \| \| *CACNA1C* \| \| *CADM4* \| \| *CALU* \| \| *CAPN2* \| \| *CAPN5* \| \| *CAPN8* \| \| *CARS* \| \| *CAST* \| \| *CBLC* \| \| *CBS* \| \| *CCDC3* \| \| *CCK* \| \| *CCL20* \| \| *CCL24* \| \| *CCL28* \| \| *CCND2* \| \| *CCRL2* \| \| *CD109* \| \| *CD22* \| \| *CD276* \| \| *CD320* \| \| *CD44* \| \| *CD55* \| \| *CD96* \| \| *CDC25B* \| \| *CDC42EP1* \| \| *CDC42EP2* \| \| *CDC42EP4* \| \| *CDC42EP5* \| \| *CDCA7* \| \| *CDH23* \| \| *CDK2AP2* \| \| *CDKN1C* \| \| *CDKN2D* \| \| *CDT1* \| \| *CDX1* \| \| *CEACAM1* \| \| *CEACAM5* \| \| *CEMIP* \| \| *CEMIP2* \| \| *CES2* \| \| *CFD* \| \| *CHAC1* \| \| *CHCHD10* \| \| *CHKA* \| \| *CHPF* \| \| *CIT* \| \| *CKAP4* \| \| *CKB* \| \| *CKS1B* \| \| *CLCA4* \| \| *CLDN3* \| \| *CLDN4* \| \| *CLIC3* \| \| *CLIC5* \| \| *CLSTN1* \| \| *CMBL* \| \| *CMIP* \| \| *CNN2* \| \| *CNPPD1* \| \| *COL13A1* \| \| *COL17A1* \| \| *COL4A1* \| \| *COL4A2* \| \| *COL5A3* \| \| *COL6A1* \| \| *COLGALT1* \| \| *COQ8A* \| \| *COX6B2* \| \| *CPAMD8* \| \| *CPLX1* \| \| *CPT1A* \| \| *CRACR2A* \| \| *CRAT* \| \| *CREB3L1* \| \| *CREG2* \| \| *CRIP1* \| \| *CRIP2* \| \| *CSPG5* \| \| *CSTB* \| \| *CTH* \| \| *CTSE* \| \| *CTSV* \| \| *CX3CL1* \| \| *CXXC5* \| \| *CYBRD1* \| \| *CYR61* \| \| *CYSTM1* \| \| *DCPS* \| \| *DDB2* \| \| *DDX11* \| \| *DEAF1* \| \| *DEGS2* \| \| *DENND1A* \| \| *DENND1C* \| \| *DERL3* \| \| *DESI2* \| \| *DGKB* \| \| *DGKD* \| \| *DHCR7* \| \| *DHRS3* \| \| *DIO3OS* \| \| *DNAH2* \| \| *DNAJB9* \| \| *DNAJC12* \| \| *DNASE2* \| \| *DNM2* \| \| *DRAM1* \| \| *DTX1* \| \| *DUOX2* \| \| *DUOXA2* \| \| *DUSP4* \| \| *E2F1* \| \| *E2F2* \| \| *EBP* \| \| *ECI1* \| \| *ECM1* \| \| *EEF1A2* \| \| *EFHD2* \| \| *EFNA3* \| \| *EFNB1* \| \| *EGFL7* \| \| *EGLN3* \| \| *EHD2* \| \| *EIF4EBP1* \| \| *EML2* \| \| *EMP1* \| \| *EMP2* \| \| *EMP3* \| \| *ENO2* \| \| *ENTPD6* \| \| *EPB41L1* \| \| *EPDR1* \| \| *EPHB3* \| \| *EPN3* \| \| *EPS8L1* \| \| *EPS8L3* \| \| *ERN2* \| \| *ESAM* \| \| *ETHE1* \| \| *ETV4* \| \| *EXPH5* \| \| *FA2H* \| \| *FADS2* \| \| *FADS6* \| \| *FAIM2* \| \| *FAM114A1* \| \| *FAM171A1* \| \| *FAM222A* \| \| *FAM3D* \| \| *FASN* \| \| *FAT1* \| \| *FBLN2* \| \| *FBXL16* \| \| *FBXO2* \| \| *FBXO32* \| \| *FCMR* \| \| *FDFT1* \| \| *FDPS* \| \| *FFAR4* \| \| *FGFBP1* \| \| *FGFR2* \| \| *FGFRL1* \| \| *FIBCD1* \| \| *FICD* \| \| *FLNB* \| \| *FLNC* \| \| *FMOD* \| \| *FOXA2* \| \| *FOXA3* \| \| *FOXO4* \| \| *FOXQ1* \| \| *FRMD3* \| \| *FRMPD1* \| \| *FSCN1* \| \| *FUT1* \| \| *FUT2* \| \| *FUT3* \| \| *FXYD3* \| \| *FXYD5* \| \| *GAL3ST4* \| \| *GALE* \| \| *GALNT12* \| \| *GARS* \| \| *GAS7* \| \| *GCAT* \| \| *GDPD2* \| \| *GDPD5* \| \| *GFPT1* \| \| *GGT6* \| \| *GLG1* \| \| *GLYCTK* \| \| *GMDS* \| \| *GMPPA* \| \| *GNAZ* \| \| *GOT1* \| \| *GPI* \| \| *GPRC5A* \| \| *GPRC5C* \| \| *GPX2* \| \| *GPX4* \| \| *GRB10* \| \| *GREM1* \| \| *GRHL3* \| \| *GSTM2* \| \| *GSTM3* \| \| *GSTM4* \| \| *GSTP1* \| \| *H1F0* \| \| *H1FX* \| \| *H2AFJ* \| \| *H2AFZ* \| \| *HDAC11* \| \| *HERPUD1* \| \| *HES2* \| \| *HES6* \| \| *HGSNAT* \| \| *HID1* \| \| *HLA-DMA* \| \| *HLA-DMB* \| \| *HMGCS2* \| \| *HMOX1* \| \| *HOXB8* \| \| *HPCAL1* \| \| *HPDL* \| \| *HRASLS5* \| \| *HS3ST2* \| \| *HSPA2* \| \| *HYAL1* \| \| *ICAM2* \| \| *IDH2* \| \| *IDI1* \| \| *IER3* \| \| *IFI35* \| \| *IFI6* \| \| *IGFBP4* \| \| *IGFL4* \| \| *IGSF9* \| \| *IL10RA* \| \| *IL17RD* \| \| *IL1RN* \| \| *IL22RA1* \| \| *IL2RG* \| \| *ILVBL* \| \| *IMPA2* \| \| *IMPDH1* \| \| *INO80C* \| \| *IQGAP2* \| \| *IRS2* \| \| *ISX* \| \| *ITGA5* \| \| *ITGB2-AS1* \| \| *ITGB4* \| \| *ITGB5* \| \| *ITGB7* \| \| *ITPK1* \| \| *JAG1* \| \| *KALRN* \| \| *KAT2B* \| \| *KCNE3* \| \| *KCNG1* \| \| *KCNJ12* \| \| *KCNJ4* \| \| *KCNN4* \| \| *KCTD14* \| \| *KDELC2* \| \| *KDELR2* \| \| *KDELR3* \| \| *KDM7A-DT* \| \| *KHK* \| \| *KIF12* \| \| *KIF2C* \| \| *KIFC3* \| \| *KLF2* \| \| *KLF4* \| \| *KLHDC7A* \| \| *KLHL29* \| \| *KLK1* \| \| *KRT17* \| \| *KRT20* \| \| *LAMA4* \| \| *LAMB2* \| \| *LARS* \| \| *LCK* \| \| *LCN2* \| \| *LDHA* \| \| *LDHD* \| \| *LDLR* \| \| *LGALS1* \| \| *LGALS3* \| \| *LGMN* \| \| *LINC00239* \| \| *LINC00261* \| \| *LINC00482* \| \| *LINC00520* \| \| *LINC00668* \| \| *LINC01133* \| \| *LINC02320* \| \| *LIPG* \| \| *LLGL2* \| \| *LMAN2* \| \| *LONP1* \| \| *LOXL1* \| \| *LOXL1-AS1* \| \| *LOXL4* \| \| *LPAR2* \| \| *LPCAT4* \| \| *LRP1* \| \| *LRP11* \| \| *LRP4* \| \| *LSS* \| \| *LTBP3* \| \| *LTBP4* \| \| *LY6D* \| \| *LY6E* \| \| *LY6G6C* \| \| *LYPD6B* \| \| *LYZ* \| \| *MAFF* \| \| *MAGED1* \| \| *MANSC1* \| \| *MAP2K2* \| \| *MAP2K3* \| \| *MAPRE3* \| \| *MARS* \| \| *MARVELD1* \| \| *MBOAT1* \| \| *MCM2* \| \| *MDFI* \| \| *MDH2* \| \| *ME1* \| \| *MEGF6* \| \| *MESD* \| \| *METTL27* \| \| *MFSD4A* \| \| *MGAT3* \| \| *MGAT5* \| \| *MGST2* \| \| *MICA* \| \| *MIR210HG* \| \| *MKNK2* \| \| *MLEC* \| \| *MLPH* \| \| *MMAB* \| \| *MOCOS* \| \| *MPRIP* \| \| *MRPL14* \| \| *MRPL2* \| \| *MRPS18B* \| \| *MRPS6* \| \| *MSI2* \| \| *MSLN* \| \| *MSLNL* \| \| *MSN* \| \| *MT1A* \| \| *MTCL1* \| \| *MTHFD1L* \| \| *MTHFD2* \| \| *MUC1* \| \| *MUC13* \| \| *MUC2* \| \| *MUC3A* \| \| *MUC4* \| \| *MUC5AC* \| \| *MVB12B* \| \| *MVD* \| \| *MYDGF* \| \| *MYEOV* \| \| *MYO15B* \| \| *MYO1E* \| \| *MYORG* \| \| *N4BP3* \| \| *NAA80* \| \| *NANS* \| \| *NBL1* \| \| *NDRG2* \| \| *NEBL* \| \| *NECTIN1* \| \| *NECTIN2* \| \| *NEDD9* \| \| *NFE2L1* \| \| *NFE2L3* \| \| *NFIX* \| \| *NID2* \| \| *NKD2* \| \| *NMU* \| \| *NMUR2* \| \| *NOSTRIN* \| \| *NPY4R* \| \| *NR1D1* \| \| *NR1H3* \| \| *NR2F6* \| \| *NR3C1* \| \| *NR4A1* \| \| *NRSN2-AS1* \| \| *NT5C* \| \| *NT5DC2* \| \| *NT5M* \| \| *NTMT1* \| \| *NTN4* \| \| *NUDT8* \| \| *NUP210* \| \| *NUPR1* \| \| *NXN* \| \| *NYNRIN* \| \| *OAS1* \| \| *OAS3* \| \| *OASL* \| \| *OCLN* \| \| *ONECUT3* \| \| *OSBP2* \| \| *OSBPL10* \| \| *OSTC* \| \| *OTUD1* \| \| *OTULINL* \| \| *OVOL1* \| \| *P2RX1* \| \| *P3H2* \| \| *P4HA2* \| \| *P4HB* \| \| *PADI2* \| \| *PAG1* \| \| *PALD1* \| \| *PAQR8* \| \| *PBX4* \| \| *PC* \| \| *PCBD1* \| \| *PCK1* \| \| *PCSK6* \| \| *PCSK9* \| \| *PDE4A* \| \| *PDE4C* \| \| *PDE4D* \| \| *PDIA5* \| \| *PDIA6* \| \| *PEX11A* \| \| *PFKFB2* \| \| *PFKFB3* \| \| *PFKFB4* \| \| *PGM1* \| \| *PHF19* \| \| *PHGDH* \| \| *PHGR1* \| \| *PHLDB2* \| \| *PHYHD1* \| \| *PI3* \| \| *PIGR* \| \| *PIGZ* \| \| *PIM3* \| \| *PIR* \| \| *PITPNM3* \| \| *PLA2G2A* \| \| *PLAC8* \| \| *PLBD1* \| \| *PLCXD1* \| \| *PLEK2* \| \| *PLEKHH3* \| \| *PLEKHM1* \| \| *PLEKHM1P1* \| \| *PLEKHS1* \| \| *PLIN2* \| \| *PLOD1* \| \| *PLP2* \| \| *PLTP* \| \| *PLXNA2* \| \| *PMM1* \| \| *PMP22* \| \| *PNMA1* \| \| *POC1A* \| \| *PPARD* \| \| *PPARG* \| \| *PPFIBP2* \| \| *PPM1L* \| \| *PPP1R14D* \| \| *PPP1R1B* \| \| *PPP1R3B* \| \| *PRDM1* \| \| *PRDX1* \| \| *PRDX5* \| \| *PREP* \| \| *PRKAB1* \| \| *PRKCA* \| \| *PRKCSH* \| \| *PROM1* \| \| *PRR15* \| \| *PRR15L* \| \| *PRSS1* \| \| *PRSS12* \| \| *PRSS23* \| \| *PRSS3* \| \| *PRSS3P1* \| \| *PSAPL1* \| \| *PSAT1* \| \| *PSCA* \| \| *PTGER4* \| \| *PTPRN2* \| \| *PTPRU* \| \| *PTTG1* \| \| *PWWP2B* \| \| *PXDC1* \| \| *PYCR1* \| \| *PYGB* \| \| *QPCT* \| \| *QPCTL* \| \| *QPRT* \| \| *QSOX1* \| \| *RAB6B* \| \| *RAD23A* \| \| *RAPGEF3* \| \| *RASAL1* \| \| *RBCK1* \| \| *RDH13* \| \| *REEP1* \| \| *REEP4* \| \| *REG4* \| \| *RETSAT* \| \| *RFLNA* \| \| *RHBDL1* \| \| *RILP* \| \| *RIMKLA* \| \| *RIMS4* \| \| *RNASE1* \| \| *RNF125* \| \| *RNF144B* \| \| *RNF165* \| \| *RNF19B* \| \| *RNF223* \| \| *RNF24* \| \| *RNPEPL1* \| \| *ROR1* \| \| *RPN2* \| \| *RRBP1* \| \| *RSPH1* \| \| *RTN3* \| \| *RXFP4* \| \| *S100A10* \| \| *S100P* \| \| *SAT1* \| \| *SAV1* \| \| *SCARA3* \| \| *SCARB1* \| \| *SCD* \| \| *SCEL* \| \| *SCNN1A* \| \| *SDCBP2* \| \| *SDR16C5* \| \| *SEC13* \| \| *SEC24D* \| \| *SEC61A1* \| \| *SEC61G* \| \| *SELENOO* \| \| *SELENOS* \| \| *SEMA5A* \| \| *SFMBT2* \| \| *SFN* \| \| *SFTA1P* \| \| *SFTA2* \| \| *SH3BP1* \| \| *SHF* \| \| *SHMT2* \| \| *SIDT2* \| \| *SIGLEC15* \| \| *SIPA1L2* \| \| *SLC12A8* \| \| *SLC13A5* \| \| *SLC14A1* \| \| *SLC16A1* \| \| *SLC16A9* \| \| *SLC17A9* \| \| *SLC19A1* \| \| *SLC1A4* \| \| *SLC25A1* \| \| *SLC26A9* \| \| *SLC28A2* \| \| *SLC29A1* \| \| *SLC29A3* \| \| *SLC2A1* \| \| *SLC2A3* \| \| *SLC35C1* \| \| *SLC35E4* \| \| *SLC37A1* \| \| *SLC38A5* \| \| *SLC39A14* \| \| *SLC39A7* \| \| *SLC3A2* \| \| *SLC43A1* \| \| *SLC43A3* \| \| *SLC44A2* \| \| *SLC45A3* \| \| *SLC48A1* \| \| *SLC4A3* \| \| *SLC50A1* \| \| *SLC52A3* \| \| *SLC5A1* \| \| *SLC6A6* \| \| *SLC6A8* \| \| *SLC7A1* \| \| *SLC7A11* \| \| *SLC7A11-AS1* \| \| *SLC7A5* \| \| *SLC7A8* \| \| *SLC9A3* \| \| *SLCO2B1* \| \| *SLITRK6* \| \| *SLPI* \| \| *SMAD6* \| \| *SMAGP* \| \| *SMOC1* \| \| *SMOX* \| \| *SNCG* \| \| *SND1* \| \| *SNHG18* \| \| *SNTA1* \| \| *SOD3* \| \| *SORBS1* \| \| *SOWAHB* \| \| *SOWAHC* \| \| *SOX7* \| \| *SPAG4* \| \| *SPDEF* \| \| *SPINK4* \| \| *SPINK5* \| \| *SPIRE2* \| \| *SPOCK2* \| \| *SPON2* \| \| *SPRY1* \| \| *SRC* \| \| *SREBF1* \| \| *SRGAP3* \| \| *SRPRB* \| \| *SSBP3* \| \| *SSBP4* \| \| *SSR2* \| \| *ST3GAL4* \| \| *ST6GAL1* \| \| *ST6GALNAC6* \| \| *STARD10* \| \| *STC1* \| \| *STK40* \| \| *STN1* \| \| *STT3A* \| \| *STXBP1* \| \| *SUGCT* \| \| *SURF4* \| \| *SUSD3* \| \| *SYNE3* \| \| *SYNPO* \| \| *SYTL1* \| \| *SYTL4* \| \| *TACC1* \| \| *TAGLN2* \| \| *TBC1D8* \| \| *TBL1X* \| \| *TBL2* \| \| *TCEA3* \| \| *TCF7* \| \| *TCN1* \| \| *TCN2* \| \| *TESC* \| \| *TFCP2L1* \| \| *TFF1* \| \| *TFF2* \| \| *TFF3* \| \| *TGFBI* \| \| *TGFBR3* \| \| *THEM6* \| \| *TIMM44* \| \| *TIMP1* \| \| *TIMP3* \| \| *TIMP4* \| \| *TINCR* \| \| *TJP3* \| \| *TK1* \| \| *TKT* \| \| *TLE1* \| \| *TLN2* \| \| *TLNRD1* \| \| *TM4SF1* \| \| *TM4SF5* \| \| *TMBIM1* \| \| *TMC5* \| \| *TMC7* \| \| *TMED9* \| \| *TMEM105* \| \| *TMEM141* \| \| *TMEM173* \| \| *TMEM184A* \| \| *TMEM200B* \| \| *TMEM214* \| \| *TMEM229B* \| \| *TMEM238L* \| \| *TMEM246* \| \| *TMEM45B* \| \| *TMEM63C* \| \| *TMEM92* \| \| *TMEM94* \| \| *TMSB10* \| \| *TMTC2* \| \| *TNFRSF1B* \| \| *TNFRSF21* \| \| *TNK2* \| \| *TNNT1* \| \| *TNS4* \| \| *TOM1L2* \| \| *TOR1B* \| \| *TOX2* \| \| *TP53I13* \| \| *TPM4* \| \| *TPPP* \| \| *TPRG1* \| \| *TPSP2* \| \| *TRABD* \| \| *TRABD2A* \| \| *TRAM2* \| \| *TRBC2* \| \| *TREX1* \| \| *TRIM16* \| \| *TRIM29* \| \| *TRIM31* \| \| *TRIM47* \| \| *TRIM7* \| \| *TSC22D1* \| \| *TSPAN11* \| \| *TSPAN15* \| \| *TSPAN2* \| \| *TSPAN3* \| \| *TSPAN4* \| \| *TSPAN5* \| \| *TSPO* \| \| *TXN* \| \| *UAP1* \| \| *UAP1L1* \| \| *UBASH3B* \| \| *UBXN10* \| \| *UCP2* \| \| *UNC5B* \| \| *UNG* \| \| *UPK3B* \| \| *UTP20* \| \| *VDAC1* \| \| *VIL1* \| \| *VIPR1* \| \| *VSIG2* \| \| *VSIR* \| \| *VSNL1* \| \| *VSTM2L* \| \| *VSTM5* \| \| *VTCN1* \| \| *WDR45B* \| \| *WFDC21P* \| \| *WFS1* \| \| *WISP2* \| \| *WNK4* \| \| *WNT7A* \| \| *WNT7B* \| \| *XBP1* \| \| *YARS* \| \| *ZBED3* \| \| *ZDHHC11B* \| \| *ZDHHC8P1* \| \| *ZFP36L2* \| \| *ZG16B* \| \| *ZNF385A*   \|  \| \| --- \| \|  \| \|  \| \|  \| \|  \| \|  \| \|  \| \|  \| \|  \| \|  \| \|  \| \|  \| \|  \| \|  \| \|  \| \|  \| \|  \| \|  \| \|  \| \|  \| \|  \| \|  \| \|  \| \|  \| \|  \| \|  \| \|  \| \|  \| \|  \| \|  \| \|  \| \|  \| \|  \| \|  \| \|  \| \|  \| \|  \| \|  \| \|  \| \|  \| \|  \| \|  \| \|  \| \|  \| \|  \| \|  \| \|  \| \|  \| \|  \| \|  \| \|  \| \|  \| \|  \| \|  \| \|  \| \|  \| \|  \| \|  \| \|  \| \|  \| \|  \| \|  \| \|  \| \|  \| \|  \| \|  \| \|  \| \|  \| \|  \| \|  \| \|  \| \|  \| \|  \| \|  \| \|  \| \|  \| \|  \| \|  \| \|  \| \|  \| \|  \| \|  \| \|  \| \|  \| \|  \| \|  \| \|  \| \|  \| \|  \| \|  \| \|  \| \|  \| \|  \| \|  \| \|  \| \|  \| \|  \| \|  \| \|  \| \|  \| \|  \| \|  \| \|  \| \|  \| \|  \| \|  \| \|  \| \|  \| \|  \| \|  \| \|  \| \|  \| \|  \| \|  \| \|  \| \|  \| \|  \| \|  \| \|  \| \|  \| \|  \| \|  \| \|  \| \|  \| \|  \| \|  \| \|  \| \|  \| \|  \| \|  \| \|  \| \|  \| \|  \| \|  \| \|  \| \|  \| \|  \| \|  \| \|  \| \|  \| \|  \| \|  \| \|  \| \|  \| \|  \| \|  \| \|  \| \|  \| \|  \| \|  \| \|  \| \|  \| \|  \| \|  \| \|  \| \|  \| \|  \| \|  \| \|  \| \|  \| \|  \| \|  \| \|  \| \|  \| \|  \| \|  \| \|  \| \|  \| \|  \| \|  \| \|  \| \|  \| \|  \| \|  \| \|  \| \|  \| \|  \| \|  \| \|  \| \|  \| \|  \| \|  \| \|  \| \|  \| \|  \| \|  \| \|  \| \|  \| \|  \| \|  \| \|  \| \|  \| \|  \| \|  \| \|  \| \|  \| \|  \| \|  \| \|  \| \|  \| \|  \| \|  \| \|  \| \|  \| \|  \| \| |
| --- | --- | --- | --- | --- | --- | --- | --- | --- | --- | --- | --- | --- | --- | --- | --- | --- | --- | --- | --- | --- | --- | --- | --- | --- | --- | --- | --- | --- | --- | --- | --- | --- | --- | --- | --- | --- | --- | --- | --- | --- | --- | --- | --- | --- | --- | --- | --- | --- | --- | --- | --- | --- | --- | --- | --- | --- | --- | --- | --- | --- | --- | --- | --- | --- | --- | --- | --- | --- | --- | --- | --- | --- | --- | --- | --- | --- | --- | --- | --- | --- | --- | --- | --- | --- | --- | --- | --- | --- | --- | --- | --- | --- | --- | --- | --- | --- | --- | --- | --- | --- | --- | --- | --- | --- | --- | --- | --- | --- | --- | --- | --- | --- | --- | --- | --- | --- | --- | --- | --- | --- | --- | --- | --- | --- | --- | --- | --- | --- | --- | --- | --- | --- | --- | --- | --- | --- | --- | --- | --- | --- | --- | --- | --- | --- | --- | --- | --- | --- | --- | --- | --- | --- | --- | --- | --- | --- | --- | --- | --- | --- | --- | --- | --- | --- | --- | --- | --- | --- | --- | --- | --- | --- | --- | --- | --- | --- | --- | --- | --- | --- | --- | --- | --- | --- | --- | --- | --- | --- | --- | --- | --- | --- | --- | --- | --- | --- | --- | --- | --- | --- | --- | --- | --- | --- | --- | --- | --- | --- | --- | --- | --- | --- | --- | --- | --- | --- | --- | --- | --- | --- | --- | --- | --- | --- | --- | --- | --- | --- | --- | --- | --- | --- | --- | --- | --- | --- | --- | --- | --- | --- | --- | --- | --- | --- | --- | --- | --- | --- | --- | --- | --- | --- | --- | --- | --- | --- | --- | --- | --- | --- | --- | --- | --- | --- | --- | --- | --- | --- | --- | --- | --- | --- | --- | --- | --- | --- | --- | --- | --- | --- | --- | --- | --- | --- | --- | --- | --- | --- | --- | --- | --- | --- | --- | --- | --- | --- | --- | --- | --- | --- | --- | --- | --- | --- | --- | --- | --- | --- | --- | --- | --- | --- | --- | --- | --- | --- | --- | --- | --- | --- | --- | --- | --- | --- | --- | --- | --- | --- | --- | --- | --- | --- | --- | --- | --- | --- | --- | --- | --- | --- | --- | --- | --- | --- | --- | --- | --- | --- | --- | --- | --- | --- | --- | --- | --- | --- | --- | --- | --- | --- | --- | --- | --- | --- | --- | --- | --- | --- | --- | --- | --- | --- | --- | --- | --- | --- | --- | --- | --- | --- | --- | --- | --- | --- | --- | --- | --- | --- | --- | --- | --- | --- | --- | --- | --- | --- | --- | --- | --- | --- | --- | --- | --- | --- | --- | --- | --- | --- | --- | --- | --- | --- | --- | --- | --- | --- | --- | --- | --- | --- | --- | --- | --- | --- | --- | --- | --- | --- | --- | --- | --- | --- | --- | --- | --- | --- | --- | --- | --- | --- | --- | --- | --- | --- | --- | --- | --- | --- | --- | --- | --- | --- | --- | --- | --- | --- | --- | --- | --- | --- | --- | --- | --- | --- | --- | --- | --- | --- | --- | --- | --- | --- | --- | --- | --- | --- | --- | --- | --- | --- | --- | --- | --- | --- | --- | --- | --- | --- | --- | --- | --- | --- | --- | --- | --- | --- | --- | --- | --- | --- | --- | --- | --- | --- | --- | --- | --- | --- | --- | --- | --- | --- | --- | --- | --- | --- | --- | --- | --- | --- | --- | --- | --- | --- | --- | --- | --- | --- | --- | --- | --- | --- | --- | --- | --- | --- | --- | --- | --- | --- | --- | --- | --- | --- | --- | --- | --- | --- | --- | --- | --- | --- | --- | --- | --- | --- | --- | --- | --- | --- | --- | --- | --- | --- | --- | --- | --- | --- | --- | --- | --- | --- | --- | --- | --- | --- | --- | --- | --- | --- | --- | --- | --- | --- | --- | --- | --- | --- | --- | --- | --- | --- | --- | --- | --- | --- | --- | --- | --- | --- | --- | --- | --- | --- | --- | --- | --- | --- | --- | --- | --- | --- | --- | --- | --- | --- | --- | --- | --- | --- | --- | --- | --- | --- | --- | --- | --- | --- | --- | --- | --- | --- | --- | --- | --- | --- | --- | --- | --- | --- | --- | --- | --- | --- | --- | --- | --- | --- | --- | --- | --- | --- | --- | --- | --- | --- | --- | --- | --- | --- | --- | --- | --- | --- | --- | --- | --- | --- | --- | --- | --- | --- | --- | --- | --- | --- | --- | --- | --- | --- | --- | --- | --- | --- | --- | --- | --- | --- | --- | --- | --- | --- | --- | --- | --- | --- | --- | --- | --- | --- | --- | --- | --- | --- | --- | --- | --- | --- | --- | --- | --- | --- | --- | --- | --- | --- | --- | --- | --- | --- | --- | --- | --- | --- | --- | --- | --- | --- | --- | --- | --- | --- | --- | --- | --- | --- | --- | --- | --- | --- | --- | --- | --- | --- | --- | --- | --- | --- | --- | --- | --- | --- | --- | --- | --- | --- | --- | --- | --- | --- | --- | --- | --- | --- | --- | --- | --- | --- | --- | --- | --- | --- | --- | --- | --- | --- | --- | --- | --- | --- | --- | --- | --- | --- | --- | --- | --- | --- | --- | --- | --- | --- | --- | --- | --- | --- | --- | --- | --- | --- | --- | --- | --- | --- | --- | --- | --- | --- | --- | --- | --- | --- | --- | --- | --- | --- | --- | --- | --- | --- | --- | --- | --- | --- | --- | --- | --- | --- | --- | --- | --- | --- | --- | --- | --- | --- | --- | --- | --- | --- | --- | --- | --- | --- | --- | --- | --- | --- | --- | --- | --- | --- | --- | --- | --- | --- | --- | --- | --- | --- | --- | --- | --- | --- | --- | --- | --- | --- | --- | --- | --- | --- | --- | --- | --- | --- | --- | --- | --- | --- | --- | --- | --- | --- | --- | --- | --- | --- | --- | --- | --- | --- | --- | --- | --- | --- | --- | --- | --- | --- | --- | --- | --- | --- | --- | --- | --- | --- | --- | --- | --- | --- | --- | --- | --- | --- | --- | --- | --- | --- | --- | --- | --- | --- | --- | --- | --- | --- | --- | --- | --- | --- | --- | --- | --- | --- | --- | --- | --- | --- | --- | --- | --- | --- | --- | --- | --- | --- | --- | --- | --- | --- | --- | --- | --- | --- | --- | --- | --- | --- | --- | --- | --- | --- | --- | --- | --- | --- | --- | --- | --- | --- | --- | --- | --- | --- | --- | --- | --- | --- | --- | --- | --- | --- | --- | --- | --- | --- | --- | --- | --- | --- | --- | --- | --- | --- | --- | --- | --- | --- | --- | --- | --- | --- | --- | --- | --- | --- | --- | --- | --- | --- | --- | --- | --- | --- | --- | --- | --- | --- | --- | --- | --- | --- | --- | --- | --- | --- | --- | --- | --- | --- | --- | --- | --- | --- | --- | --- | --- | --- | --- | --- | --- | --- | --- | --- | --- | --- | --- | --- | --- | --- | --- | --- | --- | --- | --- | --- | --- | --- | --- | --- | --- | --- | --- | --- | --- | --- | --- | --- | --- | --- | --- | --- | --- | --- | --- | --- | --- | --- | --- | --- | --- | --- | --- | --- | --- | --- | --- | --- | --- | --- | --- | --- | --- | --- | --- | --- | --- | --- | --- | --- | --- | --- | --- | --- | --- | --- | --- | --- | --- | --- | --- | --- | --- | --- | --- | --- | --- | --- | --- | --- | --- | --- | --- | --- | --- | --- | --- | --- | --- | --- | --- | --- | --- | --- | --- | --- | --- | --- | --- | --- | --- | --- | --- | --- | --- | --- | --- | --- | --- | --- | --- | --- | --- | --- | --- | --- | --- | --- | --- | --- | --- | --- | --- | --- | --- | --- | --- | --- | --- | --- | --- | --- | --- | --- | --- | --- | --- | --- | --- | --- | --- | --- | --- | --- | --- | --- | --- | --- | --- | --- | --- | --- | --- | --- | --- | --- | --- | --- | --- | --- | --- | --- | --- | --- | --- | --- | --- | --- | --- | --- | --- | --- | --- | --- | --- | --- | --- | --- | --- | --- | --- | --- | --- | --- | --- | --- | --- | --- | --- | --- | --- | --- | --- | --- | --- | --- | --- | --- | --- | --- | --- | --- | --- | --- | --- | --- | --- | --- | --- | --- | --- | --- | --- | --- | --- | --- | --- | --- | --- | --- | --- | --- | --- | --- | --- | --- | --- | --- | --- | --- | --- | --- | --- | --- | --- | --- | --- | --- | --- | --- | --- | --- | --- | --- | --- | --- | --- | --- | --- | --- | --- | --- | --- | --- | --- | --- | --- | --- | --- | --- | --- | --- | --- | --- | --- | --- | --- | --- | --- | --- | --- | --- | --- | --- | --- | --- | --- | --- | --- | --- | --- | --- | --- | --- | --- | --- | --- | --- | --- | --- | --- | --- | --- | --- | --- | --- | --- | --- | --- | --- | --- | --- | --- | --- | --- | --- | --- | --- | --- | --- | --- | --- | --- | --- | --- | --- | --- | --- | --- | --- | --- | --- | --- | --- | --- | --- | --- | --- | --- | --- | --- | --- | --- | --- | --- | --- | --- | --- | --- | --- | --- | --- | --- | --- | --- | --- | --- | --- | --- | --- | --- | --- | --- | --- | --- | --- | --- | --- | --- | --- | --- | --- | --- | --- | --- | --- | --- | --- | --- | --- | --- | --- | --- | --- | --- | --- | --- | --- | --- | --- | --- | --- | --- | --- | --- | --- | --- | --- | --- | --- | --- | --- | --- | --- | --- | --- | --- | --- | --- | --- | --- | --- | --- | --- | --- | --- | --- | --- | --- | --- | --- | --- | --- | --- | --- | --- | --- | --- | --- | --- | --- | --- | --- | --- | --- | --- | --- | --- | --- | --- | --- | --- | --- | --- | --- | --- | --- | --- | --- | --- | --- | --- | --- | --- | --- | --- | --- | --- | --- | --- | --- | --- | --- | --- | --- | --- | --- | --- | --- | --- | --- | --- | --- | --- | --- | --- | --- | --- | --- | --- | --- | --- | --- | --- | --- | --- | --- | --- | --- | --- | --- | --- | --- | --- | --- | --- | --- | --- | --- | --- | --- | --- | --- | --- | --- | --- | --- | --- | --- | --- | --- | --- | --- | --- | --- | --- | --- | --- | --- | --- | --- | --- | --- | --- | --- | --- | --- | --- | --- | --- | --- | --- | --- | --- | --- | --- | --- | --- | --- | --- | --- | --- | --- | --- | --- | --- | --- | --- | --- | --- | --- | --- | --- | --- | --- | --- | --- | --- | --- | --- | --- | --- | --- | --- | --- | --- | --- | --- | --- | --- | --- | --- | --- | --- | --- | --- | --- | --- | --- | --- | --- | --- | --- | --- | --- | --- | --- | --- | --- | --- | --- | --- | --- | --- | --- | --- | --- | --- | --- | --- | --- | --- | --- | --- | --- | --- | --- | --- | --- | --- | --- | --- | --- | --- | --- | --- | --- | --- | --- | --- | --- | --- | --- | --- | --- | --- | --- | --- | --- | --- | --- | --- | --- | --- | --- | --- | --- | --- | --- | --- | --- | --- | --- | --- | --- | --- | --- | --- | --- | --- | --- | --- | --- | --- | --- | --- | --- | --- | --- | --- | --- | --- | --- | --- | --- | --- | --- | --- | --- | --- | --- | --- | --- | --- | --- | --- | --- | --- | --- | --- | --- | --- | --- | --- | --- | --- | --- | --- | --- | --- | --- | --- | --- | --- | --- | --- | --- | --- | --- | --- | --- | --- | --- | --- | --- | --- | --- | --- | --- | --- | --- | --- | --- | --- | --- | --- | --- | --- | --- | --- | --- | --- | --- | --- | --- | --- | --- | --- | --- | --- | --- | --- | --- | --- | --- | --- | --- | --- | --- | --- | --- | --- | --- | --- | --- | --- | --- | --- | --- | --- | --- | --- | --- | --- | --- | --- | --- | --- | --- | --- | --- | --- | --- | --- | --- | --- | --- | --- | --- | --- | --- | --- | --- | --- | --- | --- | --- | --- | --- | --- | --- | --- | --- | --- | --- | --- | --- | --- | --- | --- | --- | --- | --- | --- | --- | --- | --- | --- | --- | --- | --- | --- | --- | --- | --- | --- | --- | --- | --- | --- | --- | --- | --- | --- | --- | --- | --- | --- | --- | --- | --- | --- | --- | --- | --- | --- | --- | --- | --- | --- | --- | --- | --- | --- | --- | --- | --- | --- | --- | --- | --- | --- | --- | --- | --- | --- | --- | --- | --- | --- | --- | --- | --- | --- | --- | --- | --- | --- | --- | --- | --- | --- | --- | --- | --- | --- | --- | --- | --- | --- | --- | --- | --- | --- | --- | --- | --- | --- | --- | --- | --- | --- | --- | --- | --- | --- | --- | --- | --- | --- | --- | --- | --- | --- | --- | --- | --- | --- | --- | --- | --- | --- | --- | --- | --- | --- | --- | --- | --- | --- | --- | --- | --- | --- | --- | --- | --- | --- | --- | --- | --- | --- | --- | --- | --- | --- | --- | --- | --- | --- | --- | --- | --- | --- | --- | --- | --- | --- | --- | --- | --- | --- | --- | --- | --- | --- | --- | --- | --- | --- | --- | --- | --- | --- | --- | --- | --- | --- | --- | --- | --- | --- | --- | --- | --- | --- | --- | --- | --- | --- | --- | --- | --- | --- | --- | --- | --- | --- | --- | --- | --- | --- | --- | --- | --- | --- | --- | --- | --- | --- | --- | --- | --- | --- | --- | --- | --- | --- | --- | --- | --- | --- | --- | --- | --- | --- | --- | --- | --- | --- | --- | --- | --- | --- | --- | --- | --- | --- | --- | --- | --- | --- | --- | --- | --- | --- | --- | --- | --- | --- | --- | --- | --- | --- | --- | --- | --- | --- | --- | --- | --- | --- | --- | --- | --- | --- | --- | --- | --- | --- | --- | --- | --- | --- | --- | --- | --- | --- | --- | --- | --- | --- | --- | --- | --- | --- | --- | --- | --- | --- | --- | --- | --- | --- | --- | --- | --- | --- | --- | --- | --- | --- | --- | --- | --- | --- | --- | --- | --- | --- | --- | --- | --- | --- | --- | --- | --- | --- | --- | --- | --- | --- | --- | --- | --- | --- | --- | --- | --- | --- | --- | --- | --- | --- | --- | --- | --- | --- | --- | --- | --- | --- | --- | --- | --- | --- | --- | --- | --- | --- | --- | --- | --- | --- | --- | --- | --- | --- | --- | --- | --- | --- | --- | --- | --- | --- | --- | --- | --- | --- | --- | --- | --- | --- | --- | --- | --- | --- | --- | --- | --- | --- | --- | --- | --- | --- | --- | --- | --- | --- | --- | --- | --- | --- | --- | --- | --- | --- | --- | --- | --- | --- | --- | --- | --- | --- | --- | --- | --- | --- | --- | --- | --- | --- | --- | --- | --- | --- | --- | --- | --- | --- | --- | --- | --- | --- | --- | --- | --- | --- | --- | --- | --- | --- | --- | --- | --- | --- | --- | --- | --- | --- | --- | --- | --- | --- | --- | --- | --- | --- | --- | --- | --- | --- | --- | --- | --- | --- | --- | --- | --- | --- | --- | --- | --- | --- | --- | --- | --- | --- | --- | --- | --- | --- | --- | --- | --- | --- | --- | --- | --- | --- | --- | --- | --- | --- | --- | --- | --- | --- | --- | --- | --- | --- | --- | --- | --- | --- | --- | --- | --- | --- | --- | --- | --- | --- | --- | --- | --- | --- | --- | --- | --- | --- | --- | --- | --- | --- | --- | --- | --- | --- | --- | --- | --- | --- | --- | --- | --- | --- | --- | --- | --- | --- | --- | --- | --- | --- | --- | --- | --- | --- | --- | --- | --- | --- | --- | --- | --- | --- | --- | --- | --- | --- | --- | --- | --- | --- | --- | --- | --- | --- | --- | --- | --- | --- | --- | --- | --- | --- | --- | --- | --- | --- | --- | --- | --- | --- | --- | --- | --- | --- | --- | --- | --- | --- | --- | --- | --- | --- | --- | --- | --- | --- | --- | --- | --- | --- | --- | --- | --- | --- | --- | --- | --- | --- | --- | --- | --- | --- | --- | --- | --- | --- | --- | --- | --- | --- | --- | --- | --- | --- | --- | --- | --- | --- | --- | --- | --- | --- | --- | --- | --- | --- | --- | --- | --- | --- | --- | --- | --- | --- | --- | --- | --- | --- | --- | --- | --- | --- | --- | --- | --- | --- | --- | --- | --- | --- | --- | --- | --- | --- | --- | --- | --- | --- | --- | --- | --- | --- | --- | --- | --- | --- | --- | --- | --- | --- | --- | --- | --- | --- | --- | --- | --- | --- | --- | --- | --- | --- | --- | --- | --- | --- | --- | --- | --- | --- | --- | --- | --- | --- | --- | --- | --- | --- | --- | --- | --- | --- | --- | --- | --- | --- | --- | --- | --- | --- | --- | --- | --- | --- | --- | --- | --- | --- | --- | --- | --- | --- | --- | --- | --- | --- | --- | --- | --- | --- | --- | --- |

**Genes regulated by calcitriol in RECTAL TUMOR organoids**

| \| *AASS* \| \| --- \| \| *A1CF* \| \| *AASS* \| \| *AATBC* \| \| *ABCA13* \| \| *ABCB1* \| \| *ABCB4* \| \| *ABCC1* \| \| *AC008870.2* \| \| *AC021218.1* \| \| *AC114488.1* \| \| *ACADM* \| \| *ACTR2* \| \| *ADAM22* \| \| *ADAMTS15* \| \| *ADD3* \| \| *AFF4* \| \| *AIG1* \| \| *AKAP11* \| \| *AKAP12* \| \| *AKIRIN1* \| \| *AL390728.4* \| \| *ALOX5* \| \| *AMD1* \| \| *ANK3* \| \| *ANKRD12* \| \| *ANKRD27* \| \| *ANKRD36* \| \| *AP3M1* \| \| *APPL2* \| \| *ARAP2* \| \| *ARGLU1* \| \| *ARHGAP5* \| \| *ARHGEF12* \| \| *ARHGEF28* \| \| *ARHGEF38* \| \| *ARID2* \| \| *ARID4A* \| \| *ARID4B* \| \| *ARL5B* \| \| *ARRDC3* \| \| *ASPH* \| \| *ATAD5* \| \| *ATF2* \| \| *ATM* \| \| *ATP11C* \| \| *ATP2B1* \| \| *ATP6V0A4* \| \| *ATRX* \| \| *ATXN7* \| \| *AZIN1* \| \| *B3GNT5* \| \| *BARD1* \| \| *BAZ1A* \| \| *BAZ2B* \| \| *BBS10* \| \| *BBX* \| \| *BCAS1* \| \| *BCL2L11* \| \| *BCLAF1* \| \| *BIRC3* \| \| *BLVRA* \| \| *BRCA1* \| \| *BRIP1* \| \| *BRWD1* \| \| *BRWD3* \| \| *BTAF1* \| \| *BTBD3* \| \| *C17orf77* \| \| *C1orf131* \| \| *C3orf52* \| \| *C5orf24* \| \| *C5orf51* \| \| *C6orf15* \| \| *C9orf72* \| \| *CA2* \| \| *CA9* \| \| *CALB1* \| \| *CALB2* \| \| *CAMSAP2* \| \| *CAPN7* \| \| *CAPZA2* \| \| *CASD1* \| \| *CASP8AP2* \| \| *CAV1* \| \| *CAV2* \| \| *CCAR1* \| \| *CCDC14* \| \| *CCDC18-AS1* \| \| *CCDC82* \| \| *CCDC88A* \| \| *CCL2* \| \| *CCND1* \| \| *CCNT2* \| \| *CCP110* \| \| *CCSER2* \| \| *CD14* \| \| *CD164* \| \| *CD2AP* \| \| *CD46* \| \| *CDA* \| \| *CDC14A* \| \| *CDC42SE2* \| \| *CDH1* \| \| *CDK12* \| \| *CDK17* \| \| *CDK19* \| \| *CDON* \| \| *CDR2* \| \| *CDYL* \| \| *CDYL2* \| \| *CEP120* \| \| *CEP135* \| \| *CEP170* \| \| *CEP350* \| \| *CFAP97* \| \| *CFLAR* \| \| *CHD7* \| \| *CHD9* \| \| *CHDH* \| \| *CHM* \| \| *CHML* \| \| *CHURC1* \| \| *CIP2A* \| \| *CKAP2* \| \| *CLASP2* \| \| *CLCF1* \| \| *CLDN12* \| \| *CLIP1* \| \| *CLMN* \| \| *CMTR2* \| \| *CPE* \| \| *CPEB2* \| \| *CRACD* \| \| *CRYBG2* \| \| *CRYBG3* \| \| *CSGALNACT2* \| \| *CSPP1* \| \| *CTDSPL2* \| \| *CTTNBP2NL* \| \| *CXCL8* \| \| *CYB5R4* \| \| *CYP24A1* \| \| *CYP3A4* \| \| *CYP3A5* \| \| *CYP3A7* \| \| *CYP4F3* \| \| *DAAM1* \| \| *DACT1* \| \| *DCAF17* \| \| *DCBLD2* \| \| *DCK* \| \| *DCLRE1C* \| \| *DDX17* \| \| *DDX18* \| \| *DDX21* \| \| *DDX5* \| \| *DDX6* \| \| *DDX60* \| \| *DDX60L* \| \| *DEGS1* \| \| *DENND1B* \| \| *DENND4C* \| \| *DENND6B* \| \| *DEPDC1B* \| \| *DGKH* \| \| *DHFR* \| \| *DICER1* \| \| *DLG1* \| \| *DLGAP1-AS1* \| \| *DMTF1* \| \| *DMXL2* \| \| *DNA2* \| \| *DNAJC10* \| \| *DOCK1* \| \| *DOCK5* \| \| *DOCK7* \| \| *DOP1A* \| \| *DPP4* \| \| *DPY19L1* \| \| *DPY19L4* \| \| *DUSP10* \| \| *DYNC1LI2* \| \| *ECT2* \| \| *EFL1* \| \| *EFR3A* \| \| *EGLN1* \| \| *EHBP1* \| \| *EHF* \| \| *EIF2AK2* \| \| *EIF4G2* \| \| *ELF1* \| \| *ELF4* \| \| *ELOVL5* \| \| *ELOVL7* \| \| *EMB* \| \| *ENAH* \| \| *EP300* \| \| *EPC2* \| \| *EPM2AIP1* \| \| *ERBIN* \| \| *EREG* \| \| *ERI2* \| \| *ERMP1* \| \| *ETS1* \| \| *FADS3* \| \| *FAM133B* \| \| *FAM135A* \| \| *FAM13B* \| \| *FAM217B* \| \| *FAM227A* \| \| *FAM76B* \| \| *FAM83B* \| \| *FAM91A1* \| \| *FBXL17* \| \| *FBXO28* \| \| *FBXO30* \| \| *FCHO2* \| \| *FER1L6* \| \| *FEZF1-AS1* \| \| *FGD4* \| \| *FGD6* \| \| *FIGNL1* \| \| *FLG* \| \| *FN1* \| \| *FNBP1L* \| \| *FNDC3A* \| \| *FNIP1* \| \| *FOCAD* \| \| *FOS* \| \| *FRS2* \| \| *FRYL* \| \| *FSD1L* \| \| *FSIP2* \| \| *FTH1* \| \| *FTH1P20* \| \| *FTH1P7* \| \| *FTH1P8* \| \| *G2E3* \| \| *G3BP2* \| \| *GALNT5* \| \| *GALNT7* \| \| *GATAD1* \| \| *GCC2* \| \| *GCSAM* \| \| *GDE1* \| \| *GEM* \| \| *GEN1* \| \| *GFM1* \| \| *GK5* \| \| *GNE* \| \| *GPSM2* \| \| *GPX8* \| \| *GRAMD4* \| \| *GRHL1* \| \| *GRK5* \| \| *GULP1* \| \| *GXYLT1* \| \| *HACD2* \| \| *HAUS3* \| \| *HCP5* \| \| *HECTD4* \| \| *HERC3* \| \| *HIF1A* \| \| *HIPK3* \| \| *HIVEP2* \| \| *HNF4G* \| \| *HOOK3* \| \| *HPS3* \| \| *HRCT1* \| \| *HRNR* \| \| *HS6ST2* \| \| *HSD3B1* \| \| *HSPA13* \| \| *IBTK* \| \| *IFT81* \| \| *IL6ST* \| \| *IMPA1* \| \| *INPP4B* \| \| *INTS6L* \| \| *IQCB1* \| \| *IREB2* \| \| *IRF8* \| \| *ISM1* \| \| *ITGA2* \| \| *ITGB8* \| \| *ITM2B* \| \| *ITSN1* \| \| *JCAD* \| \| *JMJD1C* \| \| *JRKL* \| \| *KAZN* \| \| *KCTD1* \| \| *KDM1B* \| \| *KIAA1324L* \| \| *KIAA1841* \| \| *KIDINS220* \| \| *KIF21A* \| \| *KLF11* \| \| *KLHL5* \| \| *KLK4* \| \| *KLK5* \| \| *KLK6* \| \| *KLK7* \| \| *KMT2C* \| \| *KNL1* \| \| *KNTC1* \| \| *KRAS* \| \| *KRIT1* \| \| *KRT16* \| \| *KYNU* \| \| *LACC1* \| \| *LANCL1* \| \| *LARP4* \| \| *LATS1* \| \| *LBH* \| \| *LEMD3* \| \| *LGR4* \| \| *LGR5* \| \| *LINC00649* \| \| *LINC00992* \| \| *LINC01301* \| \| *LINC01559* \| \| *LINC02474* \| \| *LMTK2* \| \| *LPCAT1* \| \| *LRBA* \| \| *LRP12* \| \| *LRRC58* \| \| *LRRC8B* \| \| *LRRCC1* \| \| *LRRN4* \| \| *LTN1* \| \| *MAB21L4* \| \| *MACC1* \| \| *MAN1A1* \| \| *MAN2A1* \| \| *MAOB* \| \| *MAP3K20* \| \| *MAP3K21* \| \| *MAP4K3* \| \| *MAP4K5* \| \| *MAPK6* \| \| *MARCHF7* \| \| *MAT2A* \| \| *MATR3* \| \| *MCM8* \| \| *MDM2* \| \| *MED13* \| \| *MELTF* \| \| *MELTF-AS1* \| \| *MERTK* \| \| *MFSD6* \| \| *MGAM2* \| \| *MIER3* \| \| *MIGA1* \| \| *MIS18BP1* \| \| *MLF1* \| \| *MMD* \| \| *MME* \| \| *MMP24* \| \| *MMP7* \| \| *MMS22L* \| \| *MOB1B* \| \| *MON2* \| \| *MORC3* \| \| *MORC4* \| \| *MOSMO* \| \| *MPHOSPH6* \| \| *MPP5* \| \| *MSH2* \| \| *MSH3* \| \| *MSI1* \| \| *MT-ATP8* \| \| *MT-ND4L* \| \| *MT-ND5* \| \| *MTBP* \| \| *MTDH* \| \| *MTPN* \| \| *MTUS1* \| \| *MTX3* \| \| *MUC5B* \| \| *MYO6* \| \| *MYRIP* \| \| *MYSM1* \| \| *N4BP2* \| \| *NAA16* \| \| *NAB1* \| \| *NBN* \| \| *NBPF1* \| \| *NCEH1* \| \| *NCKAP1* \| \| *NDRG4* \| \| *NECTIN1* \| \| *NEDD1* \| \| *NEK1* \| \| *NEK7* \| \| *NEMP2* \| \| *NF1* \| \| *NLRC5* \| \| *NNT* \| \| *None* \| \| *None* \| \| *None* \| \| *None* \| \| *NOX1* \| \| *NR1D2* \| \| *NRCAM* \| \| *NSD3* \| \| *NTSR1* \| \| *NUP58* \| \| *NUP62CL* \| \| *NXPE3* \| \| *OIP5-AS1* \| \| *OPHN1* \| \| *OSBPL3* \| \| *OSBPL8* \| \| *OTUD4* \| \| *OXCT1* \| \| *PADI1* \| \| *PADI3* \| \| *PAN3* \| \| *PAXBP1* \| \| *PBRM1* \| \| *PCMTD1* \| \| *PCNX4* \| \| *PDE3B* \| \| *PEAK1* \| \| *PEX1* \| \| *PEX13* \| \| *PFDN4* \| \| *PFN2* \| \| *PGM2* \| \| *PGM2L1* \| \| *PHF10* \| \| *PHF6* \| \| *PHF8* \| \| *PHIP* \| \| *PIK3C2A* \| \| *PIK3CA* \| \| *PIK3R1* \| \| *PIKFYVE* \| \| *PIWIL1* \| \| *PJA2* \| \| *PKD2* \| \| *PKHD1* \| \| *PKN2* \| \| *PLB1* \| \| *PLCB1* \| \| *PLEKHA5* \| \| *PLEKHA7* \| \| *PLK2* \| \| *PM20D2* \| \| *PNISR* \| \| *PNPLA8* \| \| *POLI* \| \| *POLK* \| \| *PPIP5K2* \| \| *PPP1R12A* \| \| *PPP4R2* \| \| *PRELID3A* \| \| *PRKCE* \| \| *PRKDC* \| \| *PRPF40A* \| \| *PRPF4B* \| \| *PRR5L* \| \| *PRSS33* \| \| *PTBP3* \| \| *PTPN21* \| \| *PTPRM* \| \| *PTPRR* \| \| *QSER1* \| \| *RAB11FIP1* \| \| *RAB37* \| \| *RABGAP1L* \| \| *RAD50* \| \| *RADX* \| \| *RALGAPB* \| \| *RALGPS2* \| \| *RANBP6* \| \| *RAP2A* \| \| *RAPGEF4* \| \| *RARRES1* \| \| *RASAL2* \| \| *RASEF* \| \| *RASL11A* \| \| *RB1* \| \| *RBL1* \| \| *RBM25* \| \| *RBM41* \| \| *RBPJ* \| \| *RC3H1* \| \| *RCBTB2* \| \| *RDX* \| \| *RECQL* \| \| *REEP3* \| \| *REL* \| \| *RESF1* \| \| *REV3L* \| \| *RFWD3* \| \| *RHOBTB3* \| \| *RHPN2* \| \| *RIF1* \| \| *RIMBP2* \| \| *RIOK3* \| \| *RNF103* \| \| *RNF128* \| \| *RNF19A* \| \| *RNPC3* \| \| *ROCK1* \| \| *RP2* \| \| *RPS6KA3* \| \| *RYBP* \| \| *SACS* \| \| *SAMD12* \| \| *SCAF11* \| \| *SCML1* \| \| *SCOC* \| \| *SCRN3* \| \| *SCYL2* \| \| *SEC14L1* \| \| *SECISBP2L* \| \| *SEMA3B* \| \| *SEMA3C* \| \| *SEMA6A* \| \| *SERINC2* \| \| *SERPINB1* \| \| *SETD7* \| \| *SGMS2* \| \| *SGPP2* \| \| *SH2D5* \| \| *SH3BGRL2* \| \| *SH3BP4* \| \| *SH3PXD2B* \| \| *SHH* \| \| *SIK1* \| \| *SIRPA* \| \| *SIRT1* \| \| *SLC17A4* \| \| *SLC1A1* \| \| *SLC25A36* \| \| *SLC30A10* \| \| *SLC30A9* \| \| *SLC34A3* \| \| *SLC35D1* \| \| *SLC37A2* \| \| *SLC38A1* \| \| *SLC38A2* \| \| *SLC39A10* \| \| *SLC44A1* \| \| *SLC44A5* \| \| *SLC4A7* \| \| *SLC5A9* \| \| *SLCO4A1-AS1* \| \| *SLFN5* \| \| *SMARCA5* \| \| *SMARCAD1* \| \| *SMC4* \| \| *SMC5* \| \| *SMC6* \| \| *SMG1* \| \| *SMIM14* \| \| *SNRK* \| \| *SNX30* \| \| *SOX4* \| \| *SOX6* \| \| *SP3* \| \| *SPATS2L* \| \| *SPDL1* \| \| *SPTBN5* \| \| *SREK1* \| \| *SRFBP1* \| \| *SRGAP1* \| \| *SRI* \| \| *STAT1* \| \| *STEAP2* \| \| *STEAP4* \| \| *STK26* \| \| *STK3* \| \| *STK38L* \| \| *STRADB* \| \| *STX17* \| \| *STXBP3* \| \| *SULT1C2* \| \| *SUZ12* \| \| *SVIL* \| \| *SYT1* \| \| *SYT12* \| \| *TAB3* \| \| *TAF2* \| \| *TAF9B* \| \| *TAOK1* \| \| *TARBP1* \| \| *TASOR2* \| \| *TBC1D8B* \| \| *TBILA* \| \| *TCF12* \| \| *TEAD1* \| \| *TET3* \| \| *TFAP2C* \| \| *TFRC* \| \| *TIA1* \| \| *TIMP2* \| \| *TIPARP* \| \| *TJP1* \| \| *TLR4* \| \| *TMEM106B* \| \| *TMEM131* \| \| *TMEM144* \| \| *TMEM150C* \| \| *TMEM209* \| \| *TMF1* \| \| *TMTC3* \| \| *TMTC4* \| \| *TMX3* \| \| *TNFAIP3* \| \| *TNKS2* \| \| *TNPO1* \| \| *TNRC6A* \| \| *TOPBP1* \| \| *TPST1* \| \| *TRAK2* \| \| *TRAM1* \| \| *TRANK1* \| \| *TRAPPC8* \| \| *TRIM33* \| \| *TRIM38* \| \| *TRIM56* \| \| *TRMT1L* \| \| *TRPM7* \| \| *TRPV6* \| \| *TSPYL4* \| \| *TTC14* \| \| *TTC9* \| \| *TTPAL* \| \| *TUBA1A* \| \| *TUG1* \| \| *TWF1* \| \| *TXNRD1* \| \| *U2SURP* \| \| *UBLCP1* \| \| *UBXN4* \| \| *UBXN7* \| \| *UCA1* \| \| *UFL1* \| \| *UGDH* \| \| *UGT8* \| \| *UHMK1* \| \| *USP1* \| \| *USP12* \| \| *USP16* \| \| *USP33* \| \| *USP34* \| \| *USP45* \| \| *USP53* \| \| *VEZF1* \| \| *VPS13A* \| \| *VPS13B* \| \| *VPS36* \| \| *VRK2* \| \| *WAPL* \| \| *WASHC2C* \| \| *WASHC4* \| \| *WDFY3* \| \| *WEE1* \| \| *WNK1* \| \| *WRN* \| \| *WSB1* \| \| *XIST* \| \| *XKRX* \| \| *XPO1* \| \| *XPR1* \| \| *XRCC2* \| \| *XRN1* \| \| *YAP1* \| \| *YBX2* \| \| *YTHDC2* \| \| *YTHDF3* \| \| *ZBTB11* \| \| *ZBTB26* \| \| *ZBTB33* \| \| *ZBTB41* \| \| *ZC3H12C* \| \| *ZDHHC17* \| \| *ZFYVE16* \| \| *ZKSCAN8* \| \| *ZMYND11* \| \| *ZNF107* \| \| *ZNF117* \| \| *ZNF197* \| \| *ZNF215* \| \| *ZNF217* \| \| *ZNF252P* \| \| *ZNF292* \| \| *ZNF302* \| \| *ZNF320* \| \| *ZNF326* \| \| *ZNF33B* \| \| *ZNF449* \| \| *ZNF512B* \| \| *ZNF518A* \| \| *ZNF525* \| \| *ZNF561* \| \| *ZNF608* \| \| *ZNF611* \| \| *ZNF644* \| \| *ZNF664* \| \| *ZNF675* \| \| *ZNF714* \| \| *ZNF761* \| \| *ZNF770* \| \| *ZNF780A* \| \| *ZNF780B* \| \| *ZNF83* \| \| *ZNF841* \| \| *ZNF91* \| \| *ZNF92* \| \| *ZRANB2* \| \| *A2M* \| \| *AATK* \| \| *ABCB8* \| \| *ABCG1* \| \| *ABHD11-AS1* \| \| *ABLIM3* \| \| *ABTB1* \| \| *AC004080.1* \| \| *AC008870.1* \| \| *AC010442.1* \| \| *AC016735.1* \| \| *AC024293.1* \| \| *AC104958.2* \| \| *AC131097.2* \| \| *AC132812.1* \| \| *ACAA1* \| \| *ACOT8* \| \| *ACOX2* \| \| *ACP2* \| \| *ACP5* \| \| *ACSF2* \| \| *ACSM3* \| \| *ACSS2* \| \| *ADAMTSL4* \| \| *ADCK2* \| \| *ADORA2B* \| \| *ADPRHL2* \| \| *ADRA2C* \| \| *ADRB2* \| \| *AGPAT2* \| \| *AGR2* \| \| *AGR3* \| \| *AGTRAP* \| \| *AIFM3* \| \| *AIP* \| \| *AL391056.1* \| \| *ALDH3A1* \| \| *ALDH4A1* \| \| *ALDOC* \| \| *AMN* \| \| *ANG* \| \| *ANGPTL4* \| \| *ANKRD9* \| \| *ANTKMT* \| \| *ANXA1* \| \| *ANXA6* \| \| *AP1S1* \| \| *AP2S1* \| \| *AP3S2* \| \| *APOBR* \| \| *APOC1* \| \| *APOD* \| \| *APOE* \| \| *APRT* \| \| *AQP3* \| \| *ARAP3* \| \| *ARHGAP27* \| \| *ARHGEF16* \| \| *ARHGEF40* \| \| *ARL2* \| \| *ARRB1* \| \| *ASAP3* \| \| *ASB4* \| \| *ASGR1* \| \| *ASIC1* \| \| *ASL* \| \| *ASPHD2* \| \| *ASS1* \| \| *ATF4* \| \| *ATG101* \| \| *ATG9A* \| \| *ATP1A1* \| \| *ATP2A3* \| \| *ATP2C2* \| \| *ATP5F1D* \| \| *ATP5MC1* \| \| *ATP5ME* \| \| *ATP5MF* \| \| *ATP5PO* \| \| *ATP6V0B* \| \| *ATP6V1F* \| \| *ATRAID* \| \| *AURKAIP1* \| \| *AVPI1* \| \| *AXIN1* \| \| *AZGP1* \| \| *B4GALT3* \| \| *B4GAT1* \| \| *BABAM1* \| \| *BACE2* \| \| *BAD* \| \| *BAIAP2L2* \| \| *BAIAP3* \| \| *BAX* \| \| *BBC3* \| \| *BCAM* \| \| *BCAP31* \| \| *BCAT2* \| \| *BCL2L14* \| \| *BDH1* \| \| *BHLHE40* \| \| *BHLHE41* \| \| *BIN1* \| \| *BLVRB* \| \| *BMP4* \| \| *BOK* \| \| *BSG* \| \| *C10orf99* \| \| *C11orf68* \| \| *C12orf10* \| \| *C16orf74* \| \| *C19orf57* \| \| *C2* \| \| *C2orf72* \| \| *C3orf85* \| \| *C4BPB* \| \| *C4orf48* \| \| *C6orf223* \| \| *C9orf152* \| \| *CAB39L* \| \| *CABP1* \| \| *CAMK2B* \| \| *CAMKK1* \| \| *CAPN5* \| \| *CAPN8* \| \| *CBLC* \| \| *CC2D1A* \| \| *CCDC12* \| \| *CCDC22* \| \| *CCDC3* \| \| *CCDC61* \| \| *CCDC69* \| \| *CCDC85B* \| \| *CCDC88B* \| \| *CCL24* \| \| *CCNJL* \| \| *CD276* \| \| *CD320* \| \| *CD44* \| \| *CD74* \| \| *CD99* \| \| *CDC25B* \| \| *CDC42EP1* \| \| *CDC42EP2* \| \| *CDC42EP4* \| \| *CDC42EP5* \| \| *CDHR1* \| \| *CDK10* \| \| *CDKN1C* \| \| *CDX1* \| \| *CEACAM1* \| \| *CEMIP* \| \| *CFD* \| \| *CFTR* \| \| *CHCHD10* \| \| *CHCHD6* \| \| *CHKA* \| \| *CHPF* \| \| *CHST13* \| \| *CIAO2B* \| \| *CIB1* \| \| *CILP2* \| \| *CISH* \| \| *CKB* \| \| *CLDN3* \| \| *CLIC1* \| \| *CLPP* \| \| *CLRN3* \| \| *CLTB* \| \| *CNFN* \| \| *CNN2* \| \| *COA3* \| \| *COA8* \| \| *COASY* \| \| *COL5A1* \| \| *COL6A1* \| \| *COL7A1* \| \| *COMTD1* \| \| *COPRS* \| \| *COQ8A* \| \| *CORO1A* \| \| *COX14* \| \| *COX5B* \| \| *COX6B1* \| \| *COX8A* \| \| *CPNE1* \| \| *CRABP2* \| \| *CRAT* \| \| *CRB3* \| \| *CRYL1* \| \| *CRYM* \| \| *CTDNEP1* \| \| *CTDSP1* \| \| *CTU2* \| \| *CX3CL1* \| \| *CXCR4* \| \| *CXXC5* \| \| *CYB5B* \| \| *CYC1* \| \| *CYP2J2* \| \| *CYSTM1* \| \| *CYTOR* \| \| *DAPL1* \| \| *DBI* \| \| *DCPS* \| \| *DCXR* \| \| *DDAH2* \| \| *DEDD2* \| \| *DEGS2* \| \| *DGAT1* \| \| *DGKA* \| \| *DGKQ* \| \| *DHRS3* \| \| *DHRS4L2* \| \| *DIO3* \| \| *DIO3OS* \| \| *DKK4* \| \| *DMKN* \| \| *DNAJC15* \| \| *DNASE2* \| \| *DNM2* \| \| *DNPH1* \| \| *DOHH* \| \| *DOK4* \| \| *DPCD* \| \| *DPEP1* \| \| *DPM3* \| \| *DRAP1* \| \| *DRD2* \| \| *DTX2* \| \| *DUOX2* \| \| *DUSP4* \| \| *DUSP6* \| \| *EBP* \| \| *ECM1* \| \| *ECSIT* \| \| *EEPD1* \| \| *EFNA1* \| \| *EFNA3* \| \| *EFNB1* \| \| *EGFL7* \| \| *EGLN3* \| \| *EIF4EBP1* \| \| *ELFN1-AS1* \| \| *EML2* \| \| *EML3* \| \| *EMP3* \| \| *ENKD1* \| \| *ENTPD6* \| \| *EPHX1* \| \| *EPN3* \| \| *ETFB* \| \| *ETHE1* \| \| *ETV4* \| \| *EVA1C* \| \| *EVPL* \| \| *EVX1* \| \| *EXOSC4* \| \| *EXOSC7* \| \| *F10* \| \| *FA2H* \| \| *FAAH* \| \| *FABP1* \| \| *FAIM2* \| \| *FAM114A1* \| \| *FAM117A* \| \| *FAM162A* \| \| *FAM166C* \| \| *FAM207A* \| \| *FAM3D* \| \| *FBXL15* \| \| *FBXO2* \| \| *FBXO25* \| \| *FBXO32* \| \| *FBXO6* \| \| *FFAR4* \| \| *FGFBP1* \| \| *FGFR3* \| \| *FGFR4* \| \| *FHL3* \| \| *FIS1* \| \| *FLNC* \| \| *FOLR1* \| \| *FOXA3* \| \| *FUOM* \| \| *FUT1* \| \| *FUT3* \| \| *FXYD3* \| \| *FZD7* \| \| *GADD45B* \| \| *GADD45GIP1* \| \| *GAK* \| \| *GAL* \| \| *GCAT* \| \| *GCHFR* \| \| *GET3* \| \| *GGT6* \| \| *GHDC* \| \| *GIPC1* \| \| *GJB1* \| \| *GJC2* \| \| *GLIS2* \| \| *GLYCTK* \| \| *GMDS* \| \| *GNB1L* \| \| *GNG4* \| \| *GOLT1A* \| \| *GOT1* \| \| *GPA33* \| \| *GPR108* \| \| *GPR157* \| \| *GPR35* \| \| *GPRC5C* \| \| *GPRIN2* \| \| *GPX2* \| \| *GRK2* \| \| *GRN* \| \| *GSDMB* \| \| *GSTM3* \| \| *GSTO1* \| \| *GSTP1* \| \| *GUK1* \| \| *H1-0* \| \| *H2AJ* \| \| *HAGH* \| \| *HAGHL* \| \| *HAX1* \| \| *HDAC5* \| \| *HES1* \| \| *HES6* \| \| *HIGD2A* \| \| *HLA-DMB* \| \| *HMBS* \| \| *HMGCS2* \| \| *HMOX1* \| \| *HOXA11* \| \| *HOXA11-AS* \| \| *HOXB13* \| \| *HOXB5* \| \| *HPDL* \| \| *HSD11B1L* \| \| *HSD11B2* \| \| *HSPB1* \| \| *HSPBP1* \| \| *ICAM3* \| \| *ID1* \| \| *ID4* \| \| *IDH3B* \| \| *IER2* \| \| *IER3* \| \| *IFI27* \| \| *IFI27L2* \| \| *IFITM1* \| \| *IFITM2* \| \| *IFITM3* \| \| *IGFBP4* \| \| *IGFL4* \| \| *IGSF8* \| \| *IGSF9* \| \| *IL27RA* \| \| *IL2RG* \| \| *IL37* \| \| *IMPDH1* \| \| *INAFM1* \| \| *INPP5D* \| \| *IRS2* \| \| *ISG15* \| \| *ISOC2* \| \| *ISX* \| \| *ISYNA1* \| \| *ITGB2-AS1* \| \| *ITGB5* \| \| *ITGB7* \| \| *ITM2C* \| \| *ITPK1* \| \| *ITPKA* \| \| *JAG1* \| \| *JSRP1* \| \| *KALRN* \| \| *KAZALD1* \| \| *KCNG1* \| \| *KCNJ4* \| \| *KCNQ1* \| \| *KDM7A-DT* \| \| *KHK* \| \| *KIFC3* \| \| *KISS1* \| \| *KLF2* \| \| *KRTCAP3* \| \| *KYAT1* \| \| *LAMA4* \| \| *LAMB2* \| \| *LAMTOR4* \| \| *LCK* \| \| *LCN2* \| \| *LCP1* \| \| *LDHD* \| \| *LEF1* \| \| *LGALS1* \| \| *LGALS3* \| \| *LHPP* \| \| *LIN7B* \| \| *LINC00482* \| \| *LINC00963* \| \| *LINC01133* \| \| *LINC01315* \| \| *LINC01843* \| \| *LINC02086* \| \| *LLGL2* \| \| *LMNA* \| \| *LNX1* \| \| *LPCAT4* \| \| *LRG1* \| \| *LRP10* \| \| *LRPAP1* \| \| *LRRC42* \| \| *LSM2* \| \| *LTBP4* \| \| *LY6D* \| \| *LY6E* \| \| *LY6G6C* \| \| *LY6G6D* \| \| *LY6G6F-LY6G6D* \| \| *LYPD6B* \| \| *LYPLA2* \| \| *LYRM1* \| \| *LYZ* \| \| *MACROD1* \| \| *MAF1* \| \| *MAP2K2* \| \| *MAPRE3* \| \| *MAST1* \| \| *MDH2* \| \| *ME3* \| \| *MED16* \| \| *MEP1A* \| \| *METTL26* \| \| *MFSD10* \| \| *MFSD3* \| \| *MGAT3* \| \| *MGAT4B* \| \| *MHENCR* \| \| *MICALL2* \| \| *MICOS13* \| \| *MLPH* \| \| *MOB2* \| \| *MOSPD3* \| \| *MPST* \| \| *MRPL12* \| \| *MRPL14* \| \| *MRPL2* \| \| *MRPL23* \| \| *MRPL24* \| \| *MRPL27* \| \| *MRPL28* \| \| *MRPL34* \| \| *MRPL4* \| \| *MRPL48* \| \| *MRPL52* \| \| *MRPL54* \| \| *MRPL55* \| \| *MRPS12* \| \| *MRPS34* \| \| *MSLN* \| \| *MT1A* \| \| *MTLN* \| \| *MUC1* \| \| *MUC13* \| \| *MUC2* \| \| *MVB12A* \| \| *MVB12B* \| \| *MVD* \| \| *MYCN* \| \| *MYDGF* \| \| *MYL6* \| \| *MZT2A* \| \| *MZT2B* \| \| *NAA10* \| \| *NAA38* \| \| *NAA80* \| \| *NAB2* \| \| *NAGLU* \| \| *NAGPA* \| \| *NBL1* \| \| *NCBP2AS2* \| \| *NCLN* \| \| *NDRG2* \| \| *NDUFA11* \| \| *NDUFA13* \| \| *NDUFA9* \| \| *NDUFB11* \| \| *NDUFB7* \| \| *NDUFB8* \| \| *NDUFB9* \| \| *NDUFS3* \| \| *NDUFS7* \| \| *NDUFS8* \| \| *NDUFV1* \| \| *NECTIN2* \| \| *NELFE* \| \| *NELL2* \| \| *NES* \| \| *NFATC4* \| \| *NIBAN2* \| \| *NKD2* \| \| *NMB* \| \| *NMU* \| \| *NMUR2* \| \| *NOC4L* \| \| *NOD2* \| \| *NOP10* \| \| *NOSTRIN* \| \| *NOXA1* \| \| *NPM3* \| \| *NPW* \| \| *NR1D1* \| \| *NR1H3* \| \| *NR2F6* \| \| *NR4A1* \| \| *NRARP* \| \| *NSMF* \| \| *NT5C* \| \| *NT5E* \| \| *NTMT1* \| \| *NUCB1* \| \| *NXPH3* \| \| *NYNRIN* \| \| *OAS1* \| \| *OAS2* \| \| *OAZ1* \| \| *OBSCN* \| \| *ORMDL3* \| \| *OSBPL5* \| \| *OSBPL7* \| \| *P3H2* \| \| *P3H4* \| \| *P4HA2* \| \| *PAQR7* \| \| *PARVB* \| \| *PBX4* \| \| *PC* \| \| *PCCA* \| \| *PCK2* \| \| *PCSK9* \| \| *PDE4A* \| \| *PDE4C* \| \| *PDE9A* \| \| *PDLIM2* \| \| *PDLIM7* \| \| *PDZD3* \| \| *PELP1* \| \| *PEMT* \| \| *PER1* \| \| *PF4* \| \| *PFKFB4* \| \| *PGM1* \| \| *PHC2* \| \| *PHETA1* \| \| *PHF1* \| \| *PHGR1* \| \| *PIGR* \| \| *PIGT* \| \| *PIH1D1* \| \| *PIM3* \| \| *PIR* \| \| *PITPNM3* \| \| *PLA2G15* \| \| *PLAC8* \| \| *PLBD1* \| \| *PLCD1* \| \| *PLD3* \| \| *PLEK2* \| \| *PLEKHH3* \| \| *PLEKHM1* \| \| *PLIN2* \| \| *PLOD1* \| \| *PLP2* \| \| *PLPP2* \| \| *PLPPR2* \| \| *PLTP* \| \| *PLXNA2* \| \| *PMF1* \| \| *PMM1* \| \| *PMVK* \| \| *PNPLA6* \| \| *POLR2E* \| \| *POLR2H* \| \| *POLR2J* \| \| *POLRMT* \| \| *POU5F1B* \| \| *PPARD* \| \| *PPARG* \| \| *PPFIA3* \| \| *PPIA* \| \| *PPP1R13L* \| \| *PPP1R14D* \| \| *PPP1R16A* \| \| *PPP4C* \| \| *PRDX1* \| \| *PRDX5* \| \| *PRELID1* \| \| *PRF1* \| \| *PRKAB1* \| \| *PRSS23* \| \| *PRSS3* \| \| *PRSS8* \| \| *PSCA* \| \| *PSD4* \| \| *PSMB6* \| \| *PSMD13* \| \| *PSMG3* \| \| *PTCHD1* \| \| *PTGER2* \| \| *PTMS* \| \| *PTP4A3* \| \| *PTPRN2* \| \| *PTPRU* \| \| *PTTG1* \| \| *PUF60* \| \| *PWWP2B* \| \| *PYCR1* \| \| *PYGB* \| \| *PYM1* \| \| *QPCTL* \| \| *QPRT* \| \| *R3HDM4* \| \| *RAB11FIP5* \| \| *RAB25* \| \| *RAB32* \| \| *RABAC1* \| \| *RAP1GAP* \| \| *RAPGEF3* \| \| *RARG* \| \| *RASA3* \| \| *RASAL1* \| \| *RASIP1* \| \| *RBCK1* \| \| *RDH10* \| \| *REEP6* \| \| *REG4* \| \| *RGS14* \| \| *RHBDD2* \| \| *RHOV* \| \| *RHPN1* \| \| *RILP* \| \| *RILPL2* \| \| *RIMKLA* \| \| *RIN1* \| \| *RLBP1* \| \| *RNASE1* \| \| *RNASE6* \| \| *RNF167* \| \| *RNF186* \| \| *RNF208* \| \| *RNF223* \| \| *RNF24* \| \| *RNH1* \| \| *RNPEPL1* \| \| *RORC* \| \| *RPL13P12* \| \| *RPL18AP3* \| \| *RPL23A* \| \| *RPS6KA1* \| \| *RRAS* \| \| *RUVBL2* \| \| *RXFP4* \| \| *RXRA* \| \| *S100A10* \| \| *S100A14* \| \| *S100A4* \| \| *S100P* \| \| *SARS1* \| \| *SAT1* \| \| *SAT2* \| \| *SBSPON* \| \| *SCAMP3* \| \| *SCAND1* \| \| *SCRN2* \| \| *SCYL1* \| \| *SDHAF1* \| \| *SEC13* \| \| *SELENOO* \| \| *SELENOW* \| \| *SEMA7A* \| \| *SERF2* \| \| *SERPINE2* \| \| *SERTAD1* \| \| *SESN2* \| \| *SEZ6L2* \| \| *SF3B5* \| \| *SFN* \| \| *SGSM3* \| \| *SH3BP1* \| \| *SHMT2* \| \| *SIGIRR* \| \| *SIPA1* \| \| *SIRT2* \| \| *SIRT6* \| \| *SKAP1* \| \| *SLC14A1* \| \| *SLC17A9* \| \| *SLC25A1* \| \| *SLC25A26* \| \| *SLC25A42* \| \| *SLC26A9* \| \| *SLC27A5* \| \| *SLC29A3* \| \| *SLC35E4* \| \| *SLC38A5* \| \| *SLC39A3* \| \| *SLC39A4* \| \| *SLC39A5* \| \| *SLC39A7* \| \| *SLC3A1* \| \| *SLC3A2* \| \| *SLC40A1* \| \| *SLC44A2* \| \| *SLC44A4* \| \| *SLC45A3* \| \| *SLC50A1* \| \| *SLC52A2* \| \| *SLC52A3* \| \| *SLC5A1* \| \| *SLC6A7* \| \| *SLC7A7* \| \| *SLC7A8* \| \| *SLC9A3R2* \| \| *SLCO2B1* \| \| *SLPI* \| \| *SMAD6* \| \| *SMAGP* \| \| *SMIM1* \| \| *SMIM12* \| \| *SMIM24* \| \| *SMYD3* \| \| *SNCG* \| \| *SNHG15* \| \| *SNHG18* \| \| *SNORC* \| \| *SNRPD2* \| \| *SNU13* \| \| *SOD3* \| \| *SP6* \| \| *SPAG7* \| \| *SPATC1L* \| \| *SPDEF* \| \| *SPINK4* \| \| *SPINT1* \| \| *SPINT2* \| \| *SPON2* \| \| *SPR* \| \| *SPRY2* \| \| *SPSB1* \| \| *SPTSSB* \| \| *SQOR* \| \| *SREBF1* \| \| *SRMS* \| \| *SRPX2* \| \| *SSBP3* \| \| *ST3GAL2* \| \| *ST3GAL4* \| \| *ST3GAL5* \| \| *ST6GALNAC1* \| \| *ST6GALNAC2* \| \| *ST6GALNAC6* \| \| *STARD10* \| \| *STING1* \| \| *STK11* \| \| *STK16* \| \| *STK32C* \| \| *STN1* \| \| *STOML2* \| \| *STXBP2* \| \| *SUGCT* \| \| *SULT1B1* \| \| *SWI5* \| \| *SYNE3* \| \| *SYNE4* \| \| *SYNGR2* \| \| *SYNPO* \| \| *SYTL1* \| \| *TACO1* \| \| *TBC1D10A* \| \| *TBC1D17* \| \| *TBRG4* \| \| *TCEA3* \| \| *TCN1* \| \| *TCN2* \| \| *TDGF1* \| \| *TECR* \| \| *TESC* \| \| *TFEB* \| \| *TFF1* \| \| *TFF2* \| \| *TGFB1* \| \| *THAP4* \| \| *THEM6* \| \| *THRA* \| \| *TIMM13* \| \| *TIMP1* \| \| *TIMP3* \| \| *TJP3* \| \| *TMBIM1* \| \| *TMC4* \| \| *TMED3* \| \| *TMED9* \| \| *TMEM101* \| \| *TMEM109* \| \| *TMEM120A* \| \| *TMEM141* \| \| *TMEM147* \| \| *TMEM159* \| \| *TMEM160* \| \| *TMEM161A* \| \| *TMEM205* \| \| *TMEM208* \| \| *TMEM214* \| \| *TMEM229B* \| \| *TMEM238L* \| \| *TMEM256* \| \| *TMEM45B* \| \| *TMEM54* \| \| *TMEM63B* \| \| *TMEM63C* \| \| *TMEM9* \| \| *TMEM92* \| \| *TMSB10* \| \| *TMSB4X* \| \| *TMUB1* \| \| *TNFRSF14* \| \| *TNFRSF1B* \| \| *TNK2* \| \| *TNNC1* \| \| *TNS4* \| \| *TOM1L2* \| \| *TOX2* \| \| *TPD52L1* \| \| *TPRA1* \| \| *TPRG1L* \| \| *TPST2* \| \| *TRAPPC1* \| \| *TREH* \| \| *TRIB3* \| \| *TRIM15* \| \| *TRIM16* \| \| *TRIM3* \| \| *TRIM7* \| \| *TRIP10* \| \| *TRPM4* \| \| *TSC22D1* \| \| *TSC22D3* \| \| *TSPAN15* \| \| *TSPO* \| \| *TSR3* \| \| *TST* \| \| *TUBA4A* \| \| *TUFM* \| \| *TWF2* \| \| *TXN2* \| \| *TXNL4A* \| \| *TYRO3* \| \| *UBAC2* \| \| *UBBP4* \| \| *UBE2M* \| \| *UBXN6* \| \| *UNC5B* \| \| *UPK3A* \| \| *UPK3B* \| \| *UQCC2* \| \| *UQCR10* \| \| *UQCR11* \| \| *UQCRC1* \| \| *UQCRFS1* \| \| *UQCRQ* \| \| *UXT* \| \| *VASP* \| \| *VEGFB* \| \| *VILL* \| \| *VIPR1* \| \| *VPS25* \| \| *VPS28* \| \| *VSIG2* \| \| *VSIR* \| \| *WDR13* \| \| *WDR18* \| \| *WDR83OS* \| \| *WFS1* \| \| *WNK4* \| \| *WNT7B* \| \| *XAB2* \| \| *XYLT2* \| \| *YDJC* \| \| *YIF1A* \| \| *YIF1B* \| \| *YIPF2* \| \| *YJU2* \| \| *ZBTB7B* \| \| *ZDHHC3* \| \| *ZDHHC8P1* \| \| *ZFAND2B* \| \| *ZFP36L2* \| \| *ZFYVE28* \| \| *ZNF385A* \| \| *ZNF511* \| \| *ZNF524* \| \| *ZNF579* \| \| *ZNF692* \| \| *ZNHIT1* \| \| *ZNHIT2* \| \| \|  \| \| --- \| \|  \| \|  \| \|  \| \|  \| \|  \| \|  \| \|  \| \|  \| \|  \| \|  \| \|  \| \|  \| \|  \| \|  \| \|  \| \|  \| \|  \| \|  \| \|  \| \|  \| \|  \| \|  \| \|  \| \|  \| \|  \| \|  \| \|  \| \|  \| \|  \| \|  \| \|  \| \|  \| \|  \| \|  \| \|  \| \|  \| \|  \| \|  \| \|  \| \|  \| \|  \| \|  \| \|  \| \|  \| \|  \| \|  \| \|  \| \|  \| \|  \| \|  \| \|  \| \|  \| \|  \| \|  \| \|  \| \|  \| \|  \| \|  \| \|  \| \|  \| \|  \| \|  \| \|  \| \|  \| \|  \| \|  \| \|  \| \|  \| \|  \| \|  \| \|  \| \|  \| \|  \| \|  \| \|  \| \|  \| \|  \| \|  \| \|  \| \|  \| \|  \| \|  \| \|  \| \|  \| \|  \| \|  \| \|  \| \|  \| \|  \| \|  \| \|  \| \|  \| \|  \| \|  \| \|  \| \|  \| \|  \| \|  \| \|  \| \|  \| \|  \| \|  \| \|  \| \|  \| \|  \| \|  \| \|  \| \|  \| \|  \| \|  \| \|  \| \|  \| \|  \| \|  \| \|  \| \|  \| \|  \| \|  \| \|  \| \|  \| \|  \| \|  \| \|  \| \|  \| \|  \| \|  \| \|  \| \|  \| \|  \| \|  \| \|  \| \|  \| \|  \| \|  \| \|  \| \|  \| \|  \| \|  \| \|  \| \|  \| \|  \| \|  \| \|  \| \|  \| \|  \| \|  \| \|  \| \|  \| \|  \| \|  \| \|  \| \|  \| \|  \| \|  \| \|  \| \|  \| \|  \| \|  \| \|  \| \|  \| \|  \| \|  \| \|  \| \|  \| \|  \| \|  \| \|  \| \|  \| \|  \| \|  \| \|  \| \|  \| \|  \| \|  \| \|  \| \|  \| \|  \| \|  \| \|  \| \|  \| \|  \| \|  \| \|  \| \|  \| \|  \| \|  \| \|  \| \|  \| \|  \| \|  \| \|  \| \|  \| \|  \| \|  \| \|  \| \|  \| \|  \| \|  \| \|  \| \|  \| \|  \| \|  \| \|  \| \|  \| \|  \| \| |
| --- | --- | --- | --- | --- | --- | --- | --- | --- | --- | --- | --- | --- | --- | --- | --- | --- | --- | --- | --- | --- | --- | --- | --- | --- | --- | --- | --- | --- | --- | --- | --- | --- | --- | --- | --- | --- | --- | --- | --- | --- | --- | --- | --- | --- | --- | --- | --- | --- | --- | --- | --- | --- | --- | --- | --- | --- | --- | --- | --- | --- | --- | --- | --- | --- | --- | --- | --- | --- | --- | --- | --- | --- | --- | --- | --- | --- | --- | --- | --- | --- | --- | --- | --- | --- | --- | --- | --- | --- | --- | --- | --- | --- | --- | --- | --- | --- | --- | --- | --- | --- | --- | --- | --- | --- | --- | --- | --- | --- | --- | --- | --- | --- | --- | --- | --- | --- | --- | --- | --- | --- | --- | --- | --- | --- | --- | --- | --- | --- | --- | --- | --- | --- | --- | --- | --- | --- | --- | --- | --- | --- | --- | --- | --- | --- | --- | --- | --- | --- | --- | --- | --- | --- | --- | --- | --- | --- | --- | --- | --- | --- | --- | --- | --- | --- | --- | --- | --- | --- | --- | --- | --- | --- | --- | --- | --- | --- | --- | --- | --- | --- | --- | --- | --- | --- | --- | --- | --- | --- | --- | --- | --- | --- | --- | --- | --- | --- | --- | --- | --- | --- | --- | --- | --- | --- | --- | --- | --- | --- | --- | --- | --- | --- | --- | --- | --- | --- | --- | --- | --- | --- | --- | --- | --- | --- | --- | --- | --- | --- | --- | --- | --- | --- | --- | --- | --- | --- | --- | --- | --- | --- | --- | --- | --- | --- | --- | --- | --- | --- | --- | --- | --- | --- | --- | --- | --- | --- | --- | --- | --- | --- | --- | --- | --- | --- | --- | --- | --- | --- | --- | --- | --- | --- | --- | --- | --- | --- | --- | --- | --- | --- | --- | --- | --- | --- | --- | --- | --- | --- | --- | --- | --- | --- | --- | --- | --- | --- | --- | --- | --- | --- | --- | --- | --- | --- | --- | --- | --- | --- | --- | --- | --- | --- | --- | --- | --- | --- | --- | --- | --- | --- | --- | --- | --- | --- | --- | --- | --- | --- | --- | --- | --- | --- | --- | --- | --- | --- | --- | --- | --- | --- | --- | --- | --- | --- | --- | --- | --- | --- | --- | --- | --- | --- | --- | --- | --- | --- | --- | --- | --- | --- | --- | --- | --- | --- | --- | --- | --- | --- | --- | --- | --- | --- | --- | --- | --- | --- | --- | --- | --- | --- | --- | --- | --- | --- | --- | --- | --- | --- | --- | --- | --- | --- | --- | --- | --- | --- | --- | --- | --- | --- | --- | --- | --- | --- | --- | --- | --- | --- | --- | --- | --- | --- | --- | --- | --- | --- | --- | --- | --- | --- | --- | --- | --- | --- | --- | --- | --- | --- | --- | --- | --- | --- | --- | --- | --- | --- | --- | --- | --- | --- | --- | --- | --- | --- | --- | --- | --- | --- | --- | --- | --- | --- | --- | --- | --- | --- | --- | --- | --- | --- | --- | --- | --- | --- | --- | --- | --- | --- | --- | --- | --- | --- | --- | --- | --- | --- | --- | --- | --- | --- | --- | --- | --- | --- | --- | --- | --- | --- | --- | --- | --- | --- | --- | --- | --- | --- | --- | --- | --- | --- | --- | --- | --- | --- | --- | --- | --- | --- | --- | --- | --- | --- | --- | --- | --- | --- | --- | --- | --- | --- | --- | --- | --- | --- | --- | --- | --- | --- | --- | --- | --- | --- | --- | --- | --- | --- | --- | --- | --- | --- | --- | --- | --- | --- | --- | --- | --- | --- | --- | --- | --- | --- | --- | --- | --- | --- | --- | --- | --- | --- | --- | --- | --- | --- | --- | --- | --- | --- | --- | --- | --- | --- | --- | --- | --- | --- | --- | --- | --- | --- | --- | --- | --- | --- | --- | --- | --- | --- | --- | --- | --- | --- | --- | --- | --- | --- | --- | --- | --- | --- | --- | --- | --- | --- | --- | --- | --- | --- | --- | --- | --- | --- | --- | --- | --- | --- | --- | --- | --- | --- | --- | --- | --- | --- | --- | --- | --- | --- | --- | --- | --- | --- | --- | --- | --- | --- | --- | --- | --- | --- | --- | --- | --- | --- | --- | --- | --- | --- | --- | --- | --- | --- | --- | --- | --- | --- | --- | --- | --- | --- | --- | --- | --- | --- | --- | --- | --- | --- | --- | --- | --- | --- | --- | --- | --- | --- | --- | --- | --- | --- | --- | --- | --- | --- | --- | --- | --- | --- | --- | --- | --- | --- | --- | --- | --- | --- | --- | --- | --- | --- | --- | --- | --- | --- | --- | --- | --- | --- | --- | --- | --- | --- | --- | --- | --- | --- | --- | --- | --- | --- | --- | --- | --- | --- | --- | --- | --- | --- | --- | --- | --- | --- | --- | --- | --- | --- | --- | --- | --- | --- | --- | --- | --- | --- | --- | --- | --- | --- | --- | --- | --- | --- | --- | --- | --- | --- | --- | --- | --- | --- | --- | --- | --- | --- | --- | --- | --- | --- | --- | --- | --- | --- | --- | --- | --- | --- | --- | --- | --- | --- | --- | --- | --- | --- | --- | --- | --- | --- | --- | --- | --- | --- | --- | --- | --- | --- | --- | --- | --- | --- | --- | --- | --- | --- | --- | --- | --- | --- | --- | --- | --- | --- | --- | --- | --- | --- | --- | --- | --- | --- | --- | --- | --- | --- | --- | --- | --- | --- | --- | --- | --- | --- | --- | --- | --- | --- | --- | --- | --- | --- | --- | --- | --- | --- | --- | --- | --- | --- | --- | --- | --- | --- | --- | --- | --- | --- | --- | --- | --- | --- | --- | --- | --- | --- | --- | --- | --- | --- | --- | --- | --- | --- | --- | --- | --- | --- | --- | --- | --- | --- | --- | --- | --- | --- | --- | --- | --- | --- | --- | --- | --- | --- | --- | --- | --- | --- | --- | --- | --- | --- | --- | --- | --- | --- | --- | --- | --- | --- | --- | --- | --- | --- | --- | --- | --- | --- | --- | --- | --- | --- | --- | --- | --- | --- | --- | --- | --- | --- | --- | --- | --- | --- | --- | --- | --- | --- | --- | --- | --- | --- | --- | --- | --- | --- | --- | --- | --- | --- | --- | --- | --- | --- | --- | --- | --- | --- | --- | --- | --- | --- | --- | --- | --- | --- | --- | --- | --- | --- | --- | --- | --- | --- | --- | --- | --- | --- | --- | --- | --- | --- | --- | --- | --- | --- | --- | --- | --- | --- | --- | --- | --- | --- | --- | --- | --- | --- | --- | --- | --- | --- | --- | --- | --- | --- | --- | --- | --- | --- | --- | --- | --- | --- | --- | --- | --- | --- | --- | --- | --- | --- | --- | --- | --- | --- | --- | --- | --- | --- | --- | --- | --- | --- | --- | --- | --- | --- | --- | --- | --- | --- | --- | --- | --- | --- | --- | --- | --- | --- | --- | --- | --- | --- | --- | --- | --- | --- | --- | --- | --- | --- | --- | --- | --- | --- | --- | --- | --- | --- | --- | --- | --- | --- | --- | --- | --- | --- | --- | --- | --- | --- | --- | --- | --- | --- | --- | --- | --- | --- | --- | --- | --- | --- | --- | --- | --- | --- | --- | --- | --- | --- | --- | --- | --- | --- | --- | --- | --- | --- | --- | --- | --- | --- | --- | --- | --- | --- | --- | --- | --- | --- | --- | --- | --- | --- | --- | --- | --- | --- | --- | --- | --- | --- | --- | --- | --- | --- | --- | --- | --- | --- | --- | --- | --- | --- | --- | --- | --- | --- | --- | --- | --- | --- | --- | --- | --- | --- | --- | --- | --- | --- | --- | --- | --- | --- | --- | --- | --- | --- | --- | --- | --- | --- | --- | --- | --- | --- | --- | --- | --- | --- | --- | --- | --- | --- | --- | --- | --- | --- | --- | --- | --- | --- | --- | --- | --- | --- | --- | --- | --- | --- | --- | --- | --- | --- | --- | --- | --- | --- | --- | --- | --- | --- | --- | --- | --- | --- | --- | --- | --- | --- | --- | --- | --- | --- | --- | --- | --- | --- | --- | --- | --- | --- | --- | --- | --- | --- | --- | --- | --- | --- | --- | --- | --- | --- | --- | --- | --- | --- | --- | --- | --- | --- | --- | --- | --- | --- | --- | --- | --- | --- | --- | --- | --- | --- | --- | --- | --- | --- | --- | --- | --- | --- | --- | --- | --- | --- | --- | --- | --- | --- | --- | --- | --- | --- | --- | --- | --- | --- | --- | --- | --- | --- | --- | --- | --- | --- | --- | --- | --- | --- | --- | --- | --- | --- | --- | --- | --- | --- | --- | --- | --- | --- | --- | --- | --- | --- | --- | --- | --- | --- | --- | --- | --- | --- | --- | --- | --- | --- | --- | --- | --- | --- | --- | --- | --- | --- | --- | --- | --- | --- | --- | --- | --- | --- | --- | --- | --- | --- | --- | --- | --- | --- | --- | --- | --- | --- | --- | --- | --- | --- | --- | --- | --- | --- | --- | --- | --- | --- | --- | --- | --- | --- | --- | --- | --- | --- | --- | --- | --- | --- | --- | --- | --- | --- | --- | --- | --- | --- | --- | --- | --- | --- | --- | --- | --- | --- | --- | --- | --- | --- | --- | --- | --- | --- | --- | --- | --- | --- | --- | --- | --- | --- | --- | --- | --- | --- | --- | --- | --- | --- | --- | --- | --- | --- | --- | --- | --- | --- | --- | --- | --- | --- | --- | --- | --- | --- | --- | --- | --- | --- | --- | --- | --- | --- | --- | --- | --- | --- | --- | --- | --- | --- | --- | --- | --- | --- | --- | --- | --- | --- | --- | --- | --- | --- | --- | --- | --- | --- | --- | --- | --- | --- | --- | --- | --- | --- | --- | --- | --- | --- | --- | --- | --- | --- | --- | --- | --- | --- | --- | --- | --- | --- | --- | --- | --- | --- | --- | --- | --- | --- | --- | --- | --- | --- | --- | --- | --- | --- | --- | --- | --- | --- | --- | --- | --- | --- | --- | --- | --- | --- | --- | --- | --- | --- | --- | --- | --- | --- | --- | --- | --- | --- | --- | --- | --- | --- | --- | --- | --- | --- | --- | --- | --- | --- | --- | --- | --- | --- | --- | --- | --- | --- | --- | --- | --- | --- | --- | --- | --- | --- | --- | --- | --- | --- | --- | --- | --- | --- | --- | --- | --- | --- | --- | --- | --- | --- | --- | --- | --- | --- | --- | --- | --- | --- | --- | --- | --- | --- | --- | --- | --- | --- | --- | --- | --- | --- | --- | --- | --- | --- | --- | --- | --- | --- | --- | --- | --- | --- | --- | --- | --- | --- | --- | --- | --- | --- | --- | --- | --- | --- | --- | --- | --- | --- | --- | --- | --- | --- | --- | --- | --- | --- | --- | --- | --- | --- | --- | --- | --- | --- | --- | --- | --- | --- | --- | --- | --- | --- | --- | --- | --- | --- | --- | --- | --- | --- | --- | --- | --- | --- | --- | --- | --- | --- | --- | --- | --- | --- | --- | --- | --- | --- | --- | --- | --- | --- | --- | --- | --- | --- | --- | --- | --- | --- | --- | --- | --- | --- | --- | --- | --- | --- | --- | --- | --- | --- | --- | --- | --- | --- | --- | --- | --- | --- | --- | --- | --- | --- | --- | --- | --- | --- | --- | --- | --- | --- | --- | --- | --- | --- | --- | --- | --- | --- | --- | --- | --- | --- | --- | --- | --- | --- | --- | --- | --- | --- | --- | --- | --- | --- | --- | --- | --- | --- | --- | --- | --- | --- | --- | --- | --- | --- | --- | --- | --- | --- | --- | --- | --- | --- | --- | --- | --- | --- | --- | --- | --- | --- | --- | --- | --- | --- | --- | --- | --- | --- | --- | --- | --- | --- | --- | --- | --- | --- | --- | --- | --- | --- | --- | --- | --- | --- | --- | --- | --- | --- | --- | --- | --- | --- | --- | --- | --- | --- | --- | --- | --- | --- | --- | --- | --- | --- | --- | --- | --- | --- | --- | --- | --- | --- | --- | --- | --- | --- | --- | --- | --- | --- | --- | --- | --- | --- | --- | --- |

**Genes regulated by calcitriol in normal COLON organoids (ENDOSCOPY SAMPLES)**

| \| *A1CF* \| \| --- \| \| *AASS* \| \| *ABAT* \| \| *ABCB1* \| \| *ABCB4* \| \| *ABCC1* \| \| *ABCC2* \| \| *ABCD3* \| \| *ABHD3* \| \| *ABL1* \| \| *ABTB2* \| \| *ACAA2* \| \| *ACTR3C* \| \| *ADAM22* \| \| *ADAMTS14* \| \| *ADAMTS15* \| \| *ADAMTS9* \| \| *ADAMTSL5* \| \| *ADGRE5* \| \| *ADGRG7* \| \| *ADGRL2* \| \| *ADGRL3* \| \| *ADHFE1* \| \| *ADO* \| \| *AHRR* \| \| *AIG1* \| \| *AKAP12* \| \| *AKIRIN1* \| \| *AKR1C1* \| \| *AKR1C3* \| \| *ALOX5* \| \| *ALPI* \| \| *AMOTL1* \| \| *AMZ1* \| \| *ANKRD27* \| \| *ANKS4B* \| \| *ANO10* \| \| *ANXA4* \| \| *ANXA5* \| \| *AOC1* \| \| *APBB1* \| \| *APH1B* \| \| *APLF* \| \| *APPL2* \| \| *ARHGAP25* \| \| *ARHGEF28* \| \| *ARHGEF38* \| \| *ARID3B* \| \| *ARL2BP* \| \| *ARL5B* \| \| *ARNT2* \| \| *ATP2B1* \| \| *ATP2B1-AS1* \| \| *ATP8A1* \| \| *B4GALNT2* \| \| *B4GALT1* \| \| *BARX2* \| \| *BCAR1* \| \| *BCAS1* \| \| *BCL2L11* \| \| *BEND7* \| \| *BIRC3* \| \| *BLNK* \| \| *BLVRA* \| \| *BMPR1B* \| \| *BTBD3* \| \| *C15orf48* \| \| *C1orf131* \| \| *C1QTNF1* \| \| *C2orf54* \| \| *C2orf88* \| \| *C3* \| \| *C3orf52* \| \| *C4orf48* \| \| *C6orf141* \| \| *C6orf15* \| \| *CA12* \| \| *CA2* \| \| *CACNA2D3* \| \| *CADPS* \| \| *CALB2* \| \| *CALM1* \| \| *CAMK2D* \| \| *CAMK2G* \| \| *CAPG* \| \| *CAPNS1* \| \| *CARD10* \| \| *CASC9* \| \| *CAVIN1* \| \| *CCL2* \| \| *CD14* \| \| *CD151* \| \| *CD40* \| \| *CDA* \| \| *CDC42SE2* \| \| *CDH1* \| \| *CDH17* \| \| *CDK19* \| \| *CDKN2A* \| \| *CDKN2B* \| \| *CDON* \| \| *CDS1* \| \| *CDX2* \| \| *CEACAM6* \| \| *CEBPD* \| \| *CFLAR* \| \| *CHDH* \| \| *CHRNA7* \| \| *CHST11* \| \| *CLCF1* \| \| *CLCN5* \| \| *CLDN11* \| \| *CLU* \| \| *CMTR2* \| \| *CNTN3* \| \| *COL12A1* \| \| *COL16A1* \| \| *COL18A1* \| \| *COLEC12* \| \| *COMP* \| \| *COX5A* \| \| *CP* \| \| *CPA6* \| \| *CPE* \| \| *CPNE2* \| \| *CPVL* \| \| *CRLF1* \| \| *CRYBG2* \| \| *CSF1* \| \| *CST6* \| \| *CTNNB1* \| \| *CTSD* \| \| *CTSO* \| \| *CTSS* \| \| *CUEDC1* \| \| *CYB5A* \| \| *CYP19A1* \| \| *CYP1B1* \| \| *CYP24A1* \| \| *CYP27A1* \| \| *CYP2B6* \| \| *CYP2B7P* \| \| *CYP3A4* \| \| *CYP3A5* \| \| *CYP3A7* \| \| *CYP4F3* \| \| *CYTH4* \| \| *DAB2* \| \| *DACT1* \| \| *DAPK2* \| \| *DCAF11* \| \| *DCBLD2* \| \| *DDX60* \| \| *DENND1B* \| \| *DENND6B* \| \| *DEPDC1B* \| \| *DGAT2* \| \| *DLGAP1-AS1* \| \| *DMXL2* \| \| *DNAJC5* \| \| *DNER* \| \| *DOCK1* \| \| *DOCK5* \| \| *DOK1* \| \| *DPP4* \| \| *DTNA* \| \| *DTNB* \| \| *DTX3* \| \| *DUSP10* \| \| *DYSF* \| \| *EDN2* \| \| *EFL1* \| \| *EFR3A* \| \| *EGLN2* \| \| *EGR1* \| \| *EGR2* \| \| *EHBP1* \| \| *EHBP1L1* \| \| *EIF6* \| \| *ELF1* \| \| *ELF4* \| \| *ELFN2* \| \| *EMB* \| \| *EMC10* \| \| *ENAH* \| \| *ENC1* \| \| *ENPEP* \| \| *EPHA10* \| \| *EPHA4* \| \| *EPHB4* \| \| *EREG* \| \| *ERMP1* \| \| *ETS2* \| \| *EXOC7* \| \| *EXTL3* \| \| *FABP6* \| \| *FADS3* \| \| *FAM126A* \| \| *FAM131B* \| \| *FAM20A* \| \| *FAM217B* \| \| *FAM227A* \| \| *FAM43A* \| \| *FAM84A* \| \| *FAM86DP* \| \| *FBLIM1* \| \| *FBXO41* \| \| *FCGRT* \| \| *FER1L6* \| \| *FLG* \| \| *FLG-AS1* \| \| *FLG2* \| \| *FLNA* \| \| *FLRT3* \| \| *FMN2* \| \| *FN1* \| \| *FNDC4* \| \| *FOCAD* \| \| *FOS* \| \| *FOSL2* \| \| *FOXK1* \| \| *FREM1* \| \| *FSIP2* \| \| *FSTL1* \| \| *FTH1* \| \| *FTH1P15* \| \| *FTH1P23* \| \| *FTH1P3* \| \| *FTH1P7* \| \| *FYCO1* \| \| *FYN* \| \| *G6PD* \| \| *GABRE* \| \| *GADD45A* \| \| *GALNT5* \| \| *GARNL3* \| \| *GCKR* \| \| *GCSAM* \| \| *GDE1* \| \| *GDF11* \| \| *GDPD3* \| \| *GEM* \| \| *GGT1* \| \| *GLB1L2* \| \| *GLI2* \| \| *GNAI2* \| \| *GNE* \| \| *GNPAT* \| \| *GOLIM4* \| \| *GPAA1* \| \| *GPD1* \| \| *GPN1* \| \| *GPR37* \| \| *GRAMD2A* \| \| *GRAMD4* \| \| *GRHL1* \| \| *GRIP2* \| \| *GRK5* \| \| *GSTA1* \| \| *GUCD1* \| \| *HCN1* \| \| *HCP5* \| \| *HIF1A* \| \| *HIST1H2AC* \| \| *HIVEP2* \| \| *HLA-B* \| \| *HLA-F* \| \| *HMGA1* \| \| *HMGA2* \| \| *HNF4G* \| \| *HNRNPA1P26* \| \| *HRCT1* \| \| *HRNR* \| \| *HSD17B2* \| \| *HSD3B1* \| \| *HSPA12A* \| \| *ICAM1* \| \| *ID3* \| \| *IFITM10* \| \| *IFNE* \| \| *IGFBP6* \| \| *IGFL1P1* \| \| *IGFL2-AS1* \| \| *IL17RB* \| \| *IL1RAPL1* \| \| *IL6R* \| \| *ILDR1* \| \| *INPP1* \| \| *INPP4B* \| \| *INTS6L* \| \| *IQCB1* \| \| *IQUB* \| \| *IRF8* \| \| *ITGB8* \| \| *ITM2B* \| \| *JCAD* \| \| *JUN* \| \| *JUNB* \| \| *KAZN* \| \| *KCNMB4* \| \| *KCTD1* \| \| *KCTD17* \| \| *KDM1A* \| \| *KIAA1211* \| \| *KIAA1324L* \| \| *KIAA1551* \| \| *KIF3C* \| \| *KLHL13* \| \| *KLK13* \| \| *KLK5* \| \| *KLK6* \| \| *KLK7* \| \| *KRT16* \| \| *KRT6B* \| \| *KSR2* \| \| *KYNU* \| \| *LAD1* \| \| *LAMA2* \| \| *LBH* \| \| *LDLRAP1* \| \| *LFNG* \| \| *LGALS3BP* \| \| *LGALS9* \| \| *LGR5* \| \| *LHFPL2* \| \| *LINC00649* \| \| *LINC01559* \| \| *LINC01801* \| \| *LINC02263* \| \| *LINC02474* \| \| *LINC02588* \| \| *LMO3* \| \| *LPCAT2* \| \| *LRIG1* \| \| *LRRC61* \| \| *LRRC66* \| \| *LRRC75B* \| \| *LRRN4* \| \| *LTB* \| \| *LZTS2* \| \| *MACC1* \| \| *MALL* \| \| *MAN2B1* \| \| *MAOB* \| \| *MAP3K12* \| \| *MAP7* \| \| *MAP9* \| \| *MDK* \| \| *MEF2C* \| \| *MEGF8* \| \| *MELTF* \| \| *MELTF-AS1* \| \| *MERTK* \| \| *MFSD1* \| \| *MFSD2A* \| \| *MFSD6* \| \| *MGAT4A* \| \| *MGST1* \| \| *MICAL3* \| \| *MIDN* \| \| *MMD* \| \| *MMP10* \| \| *MMP24* \| \| *MMP7* \| \| *MOGAT1* \| \| *MOGAT2* \| \| *MORC4* \| \| *MOSMO* \| \| *MPHOSPH6* \| \| *MPP5* \| \| *MS4A8* \| \| *MSI1* \| \| *MTTP* \| \| *MX1* \| \| *MYH14* \| \| *NAV3* \| \| *NBEA* \| \| *NBPF1* \| \| *NBPF3* \| \| *NCKAP5* \| \| *NDRG4* \| \| *NEURL3* \| \| *NEXN* \| \| *NFKBIA* \| \| *NHS* \| \| *NIPAL2* \| \| *NLRC5* \| \| *NOV* \| \| *NOX1* \| \| *NPNT* \| \| *NR0B2* \| \| *NR5A2* \| \| *NRCAM* \| \| *NRP2* \| \| *NSUN6* \| \| *NUAK2* \| \| *NUDT4* \| \| *OAF* \| \| *ODF2L* \| \| *OPHN1* \| \| *OPN3* \| \| *PACS1* \| \| *PADI1* \| \| *PAPSS1* \| \| *PARM1* \| \| *PARP12* \| \| *PBX1* \| \| *PCYOX1L* \| \| *PDCD6* \| \| *PDE3B* \| \| *PDGFA* \| \| *PDLIM4* \| \| *PDXK* \| \| *PEX13* \| \| *PFDN4* \| \| *PGM2L1* \| \| *PHACTR3* \| \| *PHETA1* \| \| *PHKA2* \| \| *PIEZO2* \| \| *PIGQ* \| \| *PIK3CA* \| \| *PINK1* \| \| *PITPNC1* \| \| *PITPNM1* \| \| *PKIB* \| \| *PLB1* \| \| *PLEC* \| \| *PLEKHA5* \| \| *PLEKHA6* \| \| *PLEKHA7* \| \| *PLK2* \| \| *PLLP* \| \| *PLPP3* \| \| *PLS1* \| \| *PLSCR1* \| \| *PLXDC2* \| \| *PLXND1* \| \| *PMEPA1* \| \| *POR* \| \| *PPP1R14C* \| \| *PPP1R1C* \| \| *PRAP1* \| \| *PRICKLE1* \| \| *PRKAR1B* \| \| *PRKG2* \| \| *PRMT9* \| \| *PRODH* \| \| *PROS1* \| \| *PRR13* \| \| *PRR5L* \| \| *PRSS22* \| \| *PRSS33* \| \| *PTAFR* \| \| *PTGS2* \| \| *PTK7* \| \| *PYCARD* \| \| *RAB11FIP1* \| \| *RAB37* \| \| *RABGAP1L* \| \| *RAC2* \| \| *RAI2* \| \| *RALGAPB* \| \| *RAMP1* \| \| *RARRES1* \| \| *RARRES2* \| \| *RASAL2* \| \| *RASL11A* \| \| *RASSF10* \| \| *RBP1* \| \| *RBPMS* \| \| *RCAN2* \| \| *RCBTB2* \| \| *RDX* \| \| *REPIN1* \| \| *RHOB* \| \| *RHOD* \| \| *RIMS3* \| \| *RIN3* \| \| *RIPK2* \| \| *RNF103* \| \| *RNF128* \| \| *RNF152* \| \| *RNF19A* \| \| *RNF224* \| \| *RNF38* \| \| *RNF5* \| \| *ROBO1* \| \| *RPS6KA3* \| \| *RPTN* \| \| *RUBCNL* \| \| *RUNDC3B* \| \| *S100G* \| \| *SAA1* \| \| *SAA2* \| \| *SAMD12* \| \| *SARDH* \| \| *SATB1* \| \| *SATB1-AS1* \| \| *SAXO2* \| \| *SCG5* \| \| *SCML1* \| \| *SCN8A* \| \| *SDC3* \| \| *SEC14L1* \| \| *SEC14L6* \| \| *SECTM1* \| \| *SELENON* \| \| *SELENOP* \| \| *SEMA3A* \| \| *SEMA3B* \| \| *SEMA3C* \| \| *SEMA3F* \| \| *SEMA6A* \| \| *SEMA6A-AS1* \| \| *SERINC2* \| \| *SERPINA1* \| \| *SERPINB1* \| \| *SERPINB9* \| \| *SESN3* \| \| *SGK1* \| \| *SGMS2* \| \| *SGPP2* \| \| *SH3BGRL2* \| \| *SH3BP4* \| \| *SH3PXD2A* \| \| *SH3PXD2B* \| \| *SH3RF2* \| \| *SH3TC1* \| \| *SHH* \| \| *SHTN1* \| \| *SI* \| \| *SIK1* \| \| *SIPA1L3* \| \| *SIRPA* \| \| *SLC12A7* \| \| *SLC15A2* \| \| *SLC16A5* \| \| *SLC17A4* \| \| *SLC1A1* \| \| *SLC20A1* \| \| *SLC25A4* \| \| *SLC27A2* \| \| *SLC28A3* \| \| *SLC2A8* \| \| *SLC30A10* \| \| *SLC34A2* \| \| *SLC34A3* \| \| *SLC37A2* \| \| *SLC46A3* \| \| *SLC4A8* \| \| *SLC51B* \| \| *SLC6A12* \| \| *SLC6A20* \| \| *SLC9A1* \| \| *SLCO4A1* \| \| *SLCO4A1-AS1* \| \| *SLFN5* \| \| *SMAD7* \| \| *SMIM14* \| \| *SMOC2* \| \| *SMPD1* \| \| *SNRK* \| \| *SNX30* \| \| *SOX4* \| \| *SPATA2L* \| \| *SPATS2L* \| \| *SPNS2* \| \| *SRGAP1* \| \| *SRI* \| \| *STAP2* \| \| *STAT1* \| \| *STAT3* \| \| *STAT6* \| \| *STEAP3* \| \| *STEAP4* \| \| *STOX2* \| \| *STRADB* \| \| *SULF1* \| \| *SULT1C2* \| \| *SULT1C2P1* \| \| *SULT2B1* \| \| *SUSD6* \| \| *SVIL* \| \| *SYNPR* \| \| *SYT12* \| \| *SYT8* \| \| *SYTL2* \| \| *TAF9B* \| \| *TBC1D1* \| \| *TBC1D9* \| \| *TCAF1* \| \| *TCF12* \| \| *TCTN1* \| \| *TEX15* \| \| *TEX9* \| \| *TFPI* \| \| *TFPI2* \| \| *TFRC* \| \| *TGFB2* \| \| *TGOLN2* \| \| *THBD* \| \| *THRB* \| \| *THSD7A* \| \| *TIMP2* \| \| *TINAGL1* \| \| *TIPARP* \| \| *TJP2* \| \| *TLE4* \| \| *TLR2* \| \| *TLR4* \| \| *TM4SF20* \| \| *TMC6* \| \| *TMEM106B* \| \| *TMEM117* \| \| *TMEM131* \| \| *TMEM132A* \| \| *TMEM158* \| \| *TMEM176A* \| \| *TMEM176B* \| \| *TMEM200A* \| \| *TMEM30B* \| \| *TMEM37* \| \| *TMEM63A* \| \| *TMEM98* \| \| *TMPRSS2* \| \| *TMTC4* \| \| *TNC* \| \| *TNF* \| \| *TNFAIP2* \| \| *TNFAIP3* \| \| *TNFAIP8L3* \| \| *TNNI1* \| \| *TNNT2* \| \| *TNS3* \| \| *TOMM34* \| \| *TOX* \| \| *TPPP3* \| \| *TPST1* \| \| *TRAF4* \| \| *TRAK2* \| \| *TRIB1* \| \| *TRIM35* \| \| *TRIM38* \| \| *TRIM56* \| \| *TRPV6* \| \| *TSC2* \| \| *TSKU* \| \| *TSPAN14* \| \| *TTC9* \| \| *TTYH3* \| \| *TUBA1A* \| \| *TUG1* \| \| *TXNRD1* \| \| *TYMP* \| \| *UBE2E1* \| \| *UBE2E2* \| \| *UCA1* \| \| *UCKL1* \| \| *UGDH* \| \| *UGT2B17* \| \| *UROS* \| \| *USP12* \| \| *USP2* \| \| *USP53* \| \| *VAV1* \| \| *VAV3* \| \| *VEZF1* \| \| *VPS13A* \| \| *VWA3B* \| \| *VWDE* \| \| *WDR55* \| \| *WDR6* \| \| *WDR72* \| \| *WDR78* \| \| *WFDC2* \| \| *WLS* \| \| *WNK1* \| \| *WSCD1* \| \| *WTIP* \| \| *XKRX* \| \| *YAP1* \| \| *YBX2* \| \| *ZBED6CL* \| \| *ZCCHC17* \| \| *ZFAND5* \| \| *ZFPM1* \| \| *ZMIZ1* \| \| *ZNF512B* \| \| *ZNF549* \| \| *ZNF608* \| \| *ZNF703* \| \| *ZNF774* \| \| *ZNF827* \| \| *ZYX* \|  \| *ABHD10* \| \| --- \| \| *ABHD17C* \| \| *ABHD6* \| \| *ABLIM3* \| \| *ACER2* \| \| *ACP5* \| \| *ACSL3* \| \| *ACSL5* \| \| *ACSS1* \| \| *ACSS2* \| \| *ADAMTSL4* \| \| *ADGRF1* \| \| *ADGRG6* \| \| *ADM* \| \| *ADORA2B* \| \| *ADRA2A* \| \| *ADRA2C* \| \| *ADRB1* \| \| *AFAP1L1* \| \| *AGR2* \| \| *AGR3* \| \| *AIF1L* \| \| *AIFM3* \| \| *AK4* \| \| *AK7* \| \| *ALDH1A3* \| \| *ALDH1L1* \| \| *ALDH3A1* \| \| *ALDOC* \| \| *ALG5* \| \| *ALPG* \| \| *ALPP* \| \| *ANG* \| \| *ANGPTL4* \| \| *ANK1* \| \| *ANKEF1* \| \| *ANKRD1* \| \| *ANXA1* \| \| *ANXA3* \| \| *APOBR* \| \| *APOL6* \| \| *AQP1* \| \| *AQP3* \| \| *AQP5* \| \| *ARF6* \| \| *ARFGAP3* \| \| *ARHGAP27* \| \| *ARHGAP27P1-BPTFP1-KPNA2P3* \| \| *ARHGAP6* \| \| *ARHGDIB* \| \| *ARHGEF2* \| \| *ARHGEF40* \| \| *ARL1* \| \| *ARRB1* \| \| *ARRDC2* \| \| *ASIC1* \| \| *ASPHD2* \| \| *ASS1* \| \| *ATL3* \| \| *ATP1A1* \| \| *ATP1B1* \| \| *ATP2A3* \| \| *ATP2C2* \| \| *AXL* \| \| *AZGP1* \| \| *B3GNT7* \| \| *BACE2* \| \| *BAIAP2L1* \| \| *BAIAP2L2* \| \| *BCAR3* \| \| *BCL2L14* \| \| *BHLHA15* \| \| *BHLHE40* \| \| *BHLHE41* \| \| *BICDL1* \| \| *BLCAP* \| \| *BMP7* \| \| *BTG3* \| \| *BTNL9* \| \| *C12orf56* \| \| *C1orf116* \| \| *C2CD4A* \| \| *C2orf72* \| \| *C4BPB* \| \| *C5orf46* \| \| *C6orf223* \| \| *C9orf152* \| \| *CABLES1* \| \| *CACNA1C* \| \| *CALU* \| \| *CAPN2* \| \| *CAPN5* \| \| *CAPN8* \| \| *CAPRIN2* \| \| *CARS* \| \| *CASP7* \| \| *CAST* \| \| *CCK* \| \| *CCL20* \| \| *CCL24* \| \| *CCL28* \| \| *CCND2* \| \| *CD109* \| \| *CD44* \| \| *CD46* \| \| *CD55* \| \| *CD58* \| \| *CD96* \| \| *CDC25B* \| \| *CDC42EP1* \| \| *CDC42EP2* \| \| *CDCA7* \| \| *CDK6* \| \| *CDKN1C* \| \| *CDKN3* \| \| *CDX1* \| \| *CEACAM5* \| \| *CEMIP* \| \| *CEMIP2* \| \| *CENPF* \| \| *CGNL1* \| \| *CHMP2B* \| \| *CKAP4* \| \| *CLCA4* \| \| *CLCN4* \| \| *CLDN10* \| \| *CLDN3* \| \| *CLIC3* \| \| *CLIC4* \| \| *CLN3* \| \| *CMBL* \| \| *COL13A1* \| \| *COL27A1* \| \| *COL4A1* \| \| *COL6A1* \| \| *COX6B2* \| \| *CPT1A* \| \| *CRAT* \| \| *CREB3L1* \| \| *CREB3L2* \| \| *CREG2* \| \| *CRIP2* \| \| *CSGALNACT1* \| \| *CSTB* \| \| *CTNNAL1* \| \| *CTSV* \| \| *CX3CL1* \| \| *CXXC5* \| \| *CYR61* \| \| *CYSTM1* \| \| *DAP* \| \| *DBI* \| \| *DCPS* \| \| *DDB2* \| \| *DDX11* \| \| *DEGS2* \| \| *DENND1A* \| \| *DESI2* \| \| *DHCR7* \| \| *DHRS3* \| \| *DLG3* \| \| *DNAH2* \| \| *DNAJB9* \| \| *DNAJC1* \| \| *DNAJC3* \| \| *DRAM1* \| \| *DRD2* \| \| *DUSP4* \| \| *DUSP6* \| \| *DYNLT3* \| \| *E2F2* \| \| *EBP* \| \| *ECM1* \| \| *EDEM1* \| \| *EDEM3* \| \| *EEF2K* \| \| *EFNA3* \| \| *EFNA5* \| \| *EFNB1* \| \| *EHD2* \| \| *EIF2AK3* \| \| *EIF2S2* \| \| *EIF4EBP1* \| \| *ELOVL6* \| \| *EML2* \| \| *EMP2* \| \| *ENO2* \| \| *EPB41L1* \| \| *EPDR1* \| \| *EPHB3* \| \| *ERLEC1* \| \| *ERN2* \| \| *ERP44* \| \| *ESAM* \| \| *ESRP1* \| \| *ETFA* \| \| *ETV4* \| \| *ETV5* \| \| *EXPH5* \| \| *FA2H* \| \| *FADS2* \| \| *FAIM2* \| \| *FAM102B* \| \| *FAM107B* \| \| *FAM114A1* \| \| *FAM171A1* \| \| *FAM213A* \| \| *FAM3D* \| \| *FAM81A* \| \| *FAM84B* \| \| *FASN* \| \| *FAT1* \| \| *FBXO2* \| \| *FBXO32* \| \| *FCMR* \| \| *FDFT1* \| \| *FDPS* \| \| *FERMT1* \| \| *FFAR4* \| \| *FGFBP1* \| \| *FGFR2* \| \| *FIBCD1* \| \| *FICD* \| \| *FLNB* \| \| *FLNC* \| \| *FMOD* \| \| *FOXA3* \| \| *FOXQ1* \| \| *FRMD3* \| \| *FRMPD1* \| \| *FSCN1* \| \| *FUT3* \| \| *FUT8* \| \| *FUT9* \| \| *FZD5* \| \| *GAL3ST4* \| \| *GALK2* \| \| *GALNT1* \| \| *GCAT* \| \| *GDPD2* \| \| *GFPT1* \| \| *GFPT2* \| \| *GLI3* \| \| *GLO1* \| \| *GMDS* \| \| *GMPPA* \| \| *GNAZ* \| \| *GOT1* \| \| *GPRC5A* \| \| *GPRC5B* \| \| *GPT2* \| \| *GPX2* \| \| *GREM1* \| \| *GRHL3* \| \| *GSKIP* \| \| *GSTM2* \| \| *GSTM3* \| \| *GSTM4* \| \| *H1F0* \| \| *H2AFJ* \| \| *HAUS4* \| \| *HEG1* \| \| *HERPUD1* \| \| *HES2* \| \| *HGSNAT* \| \| *HID1* \| \| *HIP1R* \| \| *HK2* \| \| *HLA-DMA* \| \| *HLA-DMB* \| \| *HMGCR* \| \| *HMGCS2* \| \| *HPDL* \| \| *HS3ST2* \| \| *HSD17B12* \| \| *HSPA13* \| \| *HSPA2* \| \| *HTATSF1* \| \| *HYAL1* \| \| *HYAL3* \| \| *IARS* \| \| *ICA1* \| \| *IDH2* \| \| *IDI1* \| \| *IFFO2* \| \| *IGF1R* \| \| *IGFBP4* \| \| *IL17RD* \| \| *IL1RN* \| \| *IL33* \| \| *IMPA2* \| \| *IMPDH1* \| \| *INHA* \| \| *INO80C* \| \| *IQGAP2* \| \| *ITGA1* \| \| *ITGA5* \| \| *ITGA6* \| \| *ITGB4* \| \| *ITGB7* \| \| *ITGBL1* \| \| *IVNS1ABP* \| \| *KALRN* \| \| *KCNE3* \| \| *KCNJ4* \| \| *KCNK5* \| \| *KCNN4* \| \| *KCTD12* \| \| *KCTD14* \| \| *KDELC2* \| \| *KDELR2* \| \| *KDELR3* \| \| *KDM7A-DT* \| \| *KIF12* \| \| *KIF13A* \| \| *KIF2C* \| \| *KIFC3* \| \| *KLF12* \| \| *KLF2* \| \| *KLF4* \| \| *KLHDC7A* \| \| *KLHL3* \| \| *KRT15* \| \| *KRT17* \| \| *KYAT1* \| \| *LAMA4* \| \| *LARS* \| \| *LCK* \| \| *LCN2* \| \| *LDHA* \| \| *LDHD* \| \| *LDLR* \| \| *LGALS1* \| \| *LGMN* \| \| *LIMCH1* \| \| *LINC00173* \| \| *LINC00239* \| \| *LINC00261* \| \| *LINC00482* \| \| *LINC01184* \| \| *LINC01315* \| \| *LINC01843* \| \| *LINC02320* \| \| *LIPG* \| \| *LMAN1* \| \| *LOXL1* \| \| *LOXL1-AS1* \| \| *LOXL4* \| \| *LPCAT4* \| \| *LPIN1* \| \| *LRP1* \| \| *LRP11* \| \| *LSS* \| \| *LTBP3* \| \| *LY6D* \| \| *LY6G6C* \| \| *LYPD6B* \| \| *LYZ* \| \| *MANSC1* \| \| *MAP3K5* \| \| *MAPRE2* \| \| *MARVELD1* \| \| *MBOAT1* \| \| *MCU* \| \| *ME1* \| \| *ME3* \| \| *MESD* \| \| *MFSD4A* \| \| *MGAT5* \| \| *MGST2* \| \| *MIR210HG* \| \| *MKNK2* \| \| *MLKL* \| \| *MLPH* \| \| *MMAB* \| \| *MPRIP* \| \| *MRPS6* \| \| *MSI2* \| \| *MSN* \| \| *MTCL1* \| \| *MTFR1* \| \| *MTSS1* \| \| *MUC1* \| \| *MUC12* \| \| *MUC17* \| \| *MUC2* \| \| *MUC4* \| \| *MUC5AC* \| \| *MVB12B* \| \| *MVD* \| \| *MYBPC1* \| \| *MYCN* \| \| *MYDGF* \| \| *MYEOV* \| \| *MYOF* \| \| *N4BP3* \| \| *NANS* \| \| *NDNF* \| \| *NDRG2* \| \|  \| \| *NEBL* \| \| *NEDD9* \| \| *NEK6* \| \| *NFATC4* \| \| *NFE2L3* \| \| *NFIB* \| \| *NIPAL3* \| \| *NMU* \| \| *NMUR2* \| \| *NOL3* \| \| *NOSTRIN* \| \| *NR3C1* \| \| *NR4A1* \| \| *NT5DC2* \| \| *NTN4* \| \| *NUCB2* \| \| *NUF2* \| \| *NUPR1* \| \| *NYNRIN* \| \| *OARD1* \| \| *OAS1* \| \| *OAS3* \| \| *OASL* \| \| *ODAM* \| \| *OSBP2* \| \| *OSBPL10* \| \| *OSBPL7* \| \| *OSTC* \| \| *OTOP3* \| \| *OTULINL* \| \| *P3H2* \| \| *P4HA1* \| \| *P4HB* \| \| *PACSIN2* \| \| *PAG1* \| \| *PAQR8* \| \|  \| \| *PC* \| \| *PCSK6* \| \| *PCSK9* \| \| *PDE10A* \| \| *PDE4C* \| \| *PDE4D* \| \| *PDIA3* \| \| *PDIA5* \| \| *PDK1* \| \| *PEX11A* \| \| *PFKFB2* \| \| *PFKFB3* \| \| *PGM1* \| \| *PGM3* \| \| *PHLDB2* \| \| *PHYH* \| \| *PI3* \| \| *PIGR* \| \| *PIR* \| \| *PITPNM3* \| \| *PKP2* \| \| *PLA2G2A* \| \| *PLA2G4A* \| \| *PLAC8* \| \| *PLBD1* \| \| *PLCB4* \| \| *PLCE1* \| \| *PLCL2* \| \| *PLEKHM1* \| \| *PLIN2* \| \| *PLOD2* \| \| *PLTP* \| \| *PLXNA2* \| \| *PMP22* \| \| *PNMA1* \| \| *POC1B* \| \| *POF1B* \| \| *PPARG* \| \| *PPFIA4* \| \| *PPFIBP2* \| \| *PPIB* \| \| *PPM1L* \| \| *PPP1R14D* \| \| *PPP1R1B* \| \| *PPP1R3B* \| \| *PPP1R3E* \| \| *PPP2R5C* \| \| *PRDM1* \| \| *PREP* \| \| *PRKAB1* \| \| *PRKCA* \| \| *PROM1* \| \| *PRSS12* \| \| *PRSS3* \| \| *PTPRN2* \| \| *PYCR1* \| \| *QPCT* \| \| *QPRT* \| \| *RAB15* \| \| *RAB6B* \| \| *RASA1* \| \| *RASAL1* \| \| *RASSF3* \| \| *REG4* \| \| *RFLNA* \| \| *RGS2* \| \| *RIMKLA* \| \| *RIN2* \| \| *RNASE1* \| \| *RNF125* \| \| *RNF144B* \| \| *RNF165* \| \| *RNF207* \| \| *RNF24* \| \| *ROR1* \| \| *RPL36AL* \| \| *RPN2* \| \| *RSPH1* \| \| *RTN3* \| \| *RXFP4* \| \| *S100A10* \| \| *S100A4* \| \| *S100P* \| \| *SAT1* \| \| *SAV1* \| \| *SBDS* \| \| *SC5D* \| \| *SCD* \| \| *SCEL* \| \| *SCPEP1* \| \| *SDR16C5* \| \| *SEC11C* \| \| *SEC24D* \| \| *SEC61G* \| \| *SEC63* \| \| *SEMA5A* \| \| *SERP1* \| \| *SERPINB5* \| \| *SFMBT2* \| \| *SH2D1B* \| \| *SH3BP1* \| \| *SH3GL2* \| \| *SHF* \| \| *SIDT2* \| \| *SIGLEC15* \| \| *SIPA1L2* \| \| *SLC12A2* \| \| *SLC12A8* \| \| *SLC13A5* \| \| *SLC14A1* \| \| *SLC16A1* \| \| *SLC17A9* \| \| *SLC1A4* \| \| *SLC28A2* \| \| *SLC29A3* \| \| *SLC2A1* \| \| *SLC2A10* \| \| *SLC2A13* \| \| *SLC31A1* \| \| *SLC35A3* \| \| *SLC35E4* \| \| *SLC38A4* \| \| *SLC38A5* \| \| *SLC39A7* \| \| *SLC39A8* \| \| *SLC43A1* \| \| *SLC43A3* \| \| *SLC45A3* \| \| *SLC4A3* \| \| *SLC50A1* \| \| *SLC52A3* \| \| *SLC5A1* \| \| *SLC6A6* \| \| *SLC7A1* \| \| *SLC7A11* \| \| *SLCO2B1* \| \| *SLITRK6* \| \| *SLPI* \| \| *SMOC1* \| \| *SMOX* \| \| *SNCG* \| \| *SND1* \| \| *SNHG18* \| \| *SNTA1* \| \| *SOD3* \| \| *SOWAHC* \| \| *SPAG4* \| \| *SPDEF* \| \| *SPINK4* \| \| *SPINK5* \| \| *SPOCK2* \| \| *SPON2* \| \| *SPRY1* \| \| *SPTLC2* \| \| *SQLE* \| \| *SREBF1* \| \| *SRGAP3* \| \| *SSBP3* \| \| *SSR1* \| \| *SSR2* \| \| *SSR3* \| \| *ST3GAL1* \| \| *ST3GAL2* \| \| *ST3GAL4* \| \| *ST6GAL1* \| \| *ST6GAL2* \| \| *ST6GALNAC6* \| \| *STARD10* \| \| *STC1* \| \| *STN1* \| \| *STON2* \| \| *STT3A* \| \| *STYK1* \| \| *SULT1B1* \| \| *SULT1C3* \| \| *SURF4* \| \| *SYNE3* \| \| *SYNPO* \| \| *SYT7* \| \| *SYTL4* \| \| *TACC1* \| \| *TAGLN2* \| \| *TBC1D8* \| \| *TBL1X* \| \| *TC2N* \| \| *TCEA3* \| \| *TCF7* \| \| *TCN1* \| \| *TESC* \| \| *TFF1* \| \| *TFF2* \| \| *TFF3* \| \| *TGFBI* \| \| *TGFBR3* \| \| *TIMP3* \| \| *TINCR* \| \| *TLN2* \| \| *TM4SF1* \| \| *TMBIM1* \| \| *TMC5* \| \| *TMC7* \| \| *TMED9* \| \| *TMEM141* \| \| *TMEM200B* \| \| *TMEM214* \| \| *TMEM229B* \| \| *TMEM238L* \| \| *TMEM246* \| \| *TMEM39A* \| \| *TMEM45B* \| \| *TMEM63C* \| \| *TMEM74B* \| \| *TMEM92* \| \| *TMTC2* \| \| *TNFRSF1B* \| \| *TNFRSF21* \| \| *TNFSF15* \| \| *TNS4* \| \| *TOB1* \| \| *TOR1B* \| \| *TOX2* \| \| *TPM4* \| \| *TPPP* \| \| *TRABD2A* \| \| *TRAM2* \| \| *TRIB3* \| \| *TRIM2* \| \| *TRIM29* \| \| *TRIM31* \| \| *TRIM7* \| \| *TSC22D1* \| \| *TSPAN11* \| \| *TSPAN13* \| \| *TSPAN2* \| \| *TSPAN4* \| \| *TSPAN5* \| \| *TTK* \| \| *TTPA* \| \| *TUSC8* \| \| *TXNDC15* \| \| *UAP1* \| \| *UBASH3B* \| \| *UBE2J1* \| \| *UBXN10* \| \| *UCP2* \| \| *UHRF1BP1* \| \| *UNC13A* \| \| *UPK3B* \| \| *UTP20* \| \| *VSIG2* \| \| *VSIR* \| \| *VSTM2L* \| \| *VSTM5* \| \| *VTCN1* \| \| *WFDC21P* \| \| *WNK4* \| \| *XBP1* \| \| *XPOT* \| \| *YARS* \| \| *ZDHHC11B* \| \| *ZDHHC8P1* \| \| *ZFP36L2* \| \| *ZG16B* \| \| *ZNF219* \| \| *ZNF385A* \| \| *ZNF385A* \| |
| --- | --- | --- | --- | --- | --- | --- | --- | --- | --- | --- | --- | --- | --- | --- | --- | --- | --- | --- | --- | --- | --- | --- | --- | --- | --- | --- | --- | --- | --- | --- | --- | --- | --- | --- | --- | --- | --- | --- | --- | --- | --- | --- | --- | --- | --- | --- | --- | --- | --- | --- | --- | --- | --- | --- | --- | --- | --- | --- | --- | --- | --- | --- | --- | --- | --- | --- | --- | --- | --- | --- | --- | --- | --- | --- | --- | --- | --- | --- | --- | --- | --- | --- | --- | --- | --- | --- | --- | --- | --- | --- | --- | --- | --- | --- | --- | --- | --- | --- | --- | --- | --- | --- | --- | --- | --- | --- | --- | --- | --- | --- | --- | --- | --- | --- | --- | --- | --- | --- | --- | --- | --- | --- | --- | --- | --- | --- | --- | --- | --- | --- | --- | --- | --- | --- | --- | --- | --- | --- | --- | --- | --- | --- | --- | --- | --- | --- | --- | --- | --- | --- | --- | --- | --- | --- | --- | --- | --- | --- | --- | --- | --- | --- | --- | --- | --- | --- | --- | --- | --- | --- | --- | --- | --- | --- | --- | --- | --- | --- | --- | --- | --- | --- | --- | --- | --- | --- | --- | --- | --- | --- | --- | --- | --- | --- | --- | --- | --- | --- | --- | --- | --- | --- | --- | --- | --- | --- | --- | --- | --- | --- | --- | --- | --- | --- | --- | --- | --- | --- | --- | --- | --- | --- | --- | --- | --- | --- | --- | --- | --- | --- | --- | --- | --- | --- | --- | --- | --- | --- | --- | --- | --- | --- | --- | --- | --- | --- | --- | --- | --- | --- | --- | --- | --- | --- | --- | --- | --- | --- | --- | --- | --- | --- | --- | --- | --- | --- | --- | --- | --- | --- | --- | --- | --- | --- | --- | --- | --- | --- | --- | --- | --- | --- | --- | --- | --- | --- | --- | --- | --- | --- | --- | --- | --- | --- | --- | --- | --- | --- | --- | --- | --- | --- | --- | --- | --- | --- | --- | --- | --- | --- | --- | --- | --- | --- | --- | --- | --- | --- | --- | --- | --- | --- | --- | --- | --- | --- | --- | --- | --- | --- | --- | --- | --- | --- | --- | --- | --- | --- | --- | --- | --- | --- | --- | --- | --- | --- | --- | --- | --- | --- | --- | --- | --- | --- | --- | --- | --- | --- | --- | --- | --- | --- | --- | --- | --- | --- | --- | --- | --- | --- | --- | --- | --- | --- | --- | --- | --- | --- | --- | --- | --- | --- | --- | --- | --- | --- | --- | --- | --- | --- | --- | --- | --- | --- | --- | --- | --- | --- | --- | --- | --- | --- | --- | --- | --- | --- | --- | --- | --- | --- | --- | --- | --- | --- | --- | --- | --- | --- | --- | --- | --- | --- | --- | --- | --- | --- | --- | --- | --- | --- | --- | --- | --- | --- | --- | --- | --- | --- | --- | --- | --- | --- | --- | --- | --- | --- | --- | --- | --- | --- | --- | --- | --- | --- | --- | --- | --- | --- | --- | --- | --- | --- | --- | --- | --- | --- | --- | --- | --- | --- | --- | --- | --- | --- | --- | --- | --- | --- | --- | --- | --- | --- | --- | --- | --- | --- | --- | --- | --- | --- | --- | --- | --- | --- | --- | --- | --- | --- | --- | --- | --- | --- | --- | --- | --- | --- | --- | --- | --- | --- | --- | --- | --- | --- | --- | --- | --- | --- | --- | --- | --- | --- | --- | --- | --- | --- | --- | --- | --- | --- | --- | --- | --- | --- | --- | --- | --- | --- | --- | --- | --- | --- | --- | --- | --- | --- | --- | --- | --- | --- | --- | --- | --- | --- | --- | --- | --- | --- | --- | --- | --- | --- | --- | --- | --- | --- | --- | --- | --- | --- | --- | --- | --- | --- | --- | --- | --- | --- | --- | --- | --- | --- | --- | --- | --- | --- | --- | --- | --- | --- | --- | --- | --- | --- | --- | --- | --- | --- | --- | --- | --- | --- | --- | --- | --- | --- | --- | --- | --- | --- | --- | --- | --- | --- | --- | --- | --- | --- | --- | --- | --- | --- | --- | --- | --- | --- | --- | --- | --- | --- | --- | --- | --- | --- | --- | --- | --- | --- | --- | --- | --- | --- | --- | --- | --- | --- | --- | --- | --- | --- | --- | --- | --- | --- | --- | --- | --- | --- | --- | --- | --- | --- | --- | --- | --- | --- | --- | --- | --- | --- | --- | --- | --- | --- | --- | --- | --- | --- | --- | --- | --- | --- | --- | --- | --- | --- | --- | --- | --- | --- | --- | --- | --- | --- | --- | --- | --- | --- | --- | --- | --- | --- | --- | --- | --- | --- | --- | --- | --- | --- | --- | --- | --- | --- | --- | --- | --- | --- | --- | --- | --- | --- | --- | --- | --- | --- | --- | --- | --- | --- | --- | --- | --- | --- | --- | --- | --- | --- | --- | --- | --- | --- | --- | --- | --- | --- | --- | --- | --- | --- | --- | --- | --- | --- | --- | --- | --- | --- | --- | --- | --- | --- | --- | --- | --- | --- | --- | --- | --- | --- | --- | --- | --- | --- | --- | --- | --- | --- | --- | --- | --- | --- | --- | --- | --- | --- | --- | --- | --- | --- | --- | --- | --- | --- | --- | --- | --- | --- | --- | --- | --- | --- | --- | --- | --- | --- | --- | --- | --- | --- | --- | --- | --- | --- | --- | --- | --- | --- | --- | --- | --- | --- | --- | --- | --- | --- | --- | --- | --- | --- | --- | --- | --- | --- | --- | --- | --- | --- | --- | --- | --- | --- | --- | --- | --- | --- | --- | --- | --- | --- | --- | --- | --- | --- | --- | --- | --- | --- | --- | --- | --- | --- | --- | --- | --- | --- | --- | --- | --- | --- | --- | --- | --- | --- | --- | --- | --- | --- | --- | --- | --- | --- | --- | --- | --- | --- | --- | --- | --- | --- | --- | --- | --- | --- | --- | --- | --- | --- | --- | --- | --- | --- | --- | --- | --- | --- | --- | --- | --- | --- | --- | --- | --- | --- | --- | --- | --- | --- | --- | --- | --- | --- | --- | --- | --- | --- | --- | --- | --- | --- | --- | --- | --- | --- | --- | --- | --- | --- | --- | --- | --- | --- | --- | --- | --- | --- | --- | --- | --- | --- | --- | --- | --- | --- | --- | --- | --- | --- | --- | --- | --- | --- | --- | --- | --- | --- | --- | --- | --- | --- | --- | --- | --- | --- | --- | --- | --- | --- | --- | --- | --- | --- | --- | --- | --- | --- | --- | --- | --- | --- | --- | --- | --- | --- | --- | --- | --- | --- | --- | --- | --- | --- | --- | --- | --- | --- | --- | --- | --- | --- | --- | --- | --- | --- | --- | --- | --- | --- | --- | --- | --- | --- | --- | --- | --- | --- | --- | --- | --- | --- | --- | --- | --- | --- | --- | --- | --- | --- | --- | --- | --- | --- | --- | --- | --- | --- | --- | --- | --- | --- | --- | --- | --- | --- | --- | --- | --- | --- | --- | --- | --- | --- | --- | --- | --- | --- | --- | --- | --- | --- | --- | --- | --- | --- | --- | --- | --- | --- | --- | --- | --- | --- | --- | --- | --- | --- | --- | --- | --- | --- | --- | --- | --- | --- | --- | --- | --- | --- | --- | --- | --- | --- | --- | --- | --- | --- | --- | --- | --- | --- | --- | --- | --- | --- | --- | --- | --- | --- | --- | --- | --- | --- | --- | --- | --- | --- | --- | --- | --- | --- | --- | --- | --- | --- | --- | --- | --- | --- | --- | --- | --- | --- | --- | --- | --- | --- | --- | --- | --- | --- | --- | --- | --- | --- | --- | --- | --- | --- | --- | --- | --- | --- | --- | --- | --- | --- | --- | --- | --- | --- | --- | --- | --- | --- | --- | --- | --- | --- | --- | --- | --- | --- | --- | --- | --- | --- | --- | --- | --- | --- | --- | --- | --- | --- | --- | --- | --- | --- | --- | --- | --- | --- | --- | --- | --- | --- | --- | --- | --- | --- | --- | --- | --- | --- | --- | --- | --- | --- | --- | --- | --- | --- | --- | --- | --- | --- | --- | --- | --- | --- | --- | --- | --- | --- | --- | --- | --- | --- | --- | --- | --- | --- | --- | --- | --- | --- | --- | --- | --- | --- | --- | --- | --- | --- | --- | --- | --- | --- | --- | --- | --- | --- | --- | --- | --- | --- | --- | --- | --- | --- | --- | --- | --- | --- | --- | --- | --- | --- | --- | --- | --- | --- | --- | --- | --- | --- | --- | --- | --- | --- | --- | --- | --- | --- | --- | --- | --- | --- | --- | --- | --- | --- | --- | --- | --- | --- | --- | --- | --- | --- | --- | --- | --- | --- | --- | --- | --- | --- | --- | --- | --- | --- | --- | --- | --- | --- | --- | --- | --- | --- | --- | --- | --- | --- | --- | --- | --- | --- | --- | --- | --- | --- | --- | --- | --- | --- | --- | --- | --- | --- | --- | --- | --- | --- | --- | --- | --- | --- | --- | --- | --- | --- | --- | --- | --- | --- | --- | --- | --- | --- | --- | --- | --- | --- | --- | --- | --- | --- | --- | --- | --- | --- | --- | --- | --- | --- | --- | --- | --- | --- | --- | --- | --- | --- | --- | --- | --- | --- |

Red, genes upregulated

Blue, genes downregulated
